# Iron oxides and aluminous clays selectively control soil carbon storage and stability in the humid tropics

**DOI:** 10.1038/s41598-021-84777-7

**Published:** 2021-03-03

**Authors:** Maximilian Kirsten, Robert Mikutta, Cordula Vogel, Aaron Thompson, Carsten W. Mueller, Didas N. Kimaro, Huig L. T. Bergsma, Karl-Heinz Feger, Karsten Kalbitz

**Affiliations:** 1 Technische Universität Dresden, Institute of Soil Science and Site Ecology, Tharandt, Germany; 2 Soil Science and Soil Protection, Martin Luther University Halle-Wittenberg, Halle (Saale), Germany; 3 Department of Crop and Soil Sciences, University of Georgia, Athens, GA USA; 4 Department of Geosciences and Natural Resource Management, University of Copenhagen, Copenhagen, Denmark; 5 Directorate of Research Innovations and Consultancy, Mwenge Catholic University, Moshi, Tanzania; 6 BodemBergsma, Blikakker 8, 7421 GD Deventer, The Netherlands

**Keywords:** Biogeochemistry, Environmental sciences

## Abstract

Clay minerals and pedogenic metal (oxyhydr)oxides are the most reactive soil mineral constituents controlling the long-term persistence of organic carbon (OC) in terrestrial ecosystems. However, their co-occurrence in most soils complicates direct assessment of their individual contribution to OC persistence. Making use of unique mineralogical combinations in soils located in the East Usambara Mountains of Tanzania, we disentangled the contribution of clay-sized aluminous minerals (kaolinite, gibbsite) and pedogenic Fe (oxyhydr)oxides (predominant goethite and hematite) on OC storage and stabilization under natural forests and croplands. Topsoil samples, varying in contents but not types of aluminous clays and pedogenic Fe (oxyhydr)oxides, were identified by selective extractions, X-ray diffraction, and Mössbauer spectroscopy. Associated abundance of particulate and mineral-associated organic matter (OM) was quantified by density fractionation and their changes during land-use conversion were determined as a measure of OC persistence. Additionally, we assessed the resistance of OC to chemical oxidation as well as microbial decomposition in a 50-day laboratory incubation. We found that the ratio of pedogenic Fe to aluminous clay is more consequential for OC storage and stabilization than their individual contents, despite the fact that Fe (oxyhydr)oxides generally exert a stronger impact on OC than aluminous clays. Conjunction of large amounts of Fe (oxyhydr)oxides with low aluminous clay contents caused the strongest accumulation of mineral-associated OC, a low soil respiration, high OC stability against chemical oxidation, and high OC persistence during land-use change. Our study suggests that certain mineralogical combinations in the humid tropics alleviate OM losses during land conversion because of the strong and selective mineral control on OC stabilization, particular if the weight ratio of pedogenic Fe to aluminous clay exceeds the threshold range of 0.44‒0.56.

## Introduction

Soils are the largest terrestrial reservoir of biologically fixed atmospheric carbon dioxide, comprising 3500–4800 Pg of organic carbon (OC)^1^. Climate^2^ and geological parent material^3^ are well-known controls on the long-term storage and stabilization of OC. In contrast to these natural environmental factors, land-use change has a direct impact on OC storage, which can obscure the effects of climate, geology, and geochemistry^4^. Sub Saharan Africa is known for ongoing land-use change, because of population growth and increasing demand for food^5^. It is widely accepted that forest to cropland conversion results in carbon loss, threatening the key role of soils in ecosystem services^6^. Uncertainty about the magnitude of OC losses have a huge impact for African societies because the biogeochemical function of tropical soils is highly depending on organic matter (OM). One crucial limitation in our understanding of the carbon cycle in tropical soils is that we are not clear which processes and mechanisms drive OC storage in response to altered land use, especially in soils of Sub-Saharan Africa^7^. In fact, the change in OC storage as tropical soils are converted from forest to cropland exhibit tremendous variability, ranging from a loss of 80% to a gain of 58%^4^, while the controlling factors for this variation remain obscure.

**Figure 1:**
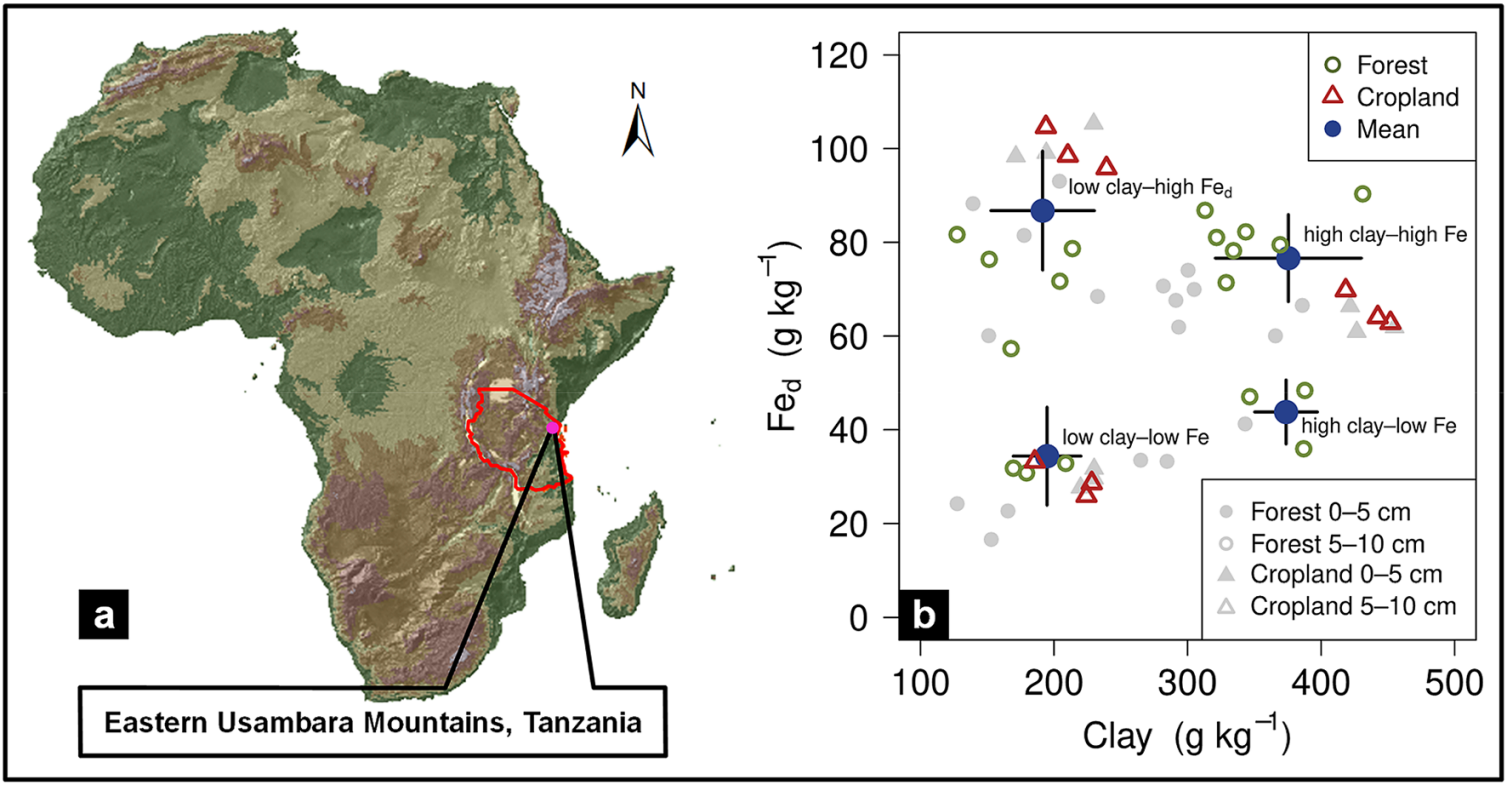
(**a**) Study site location in the Eastern Usambara Mountains of Tanzania. This map was created using the software ArcGIS version 10.3; https://desktop.arcgis.com/de/arcmap/; (**b**) total sample set showing the combinations of aluminous clay (clay) and pedogenic Fe oxides (Fe_d_) at the study site (*n* = 54). Aluminous clay represents the weight sum of kaolinite and gibbsite present in the < 2-µm fraction, and pedogenic Fe oxides are represented by dithionite-bicarbonate-citrate-extractable Fe. The depth of 5–10 cm (open colored circles and triangles) was used to assign samples to one of the mineralogical combinations. Large blue dots show the respective combination means, while whiskers represent the associated standard deviations.

Organic matter associated with pedogenic minerals contributes largely to total OM in soil^2,8^ and frequently exhibits lower decomposition rates than plant residues^9–11^. The formation of mineral-associated OM through biogeochemical interactions between mineral particles and OM^12^ is regarded as one preeminent factor for the preservation of soil OC^13,14^. Secondary pedogenic minerals formed during the weathering like aluminum (Al) and iron (Fe) (oxyhydr)oxides (hereafter termed ‛oxides’), as well as clay minerals are proposed as the main soil constituents binding OM^14–16^. Kaolinite (Al_2_Si_2_O_5_(OH)_4_), gibbsite (γ-Al(OH)_3_), goethite (α-FeOOH), and hematite (α-Fe_2_O_3_) are typical minerals in weathered tropical soils. Their functional groups are reactive toward OM under the acidic pH conditions common in weathered tropical soils^15,17^, and this enables the sorptive stabilization of OM^18,19^. In addition, microorganisms encounter favorable conditions (temperature, soil moisture) for OM decomposition under humid tropical climate. This reduces the molecular size of biomolecules and adds oxygen-containing functional groups to OM, both processes being essential for the interaction of OM with soil minerals^20^. As both clay and pedogenic Fe and Al oxides increases in soils, the abundance and stocks of OC also typically increase in tropical ecosystems and beyond^15,21,22^. Yet, it is still an open question whether pedogenic Fe oxides are more efficient at stabilizing OM than other clay-sized mineral phases in tropical soils, and further, which mineralogical properties control the vulnerability of mineral-associated OC (MAOC) to microbial decomposition after land-use change. It has to be considered that the clay-sized fraction (< 2-µm) as basis for the observed correlations to OC, does not only comprise different types of clay minerals, but also variable forms and amounts of pedogenic Fe and Al oxides^23,24^. This co-occurrence of mineral phases in the clay fraction makes it challenging to identify the independent role of these mineral constituents. In tropical soils, secondary aluminous clays, such as gibbsite and kaolinite, form a second group of mineral constituents in addition to Fe oxides, which are highly related to each other. Gibbsite as the most important Al hydroxide has a similar structure as the octahedral layer of kaolinite^25^ and might form upon desilication of kaolinite^26–28^ or even be transformed back to kaolinite through resilication^29,30^. Studying the selective contribution of these aluminous clays and pedogenic Fe oxides to OM stabilization in soils under field conditions thus requires distinct combinations in the content of both mineralogical groups under similar environmental conditions including climatic and geochemical properties. In this study, we make use of contrasting mineralogical combinations in soils of the East Usambara Mountains of Tanzania (Fig. 1a) to investigate how aluminous clays and pedogenic Fe oxides individually affect the storage of OC, in particular as MAOC. Since the soils include both natural forests and areas converted to croplands, we further explore how aluminous clay and pedogenic Fe oxides modulate OC persistence during land-use change. We evaluate the stability of MAOC by testing its susceptibility to chemical oxidation with sodium hypochlorite (NaOCl)^31^ and to microbial decomposition in an 50-day laboratory incubation experiment. These metrics of OC stability are then compared between land-use types to assess how OM stability is influenced by clay-sized aluminum phases and pedogenic Fe oxides.

## Results and discussion

### Mineralogical and geochemical properties of soil mineralogical combinations

Soil samples were collected from six forest and three cropland sites in the East Usambara Mountains at comparable mid-slope relief positions. We relied on topsoils (0‒5 cm and 5‒10 cm) since land-use-induced OC losses in this region largely occur in uppermost soil horizons^32^. The study area on the plateau of the East Usambara Mountains is characterized by mafic biotite-hornblende-garnet gneiss^33^, promoting the development of Acrisols and Alisols (Table S1)^32,34^. At these sites, tropical weathering resulted in an enrichment of secondary Fe and Al oxides over silica-bearing primary and secondary minerals, as indicated by low values of common weathering proxies (*K*_r_ = SiO_2_ / (Al_2_O_3_ + Fe_2_O_3_) and *K*_i_ = SiO_2_/Al_2_O_3_; Table S2). Soils had distinct combinations of aluminous clay (< 2-µm fraction after removal of OM and pedogenic Fe oxides) and pedogenic Fe oxides (dithionite-citrate-bicarbonate-extractable Fe; Fe_d_), but otherwise similar soil properties (Tables S1–S3). Four groups varying in aluminous clay and pedogenic Fe oxide contents were defined for the forest sites (i.e. ‛low clay‒low Fe’, ‛low clay‒high Fe’, ‛high clay‒low Fe’, ‛high clay‒high Fe’), whereas three groups could be selected for cropland sites based on the 5–10 cm depth increment (Fig. 1b; Table 1). The aluminous clay fraction from all sites displayed a remarkable homogeneous composition comprised of kaolinite and gibbsite, as indicated by distinct X-ray diffraction peaks at 7.13 Å (d001) and 3.56 Å (d002) for kaolinite and a reflection at 4.85 Å (d002) for gibbsite (Figure S1). Negligible amounts of oxalate-extractable Al (< 0.02% of total Al; Table S2) were present in all soils. Across the mineralogical combinations, goethite and hematite were the dominant Fe oxides as identified by Mössbauer spectroscopy (Figure S2). About 90% of the total Fe could be assigned to these two minerals, with goethite dominating over hematite (Table S3). This accords to common knowledge that tropical weathering promotes the development of well-ordered Fe oxides^35–37^. This is further underpinned by the high crystallinity index (> 0.79) calculated based on Mössbauer data^38^, and a low contribution of oxalate-extractable Fe (< 7% of Fe_d_; Table S2) throughout the mineralogical combinations. Magnetic susceptibility measurements further suggest the minor presence of maghemite and/or magnetite (Supplementary Material Sect. 3; Table S4).

**Table 1:** Mineralogical combinations and their related amount of aluminous clay (clay) and dithionite-citrate-bicarbonate-extractable Fe (Fe_d_), and respective Fe_d_ to aluminous clay ratios (Fe_d_/clay).

Land use	Mineralogical combination	Depth (cm)	Clay(g kg^‒1^)	Fe_d_ (g kg^‒1^)	Fe_d_/clay	Bulk OC	MAOC
Forest	Low aluminous clay‒	0‒5	149^b^ (19)	21^d^ (4)	0.15^b,A^ (0.04)	76.0^ab,A^ (27.4)	27.1^a,A^ (2.9)
	Low pedogenic Fe oxides	5‒10	181^b^ (19)	38^b^ (13)	0.21^bc,A^ (0.09)	34.1^a,A^ (6.2)	23.2^ab,A^ (5.5)
Forest	Low aluminous clay‒	0‒5	182^b^ (38)	78^a^ (14)	0.45^a,A^ (0.12)	57.3^b,A^ (14.4)	36.0^a,A^ (4.9)
	High pedogenic Fe oxides	5‒10	174^b^ (42)	77^a^ (4)	047^a,A^ (0.13)	37.2^a,A^ (6.6)	28.3^a,B^ (5.3)
Forest	High aluminous clay‒	0‒5	298^a^ (41)	36^c^ (5)	0.12^b^ (0.01)	43.2^b^ (6.1)	24.5^a^ (1.1)
	Low pedogenic Fe oxides	5‒10	374^a^ (24)	44^b^ (7)	0.12^c^ (0.02	23.0^b^ (5.0)	14.6^c^ (0.8)
Forest	High aluminous clay‒	0‒5	318^a^ (41)	67^b^ (5)	0.22^b,A^ (0.03)	95.1^a,A^ (31.1)	36.1^a,A^ (15.7)
	High pedogenic Fe oxides	5‒10	349^a^ (40)	81^a^ (6)	0.23^b,A^ (0.02)	34.9^a,A^ (4.5)	22.6^b,A^ (2.6)
Cropland	Low aluminous clay‒	0‒5	227^b^ (6)	30^c^ (2)	0.13^b,A^ (0.01)	18.7^c,B^ (0.2)	10.5^c,B^ (2.7)
	Low pedogenic Fe oxides	5‒10	213^b^ (24)	29^c^ (4)	0.14^b,A^ (0.03)	18.8^c,B^ (1.3)	13.7^c,B^ (0.2)
Cropland	Low aluminous clay‒	0‒5	198^b^ (29)	101^a^ (4)	0.51^a,A^ (0.06)	47.1^a,A^ (0.9)	38.2^a,A^ (4.7)
	High pedogenic Fe oxides	5‒10	215^b^ (23)	100^a^ (5)	0.47^a,A^ (0.07)	48.1^a,A^ (4.8)	36.7^a,A^ (1.3)
Cropland	High aluminous clay‒	0‒5	434^a^ (18)	63^b^ (3)	0.15^b,B^ (0.01)	33.7^b,B^ (1.2)	20.0^b,A^ (0.9)
	High pedogenic Fe oxides	5‒10	438^a^ (17)	66^b^ (4)	0.15^b,B^ (0.01)	28.9^b,A^ (2.9)	18.8^b,A^ (3.0)

Organic carbon contents are given for bulk soil (Bulk OC) and the heavy fraction (MAOC). Aluminous clay represents the weight sum of kaolinite and gibbsite present in the < 2-µm fraction. Lower case letter denote significant differences within a certain land use and soil depth; capital letters indicate significant differences between land-use types for a given mineralogical combination. Sample numbers for the combinations are as follows: ‛low clay‒low Fe’ under forest (n = 4), ‛low clay‒high Fe’ under forest (n = 4), ‛high clay‒low Fe’ under forest (n = 3), ‛high clay‒high Fe’ under forest (n = 7); all cropland combinations (n = 3); mean and standard deviation in parentheses.

The BET specific surface area (SSA) of heavy soil fractions after OM removal ranged between 10 to 32 and 17 to 36 m^2^ g^–1^ for the 0–5 and 5–10 cm depth increments (Table S7). Across the mineralogical combinations, the ‛low clay‒low Fe’ combination had a significantly lower SSA, being lower or similar to those of pure kaolinite (16 m^2^ g^–1^ ^31^). In the other combinations, increasing abundance of aluminous clay or pedogenic Fe oxides caused higher SSA values, but no clear differences between combinations were evident. The mineralogical composition of the studied soils is also reflected in important soil properties such as the effective cation exchange capacity (CEC_eff_), base saturation, and pH, which were in the typical range for weathered tropical topsoils (Table S2)^32^. The low CEC_eff_ (2.9‒9.4 cmol_c_ kg^‒1^) is representative for soils rich in low-activity clay minerals^35,38^. Cropland soils had significantly higher pH (4.8‒5.4) than the forest soils (3.5‒4.1), which can be attributed to management activities such as biomass burning. Agricultural management likely also explains the uniformly high base saturation of cropland soils (93‒98%), in contrast to the lower and more variable base saturation found in forest soils (8‒72%; Table S2). Likewise, the composition of organic input materials (plant litter) differed to some extent between both land uses. While cropland litter contained more O/N-alkyl carbons (cellulose and hemicelluloses^39^), forest litter comprised more carboxyl and aryl carbons, e.g., derived from lignin^40^ (Figure S3; Table S8). However, both litter materials were dominated by O/N-alkyl carbons and exhibited a low and comparable degree of decomposition, as indicated by similar alkyl to O/N-alkyl carbon ratios (Table S8).

Summing up, our analyses confirm that soils across the mineralogical combinations have the same minerals present, but those minerals vary in abundance. Thus, we can consider the weight contribution of aluminous clays and pedogenic Fe oxides as the key predictor for differences in OC storage and stability.

### Dependence of bulk soil carbon on aluminous clay and pedogenic Fe oxides

**Figure 2:**
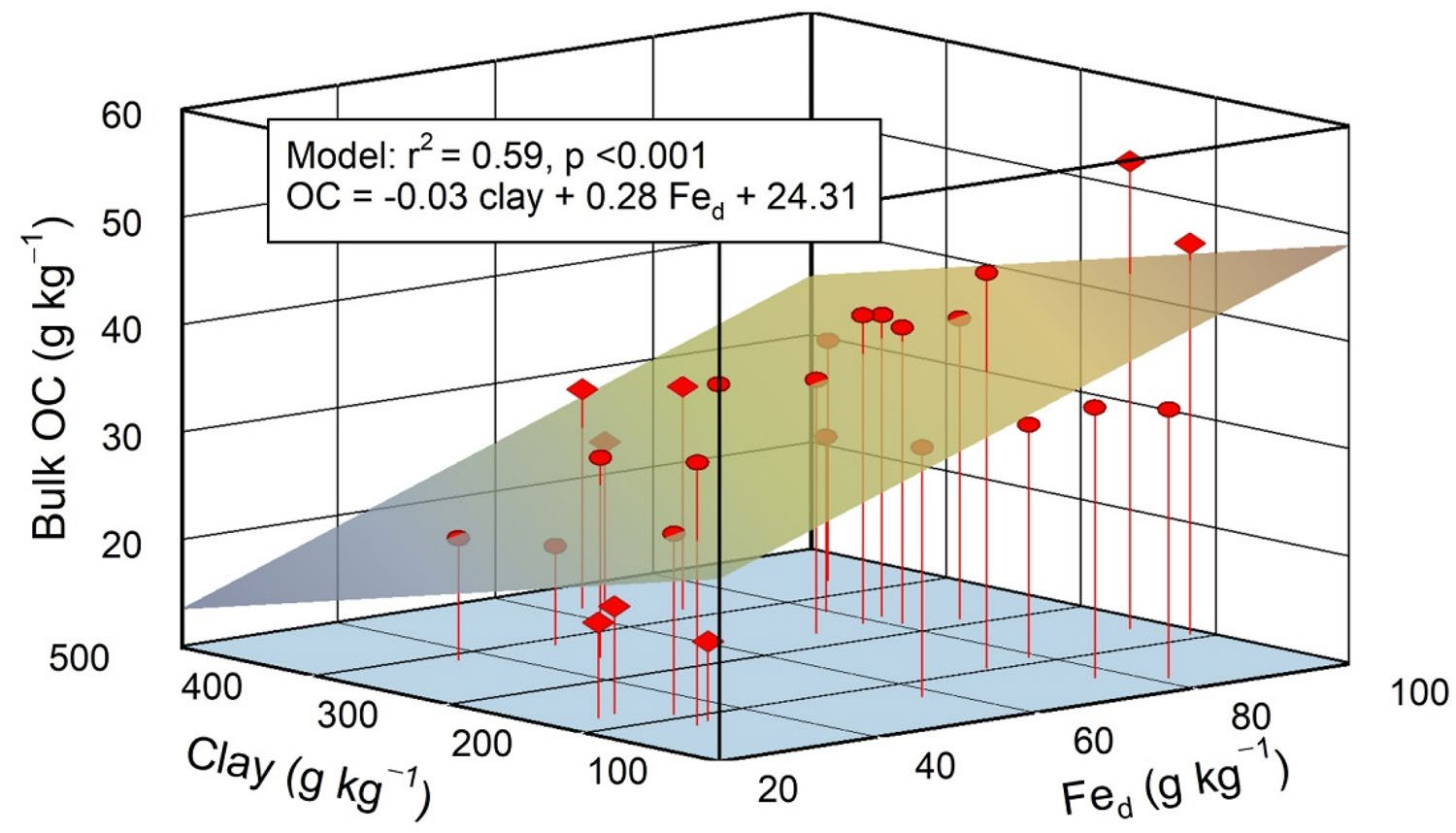
Multiple linear regression of bulk OC content in relation to aluminous clay (clay) and pedogenic Fe oxides (Fe_d_) contents for all mineralogical combinations in 5‒10 cm soil depth (*n* = 27). Circles and diamonds denote sites under forest and cropland, respectively. Aluminous clay represents the weight sum of kaolinite and gibbsite present in the < 2-µm fraction and Fe_d_ denotes the content of total pedogenic Fe extractable by dithionite-citrate-bicarbonate.

The different combinations in aluminous clay and pedogenic Fe oxides had a significant effect on bulk soil OC storage under both land uses (Table 1). Low contents in aluminous clay in combination with high pedogenic Fe oxide contents caused significantly higher bulk OC contents. Especially under cropland, we found a significant increase in bulk OC contents for both soil depths across mineralogical combinations in the order ‛low clay‒low Fe’ < ‛high clay‒high Fe’ < ‛low clay‒high Fe’. Doubling the Fe_d_ content while keeping the aluminous clay content low resulted in about 2.5-fold more bulk OC in 0‒5 cm of the croplands (i.e. 48 and 19 g kg^‒1^ for the ‛low clay‒high Fe’ and ‛low clay‒low Fe’ combinations, respectively; Table 1). The positive effect of pedogenic Fe oxides on bulk OC storage is likely due to their large affinity for the sorption of organic compounds under the acidic conditions^20,25,41^. Apparently, the surface and charge characteristics of the aluminous clays (kaolinite and gibbsite) rendered them less active in OC binding than those of pedogenic Fe oxides^37,42^. In laboratory sorption experiments under slightly acidic pH conditions, Gao et al.^43^ similarly observed about tenfold higher adsorption of dissolved OM to goethite than kaolinite. Furthermore, goethite coatings tripled the retention of dissolved OM by kaolinite^42^. An assessment of the relevance of short-range order (SRO) Fe minerals for OC storage in our study soils is only indirectly possible. Given the mass of mineral-associated OM (MAOC × 2) and assuming that the sorption capacity of SRO minerals, like nano-goethite or ferrihydrite (estimated as oxalate-extractable Fe × 1.7^44^), for OM can be close to their own mass^45,46^, we reason that the majority of MOAC in the studied soil has to be bound to crystalline Fe oxides or aluminous clay. This rough estimate, though, is consistent with the observation that in weathered tropical soils, SRO Fe minerals play a subordinate r ole for OC storage^47^. Our data did not show positive effects of increasing amounts in aluminous clay on bulk OC storage as observed for clay of kaolinitic Arenosols and Ferralsols under tropical forest in Ghana^48^. In contradiction to many other studies, aluminous clay contributed slightly negative to bulk OC contents, whereas Fe_d_ affected the bulk OC contents positively in the 5‒10 cm depth increment (r^2^ = 0.59, p < 0.01; Fig. 2, Table S6). This suggests that increasing aluminous clay contents do not necessarily imply higher bulk OC contents even in weathered tropical soils. The moderate explanatory power of the multiple regression model is likely due to the presence of plant residues (19‒64% of total OC), which mask the effect of mineral composition on bulk OC storage, particularly in 0‒5 cm depth (Table S6).

We calculated land-use-induced OC losses and gains for 10 cm soil depth based on bulk OC stocks of the two land-use systems measured in two depth increments (Table S5). Conversion of natural forest to cropland in the study area dates back at least > 15 years ago, thus, top soils likely approached a new equilibrium^4,17^. An intriguing persistence of bulk OC was observed for the ‛low clay‒high Fe’ combination. In fact, OC stocks were even 1.2 kg m^‒2^ higher under cropland than in the forest (Figure S4 and S5; Table S5), suggesting mineralogical combinations with the highest ratio of pedogenic Fe oxides to aluminous clay may have additional capacity to accumulate carbon (Table 1). In contrast, significant losses of bulk OC stocks induced by land use were observed in the other croplands with the largest loss of 3.5 kg m^‒2^ in mineralogical combination with low aluminous clay and low Fe contents (Figure S5). At present we can only speculate why the ‛low clay‒high Fe’ combination resulted in no loss of bulk OC during land-use change, while OC losses occurred in the ‛high clay‒high Fe’ combination. The proton dissociation constants of edge OH groups of kaolinite (pK_a_ = 6.9 and 5.7^20^) are much closer to the measured soil pH (3.5‒5.4; Table S2) than those of goethite and hematite (pK_a_ ≥ 7.7^20^). In Oxisols and Ultisols from Thailand, the point of zero charge of kaolinite was even lower and varied between 2.3 and 2.9^49^. That means under the given soil pH, pedogenic Fe oxides provide more positive net surface charge^31,41^ and a significant share of kaolinite might even be net negatively charged. Thus, with increasing kaolinite content (i.e. decreasing ratio of pedogenic Fe to aluminous clay), positive charges provided by pedogenic Fe oxides could be more effectively screened and neutralized, e.g. by forming coatings on kaolinite particles or Fe oxide-kaolinite aggregates^50^. Formation of coatings or aggregates might thus constrain available positive charges of pedogenic Fe oxides in order to bind and stabilize OM in the form of MAOC, leading to overall lower bulk OC content in the ‛high clay‒high Fe’ combination.

### Effects of aluminous clay and pedogenic Fe oxides on mineral-associated carbon

**Figure 3:**
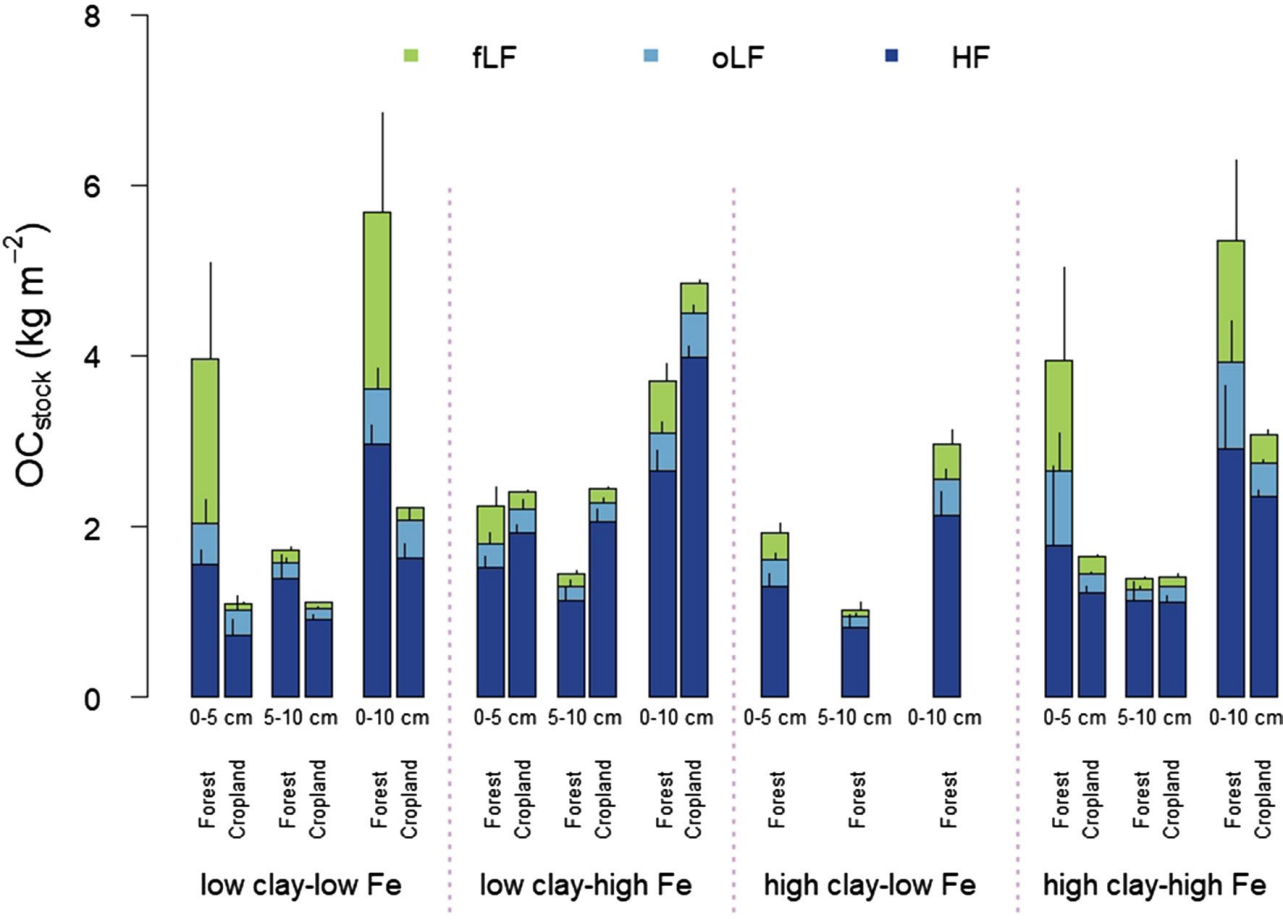
Bulk OC stocks related to soil density fractions (fLF and oLF = free and occluded light fraction; HF = heavy fraction) in mineralogical combinations under forest and cropland separated by soil depth. Sample numbers for the combinations are as follows: ‛low clay‒low Fe’ under forest (*n* = 4), ‛low clay‒high Fe’ under forest (*n* = 4), ‛high clay‒low Fe’ under forest (*n* = 3), ‛high clay‒high Fe’ under forest (*n* = 7); all cropland combinations (*n* = 3).

The amount of MAOC was determined by density fractionation and defined as OC associated with the heavy soil fraction^51–53^. In the studied topsoils, MAOC accounted for 36‒81% and 64‒76% of the bulk OC in the top 5 cm and 5‒10 cm depth, respectively. These ranges are much wider than values determined for sandy and clay-rich topsoils from Zimbabwe, where MAOC accounts for 86‒98% of total OC^48^. The high variation in MAOC, especially in the topmost 5 cm under forest, can be explained by the significantly higher and more variable amount of the free light fraction compared to the cropland soils (Table S5). As found for bulk OC, the combination of low aluminous clay and high Fe content was associated with significantly higher OC contents and stocks in MAOC under both land-use types and both sampling depths (Fig. 3; Table 1; Table S5). The positive effect of increasing pedogenic Fe to aluminous clay ratios on OC is even more pronounced for MAOC than for bulk OC as plant residues as interfering factor were removed by density fractionation^20,52,54^. We found significant relationships between MAOC, aluminous clay, and Fe_d_ contents at all depth increments (MAOC_0‒5 cm_: r^2^ = 0.18, p = 0.03; MAOC_5‒10 cm_: r^2^ = 0.68, p = 0.001; MAOC_0‒10 cm_: r^2^ = 0.28, p = 0.001; Table S6).

When differentiating between the two land-use regimes, we found that the explanatory power of Fe_d_ and aluminous clay, as well as the pedogenic Fe to aluminous clay ratio in the regression models were always stronger for the cropland than for the forest soils (Table S6). The pedogenic Fe to aluminous clay ratio, however, explained the variability of MAOC less well under forest, particularly in the upper depth increment. We speculate that is due to the reduced mechanical disturbance of forest topsoils, rendering mineral surfaces partly less accessible to OM compared to those of the managed croplands.

**Figure 4:**
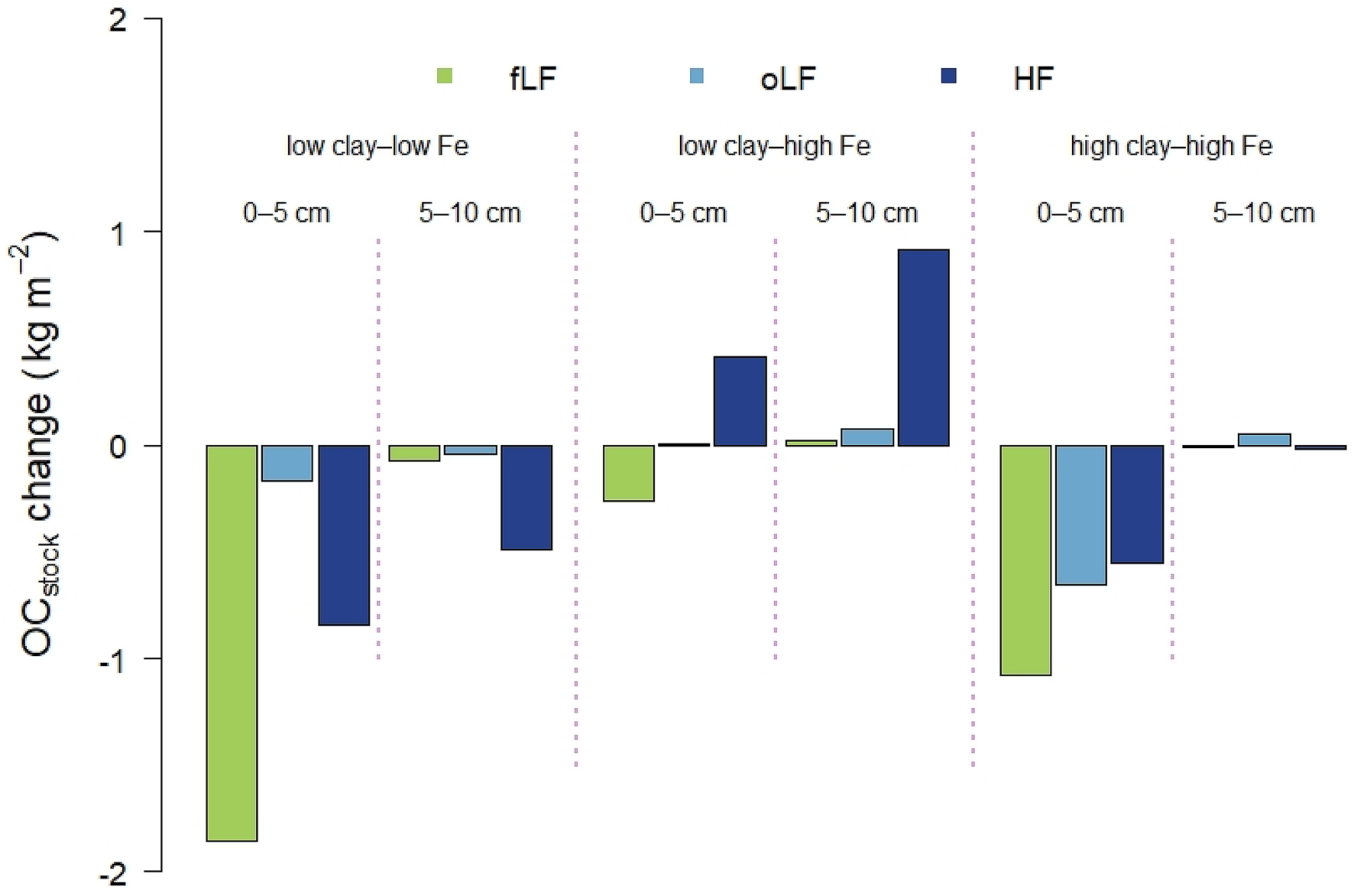
Changes of OC stocks associated with soil density fractions (fLF and oLF = free and occluded light fraction; HF = heavy fraction) in mineralogical combinations and separated by soil depth. Sample numbers for the combinations are as follows: ‛low clay‒low Fe’ under forest (*n* = 4), ‛low clay‒high Fe’ under forest (*n* = 4), ‛high clay‒low Fe’ under forest (*n* = 3), ‛high clay‒high Fe’ under forest (*n* = 7); all cropland combinations (*n* = 3).

The amount of MOAC changed significantly due to land-use conversion in the 0‒5 cm depth at low pedogenic Fe to aluminous clay ratios, i.e. for the ‛low clay‒low Fe’ and ‛high clay‒high Fe’ combinations. For these combinations, land-use change from forest to cropland caused a decline in MAOC contents of 17‒61%, accounting for maximum loss of 0.6 kg C m^‒2^ in the ‛low clay‒low Fe’ combination (Table S5). For 5‒10 cm depth, we found similar but statistically not significant relationships (Table S5). In the case of sandy and loamy soils in Zimbabwe, land-use change from forest to cropland under conventional tillage caused a loss in MAOC stocks of up to 1 kg m^‒2^ at 0‒30 cm depth^48^. In contrast, conversion of natural forest to pasture systems was accompanied by MAOC losses of 1.2 kg m^‒2^, but also minor gains of 0.15 kg m^‒2^ were observed in 0‒10 cm depth of sedimentary soils in northwestern Ecuador^55^. Noteworthy, this study included soils with a wide range of clay contents (287‒639 g kg^‒1^), but high clay content did not necessarily reduce the MAOC losses^55^. This also matches our finding that higher aluminous clay abundance does not necessarily attenuate MAOC losses upon land-use change. For the ‛low clay‒high Fe’ combination, the MAOC content and stock increased upon forest conversion to croplands at both depths (0.5 kg m^‒2^ in the 0‒5 cm depth, and 0.8 kg m^‒2^ in the 5‒10 cm depths, respectively; Table S5, Fig. 3). Thus, higher MAOC persistence in weathered tropical soils is evidently caused by a combination of low aluminous clay (kaolinite, gibbsite) and high pedogenic Fe oxide (goethite, hematite) contents. The observed persistence of MAOC at high pedogenic Fe to aluminous clay ratios probably reflects efficient binding of OC released by microbial decomposition of particulate OM after land-use change, including the higher availability of particulate OM by aggregate disruption^12,56^. It seems that reactive mineral surfaces become available through mechanical soil cultivation (e.g. aggregate break up by hand-hoe tillage)^57^ and contribute to OM persistence in this specific mineralogical combination. In fact, land-use-induced losses of OC from light fractions were associated with significant increases in MAOC stocks for the ‛low clay‒high Fe’ combination (Fig. 4, Table S5). To sum up, MAOC represented the quantitatively most relevant carbon fraction in topsoils of almost all soils across the mineralogical combinations (Table S5). While a land-use-induced MAOC loss was observed in most combinations, conjunction of high pedogenic Fe oxides and low aluminous clay contents was associated with a net OC accumulation in the topsoil upon forest-cropland conversion.

### Chemical and biological resistance of bulk and mineral-associated carbon

**Figure 5:**
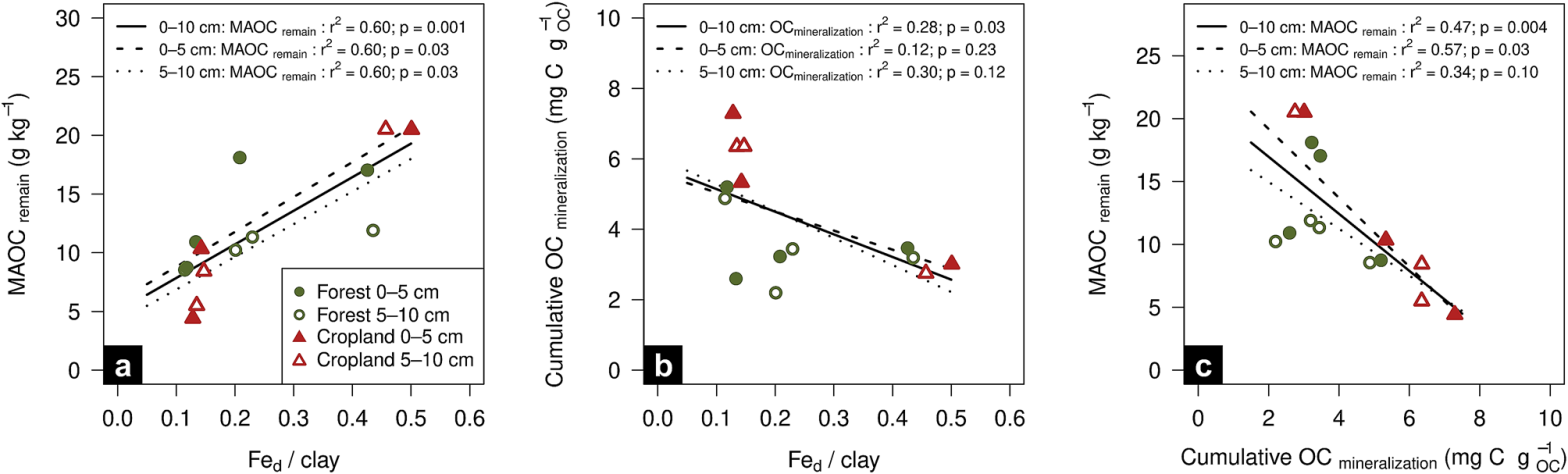
(**a**) MAOC content resistant to chemical oxidation with 6% NaOCl (MAOC_remain_) in relation to the Fe_d_ to aluminous clay ratio. (**b**) Cumulative OC mineralization after 50 days of incubation in relation to the Fe_d_ to aluminous clay ratio. (**c**) MAOC_remain_ content related to the cumulative OC mineralization. Aluminous clay represents the weight sum of kaolinite and gibbsite present in the < 2-µm fraction and Fe_d_ denotes the content of total pedogenic Fe extractable by dithionite-citrate-bicarbonate. All regression models are provided in Table S6.

To further characterize the stability of OM across the mineralogical combinations, we analyzed (i) the chemical resistance of MAOC to 6% NaOCl, and (ii) the biological stability of bulk OC during a 50-day incubation experiment (Table S7, Fig. 5a). Between 41 and 64% of MAOC was removed by the NaOCl treatment, with resistant MAOC comprising between 4.4 and 20.5 g kg^‒1^. The oxidation efficiency was at the upper end compared to artificial goethite-OM associations, where oxidation efficiencies varied between 10 and 45% for low and high OC loadings, respectively^58^. The oxidation-resistant fraction of MAOC was positively related to the Fe_d_ content and slightly negatively to aluminous clay content (Table S6). The opposing effect of Fe_d_ and aluminous clay might be attributed to differences in their overall capacity to protect OC against chemical oxidation, with a higher capacity for goethite than for clay minerals such as vermiculite^59^. Consequently, combinations with high pedogenic Fe to aluminous clay ratios are consistent with higher MAOC resistance to wet chemical oxidation (Fig. 5a).

Our laboratory incubation yielded similar trends in bulk OC resistance to microbial decomposition as the MAOC exhibited toward chemical oxidation treatment. Cumulative microbial OC mineralization during incubation generally decreased with increasing pedogenic Fe to aluminous clay ratio. This trend was significant for the combined soil depth of 0‒10 cm (Fig. 5b). Mineralization losses ranged from 2.6‒7.3 mg CO_2_–C g OC^‒1^ and 2.2‒6.4 mg CO_2_–C g OC^‒1^ in the 0‒5 and 5‒10 cm depth increments, respectively (Table S9). There was a significant inverse relationship between the cumulative microbial OC mineralization and MAOC resistant to chemical oxidation (Fig. 5c), suggesting that mineralogical combinations holding more biologically stable OC contains a larger proportion of MAOC resistant to chemical oxidation. The low aluminous clay and high pedogenic Fe combinations showed in most cases significantly lower bulk OC mineralization compared to other mineralogical combinations under both land uses (Table S9). In contrast, the ‛high clay‒low Fe’ combination under forest exhibited the highest OC mineralization of all treatments. These results in combination with the chemical oxidation data clearly demonstrate that contents in pedogenic Fe oxides are more decisive for OC stabilization in the tropical soils than contents in aluminous clays.

### Implications

**Figure 6:**
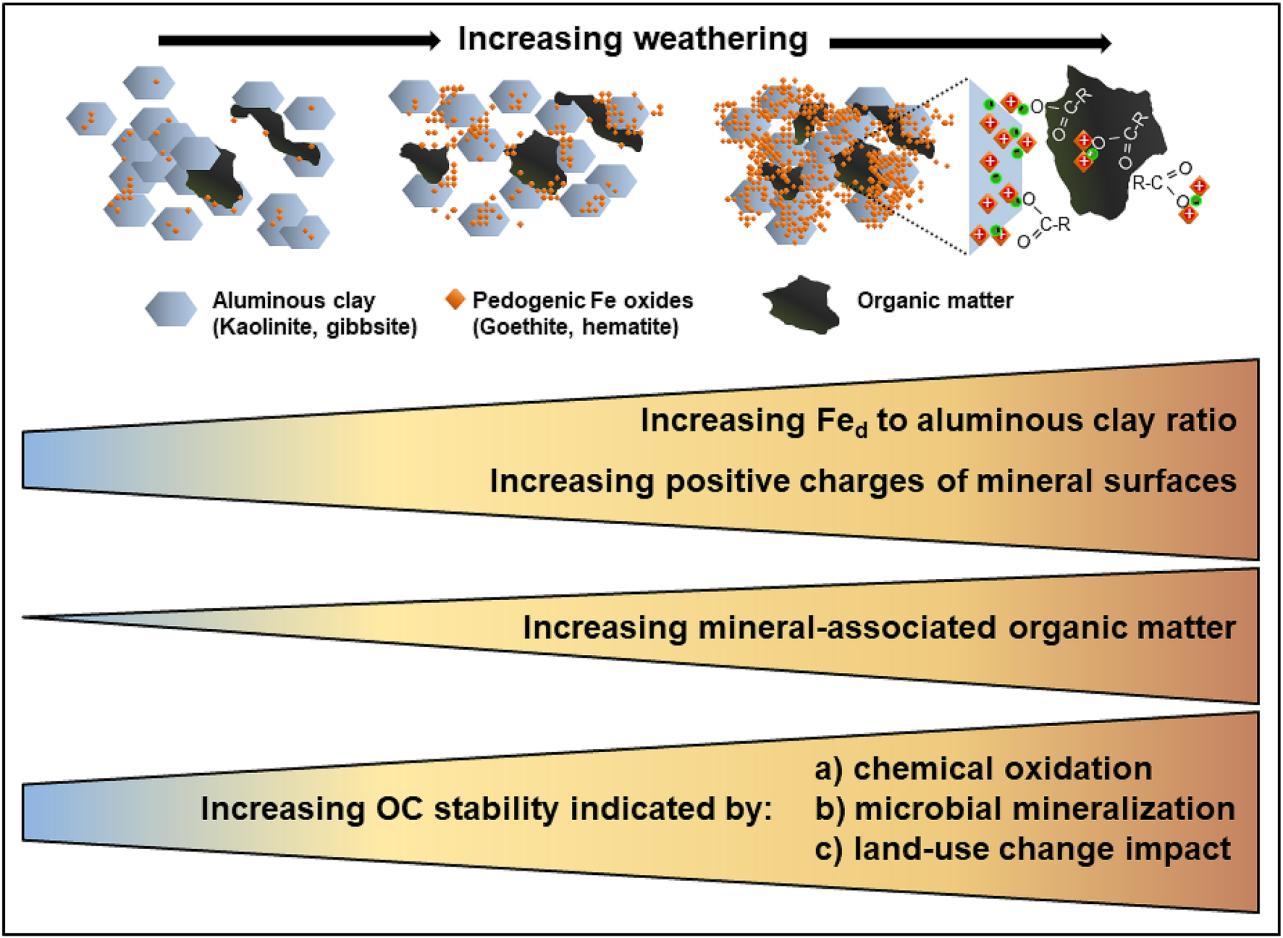
Implications of intensive tropical weathering for the formation of mineral-associated OM and overall OM stability.

Sustainable use of weathered tropical soils strongly relies on OM contents for complying key soil functions such as nutrient supply and water retention. Our data reconfirm that mineralogical soil properties are crucial determinants of OM storage in tropical soils (Fig. 6). A higher OM persistency emerges in soils comprising a high ratio of pedogenic Fe to aluminous clay, rendering them more resilient against disturbances caused by land-use change. We found that stability indicators determined in the laboratory are in good agreement with the persistence of bulk OC and MAOC during land-use change under field conditions. While previous studies often showed land-use changes from forests to cropland drive substantial OC losses^4^, we provide the first evidence that this can be alleviated under certain mineralogical conditions, particularly under conditions with high pedogenic Fe to aluminous clay ratios (0.44‒0.56). Therefore, we suggest the ratio of pedogenic Fe to aluminous clay may be a useful metric for predicting soil OM abundance and persistence in weathered tropical soils.

## Methods

### Study area and soil sampling

The study area is located in the Eastern Usambara Mountains of Northeast Tanzania in close vicinity of the village Amani (5°06ʹ 00ʺ S; 38°38ʹ 00ʺ E). The climate is characterized as humid monsoonal, with a mean annual precipitation of 1918 mm, and a mean annual temperature of 20.6 °C^33^. Soil sampling took place in February 2018 at the end of the long dry season. The small distances between individual sampling sites (maximal 5 km) ensured very similar parent material and climatic conditions^17,32,33^. Six sites under forest and three sites under annual cropping were sampled. On croplands, hand-hoe tillage has been practiced for at least 15 years, typically corn and cassava are grown without the application of chemical fertilizers^32,60^. Processing residues that arise during the preparation of the seedbed are burned before the crops are planted^32^. The average slope angle measured at the study sites was 24 ± 6°. Around a central soil pit at mid-slope position used for soil description, three randomly distributed soil mini-pits were sampled at 0‒5 and 5‒10 cm depth. Additionally, undisturbed soil cores (100 cm^3^) were collected from the central and mini-pits for bulk density determination.

### Basic soil properties

Soils were air-dried and sieved to < 2 mm. Bulk density was determined after drying the soil at 105 °C, and correcting the soil mass for coarse fragments^61^. Potentiometric measurement of pH was conducted by a glass electrode four hours after equilibrating 10 g bulk soil in 25 mL 0.01 M CaCl_2_. The contents of OC and total N were determined by high temperature combustion at 950 °C and thermo-conductivity detection (Vario EL III/Elementar, Heraeus, Langenselbold, Germany). Organic carbon stocks were calculated according to Eq. (1),

1 $$O{C_{stock}} = OC \times BD \times D \times \, \left( {1 - CF/V} \right),$$

where *OC* resembles the OC content (kg kg^‒1^), *BD* the bulk density (kg m^‒3^), *D* the soil depth (m), *CF* the volume of coarse fragments > 2 mm (m^3^), and *V* the volume of sampled soil (m^3^).

### Density fractionation

Light and heavy fractions were obtained by exposing bulk soil (< 2 mm) to a sodium polytungsten solution (Na_6_[H_2_W_12_O_6_], grade = SPT-0; TC-Tungsten Compounds, D-96271) adjusted to 1.6 g cm^–3^ ^52^. The floating free light fraction (fLF) was collected prior to sonication (600 J mL^‒1^), which released occluded light fraction (oLF) material. The remaining soil was considered as heavy fraction (HF) and includes OM associated with minerals. Each fraction was washed with deionized water until the electric conductivity was < 50 µS cm^‒1^ and subsequently freeze-dried. The mean mass and OC recovery were 94 ± 2% and 92 ± 8%, respectively. Organic C and total N contents of fractions were determined by high temperature combustion.

### Aluminous clay and pedogenic Fe oxide contents

A combined Fe extraction and texture analysis was applied to each bulk soil (< 2 mm). For that purpose, five to six grams of bulk soil, pre-treated with 30% H_2_O_2_ to remove OM, were extracted with 30 g sodium dithionite (Na_2_S_2_O_4_) and 1.35 L buffer solution (0.27 M trisodium citrate dihydrate (C_6_H_5_Na_3_O_7_ • 2H_2_O) + 0.11 M sodium bicarbonate (NaHCO_3_)) at 75 °C in a water bath for 15 min^62^. Soil suspensions were centrifuged at 3000 *g* for 15 min and the supernatant decanted. The remaining soil was washed three times with 0.05 L buffer solution, centrifuged, and each solution was combined in a 2-L flask. Concentrations of Fe in extracts were measured by inductively coupled plasma optical emission spectroscopy (ICP-OES) using a CIROS-CCD instrument (Spectro, Kleve, Germany). Dithionite-citrate-bicarbonate-extractable Fe (Fe_d_) represents total pedogenic Fe as released from goethite, hematite, SRO Fe minerals as well as Fe-organic complexes. In addition, ammonium-oxalate-extractable Fe (Fe_o_) was measured on parallel samples^63^. The dithionite-citrate-bicarbonate treated soil was washed with distilled water until the electric conductivity of the supernatant was < 50 µS cm^‒1^. Subsequently, soil texture analysis was performed using the pipette method^64^, providing the clay fraction as measure for the content of aluminous clay (kaolinite, gibbsite). Thresholds differentiating between high and low mineral abundance along the mineralogical combinations were set to 250 g kg^‒1^ for aluminous clay and 60 g kg^‒1^ for pedogenic Fe.

### Total element contents and weathering indicators

Bulk soil (< 2 mm) was analyzed using a NITON™ XL3t-950 He GOLDD + X-ray fluorescence analyzer (Thermo Fisher Scientific) under helium atmosphere in a 2.5-cm Ø sample cup with 6.0-µm polypropylene foil. Filter settings were set for the main (Fe, Zr, Sr, Rb, and heavy metals) and low range (Ti, Ca, K) to 20 s, and for the light range (Al, Si, P, Mg) to 50 s. Reference samples used were USGS GSP-2, GSJ JSL1, NCS DC 71,311, SACCRM SARM46, GSJ-JH1, and LNS CGL006. Weathering indicators, *K*_*r*_ and *K*_*i*_, were calculated according to Eqs. (2 and 3)^65^.

2 $${K_r} = \frac{SiO_{2}}{\left( {{Al_2}{O_3} + {Fe_2}{O_3}} \right)}$$

3 $${K}_{i}=\frac{{SiO}_{2}}{{Al}_{2}{O}_{3}}$$

### X-ray diffraction (XRD)

The mineral composition of the clay fraction obtained by the combined Fe extraction and texture analysis was analyzed by X-ray diffraction (X’Pert Pro-MPD; PANanalytical, Almelo, The Netherlands). Oriented clay specimens saturated with K^+^ (with and without heating to 550 °C) and Mg^2+^ (with and without ethylene glycol) were measured in the range of 3‒30° 2ϴ with CuKα radiation (45 kV, 40 mA), and a step size of 0.01°. Phase identification was performed using the MATCH! software (version 3.10.0.176) and the Crystallographic Open Database (COD-Inorg REV218120 2019.09.10).

### Mössbauer spectroscopy

^57^Fe-Mössbauer analysis was performed on composite heavy soil fractions from all mineralogical combinations. Composite samples were prepared using samples from each mini-pit assigned to a particular mineralogical combination, depth, and land use based on equal weights. Samples were mounted between layers of Kapton tape and ^57^Fe Mössbauer spectra were recorded in transmission mode with a variable-temperature He-cooled cryostat (Janis Research Co.) and a 1024 channel detector. A ^57^Co source (50 mCi) embedded in a Rh matrix was used at room temperature. The velocity (i.e., γ-ray energy) was calibrated using α-Fe foil at 295 K, and all center shifts (CSs) and peak positions are reported with respect to this standard. Detailed information regarding the Mössbauer spectra modeling is provided in the Supplementary Material (Sect. 2). The fitted Mössbauer parameters are summarized in Table S3, and further details are given in Figure S2.

### Specific surface area (SSA)

The SSA was analyzed on composite heavy soil fractions for each mineralogical combination. Organic matter was removed from the samples by treatment with 6% NaOCl^31^. The SSA of freeze-dried and homogenized samples was analyzed in duplicates based on a 10-point adsorption of N_2_ at 77 K in the relative pressure range of 0.05–0.30^66^ with an Autosorb IQ2 instrument (Quantachrome Instruments, Boynton Beach, FL 33,426). Before measurement, samples were degassed at 60 °C until the pressure increase in the analysis cell was < 20 mTorr min^–1^. The SSA was corrected for OM remaining after NaOCl treatment^67^.

### ^13^C nuclear magnetic resonance spectroscopy

The chemical composition of litter samples was analyzed by ^13^C cross-polarization magic angle spinning nuclear magnetic resonance (^13^C CPMAS NMR) spectroscopy (Bruker DSX 200, Billerica, USA). We used a 7-mm zirconium dioxide rotor for the measurements, recorded the spectra at 6.8 kHz, and set the acquisition time to 0.01024 s. To avoid Hartmann-Hahn mismatches, a ramped ^1^H pulse was implemented during a 1 ms contact time. Tetramethylsilane served as chemical shift reference (0 ppm). Spectra were integrated according to Wilson et al.^68^ and Beudert et al.^69^ with minor adjustments according to Mueller and Koegel–Knabner^70^ as follows: -10‒45 ppm (alkyl C), 45‒110 ppm (O/N alkyl C), 110‒160 ppm (aromatic C), and 160‒220 ppm (carboxyl C).

### Wet chemical oxidation and soil respiration

The resistance of MAOC against chemical oxidation was determined in duplicates using the same composite heavy fraction materials as described above. We placed 1.00 *g* of dried (40 °C) material in centrifuge tubes and added 50 mL of 6% NaOCl (pH 8, solid/solution ratio of 1:50). After 6 h at 22 °C the suspensions were centrifuged at 6000 *g* for 30 min^31^. The supernatant was replaced by fresh NaOCl and the procedure was repeated in total five times. The material was washed with deionized water until the electric conductivity of the supernatant was < 50 µS cm^‒1^, freeze-dried, and the remaining MAOC content determined by high temperature combustion.

The biological stability of bulk OC was determined by an aerobic incubation of 20 g sieved (< 2 mm) and rewetted soil. The water potential of each sample was adjusted to pF 2.0 (close to field capacity), which was maintained regularly by weighing. Soils, including three blank samples, were incubated in glass bottles at 22 °C for 50 days in the dark. Soil OC mineralization was quantified by trapping evolved CO_2_-C in 0.1 M NaOH, subsequent precipitation with BaCl_2_, and titration against 0.1 M HCl. Phenolphthalein was used as the indicator in the titration reaction. The captured CO_2_-C was measured in a 4-day interval for the first 16 days, followed by a 7-day interval; soil-derived CO_2_ emissions were corrected for blank measurements.

### Statistics and calculations

Means and standard deviations of data were calculated with the software package R (version 3.6.0). To test for significant differences between mineralogical combinations, linear model function [lm()] was used in combination with analysis of variance [aov(lm()]. Tukey-HSD test was used as a post-hoc comparison of means; the LSD-test was applied in the case of non-equality of variances. Linear regression and correlation analysis were used to test for relations between independent variables. Statistically significant differences are reported at a significance level of p < 0.05.

## Supplementary Information

Supplementary Information.

## Data Availability

The datasets generated during and/or analyzed during the current study are available from the corresponding author on reasonable request.
