# Author Correction: Iron oxides and aluminous clays selectively control soil carbon storage and stability in the humid tropics

**DOI:** 10.1038/s41598-021-00215-8

**Published:** 2021-10-12

**Authors:** Maximilian Kirsten, Robert Mikutta, Cordula Vogel, Aaron Thompson, Carsten W. Mueller, Didas N. Kimaro, Huig L. T. Bergsma, Karl-Heinz Feger, Karsten Kalbitz

**Affiliations:** 1 Technische Universität Dresden, Institute of Soil Science and Site Ecology, Tharandt, Germany; 2 Soil Science and Soil Protection, Martin Luther University Halle-Wittenberg, Halle (Saale), Germany; 3 Department of Crop and Soil Sciences, University of Georgia, Athens, GA USA; 4 Department of Geosciences and Natural Resource Management, University of Copenhagen, Copenhagen, Denmark; 5 Directorate of Research Innovations and Consultancy, Mwenge Catholic University, Moshi, Tanzania; 6 BodemBergsma, Blikakker 8, 7421 GD Deventer, The Netherlands

Correction to: *Scientific Reports* 10.1038/s41598-021-84777-7, published online 03 March 2021

The original version of this Article contained errors in Figure 3, where the bar showing the mineralogical combination of “high clay-low Fe (0-10 cm)” was incorrectly given.

The original Figure 3 and accompanying legend appear below.

**Figure 3:**
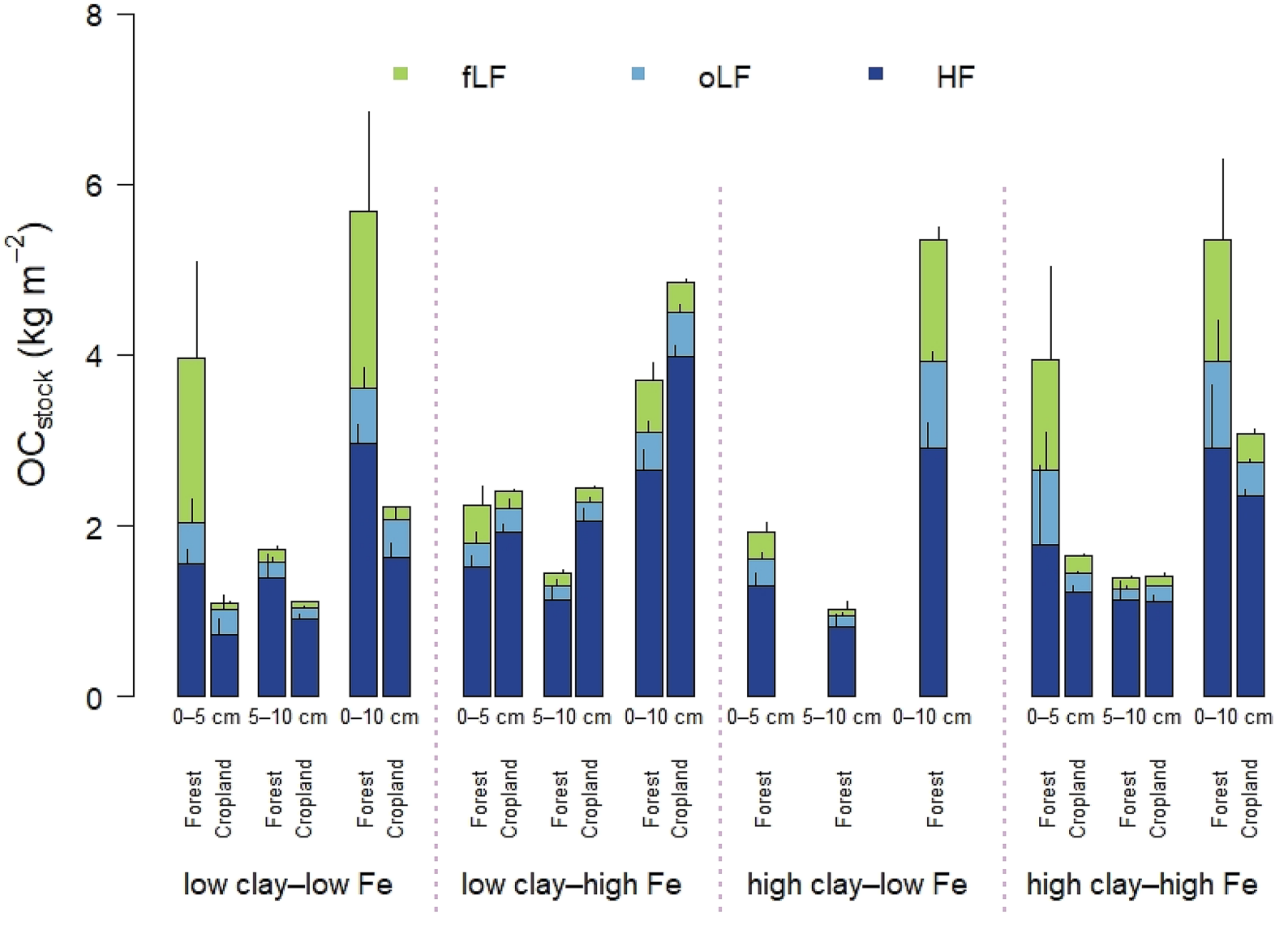
Bulk OC stocks related to soil density fractions (fLF and oLF = free and occluded light fraction; HF = heavy fraction) in mineralogical combinations under forest and cropland separated by soil depth. Sample numbers for the combinations are as follows: ‛low clay‒low Fe’ under forest (*n* = 4), ‛low clay‒high Fe’ under forest (*n* = 4), ‛high clay‒low Fe’ under forest (*n* = 3), ‛high clay‒high Fe’ under forest (*n* = 7); all cropland combinations (*n* = 3).

The original Article has been corrected.

