# Supplementary material for: Iron oxides and aluminous clays selectively control soil carbon storage and stability in the humid tropics
Source: Sci Rep. 2021 Mar 3;11:5076. doi: 10.1038/s41598-021-84777-7 (PMC7970839; doi:10.1038/s41598-021-84777-7)
Supplement: Supplementary file 1 — Supplementary Information. [file 41598_2021_84777_MOESM1_ESM.docx]

**Supplementary Material**

**Iron oxides and aluminous clays selectively control soil carbon storage and stability in the humid tropics**

Maximilian Kirsten^1^, Robert Mikutta^2^, Cordula Vogel^1^, Aaron Thompson^3^, Carsten W. Mueller^4^, Didas N. Kimaro^5^, Huig L.T. Bergsma^6^, Karl-Heinz Feger^1^, Karsten Kalbitz^1^

^1^ Technische Universität Dresden, Institute of Soil Science and Site Ecology, Tharandt, Germany

^2^ Martin-Luther-Universität Halle-Wittenberg, Soil Science and Soil Protection, Halle (Saale), Germany

^3^ University of Georgia, Department of Crop and Soil Sciences, Athens, GA, USA

^4^ University of Copenhagen, Department of Geosciences and Natural Resource Management, Copenhagen, Denmark

^5^ Mwenge Catholic University, Innovations and Consultancy, Moshi, Tanzania

^6^ BodemBergsma, Blikakker 8, 7421 GD Deventer, The Netherlands

Table of contents

[Section 1: General soil description 2](#_Toc64280558)

[Section 2: ^57^Fe Mössbauer spectroscopy of 6](#_Toc64280559)

[Section 3: Magnetic susceptibility 12](#_Toc64280560)

[Section 4: Density fractionation 14](#_Toc64280561)

[Section 5: Model parameters 16](#_Toc64280562)

[Section 6: Specific surface area analysis and chemical resistance of MAOC 19](#_Toc64280563)

[Section 7: ^13^C NMR analysis 21](#_Toc64280564)

[Section 8: Microbial respiration 22](#_Toc64280565)

[Section 9: Figures 23](#_Toc64280566)

[Section 9: References 55](#_Toc64280567)

# Section 1: General soil description

**Table S1:** Overview of sites and properties of soils selected for the mineralogical combinations: bulk density (BD), potential cation exchange capacity (CEC_pot_, measured at pH 7), base saturation related to the potential cation exchange capacity (BS), organic carbon contents (OC), total nitrogen contents (N_total_). All soils belong to the reference soil group Acrisols or Alisols^1^ Illluvial clay accumulation was detected in the subsoil, where clay coatings covered > 5% of aggregate surface. All data belong to samples taken from a soil pit located at mid slope position of each site.

| **Site** | **Code** | **Land-use** | **Coordinates** | **Soil depth** |  | **Horizon** |  | **BD** |  | **Sand** | **Silt** | **Clay** |  | **pH** |  | **CEC_pot_** |  | **BS** |  | **OC** | **N_total_** |
| --- | --- | --- | --- | --- | --- | --- | --- | --- | --- | --- | --- | --- | --- | --- | --- | --- | --- | --- | --- | --- | --- |
|  |  |  |  | (cm) |  |  |  | (g cm^‒3^) |  |  | (g kg^‒1^) |  |  | (H_2_O) |  | (cmol_c_ kg^‒1^) |  | (%) |  | (g kg^‒1^) | |
|  |  |  |  |  |  |  |  |  |  |  |  |  |  |  |  |  |  |  |  |  |  |
|  |  |  |  |  |  |  |  |  |  |  |  |  |  |  |  |  |  |  |  |  |  |
| **Turaco Bird Trail** | F1 | Forest | 5°06ˈ26 ̎ S; 38°36ˈ02 ̎ E | 0–5 |  | Ah |  | 1.3 |  | 505 | 118 | 377 |  | 4.1 |  | 17 |  | 11 |  | 4.0 | 0.3 |
|  |  |  |  | 5–30 |  | AB |  | 1.2 |  | 386 | 141 | 473 |  | 4.3 |  | 14 |  | 7 |  | 1.3 | 0.1 |
|  |  |  |  | 30–52 |  | Bt1 |  | 1.3 |  | 387 | 118 | 495 |  | 4.7 |  | 14 |  | 19 |  | 1.0 | 0.1 |
|  |  |  |  | 52–88 |  | Bt2 |  | 1.3 |  | 348 | 131 | 521 |  | 4.9 |  | 13 |  | 18 |  | 0.7 | 0.1 |
|  |  |  |  | 88–100 |  | Bt3 |  | 1.3 |  | 346 | 121 | 532 |  | 4.9 |  | 16 |  | 16 |  | 0.7 | 0.1 |
|  |  |  |  |  |  |  |  |  |  |  |  |  |  |  |  |  |  |  |  |  |  |
| **Derema Forest** | F2 | Forest | 5°03ˈ57 ̎ S; 38°38ˈ38 ̎ E | 0–9 |  | Ah |  | 0.8 |  | 587 | 73 | 340 |  | 4.4 |  | 47 |  | 4 |  | 8.0 | 0.6 |
|  |  |  |  | 9–17 |  | AB |  | 1.2 |  | 522 | 123 | 355 |  | 4.7 |  | 20 |  | 5 |  | 4.4 | 0.4 |
|  |  |  |  | 17–41 |  | Bt1 |  | 1.4 |  | 402 | 150 | 448 |  | 4.4 |  | 11 |  | 11 |  | 1.7 | 0.2 |
|  |  |  |  | 41–71 |  | Bt2 |  | 1.3 |  | 362 | 110 | 528 |  | 4.5 |  | 9 |  | 8 |  | 1.0 | 0.2 |
|  |  |  |  | 71–100 |  | Bt3 |  | 1.3 |  | 362 | 100 | 538 |  | 4.4 |  | 8 |  | 9 |  | 0.9 | 0.1 |
|  |  |  |  |  |  |  |  |  |  |  |  |  |  |  |  |  |  |  |  |  |  |
| **Kihuhi Forest** | F3 | Forest | 5°09ˈ12 ̎ S; 38°37ˈ15 ̎ E | 0–12 |  | Ah |  | 0.9 |  | 437 | 133 | 430 |  | 4.7 |  | 20 |  | 8 |  | 5.0 | 0.3 |
|  |  |  |  | 12–32 |  | AB |  | 0.8 |  | 492 | 70 | 438 |  | 4.7 |  | 10 |  | 7 |  | 1.0 | 0.1 |
|  |  |  |  | 32–60 |  | Bt1 |  | 1.3 |  | 412 | 130 | 458 |  | 4.7 |  | 7 |  | 22 |  | 1.1 | 0.1 |
|  |  |  |  | 60–85 |  | Bt2 |  | 1.2 |  | 442 | 110 | 448 |  | 4.8 |  | 7 |  | 24 |  | 0.8 | 0.1 |
|  |  |  |  | 85–100 |  | Bt3 |  | 1.2 |  | 402 | 140 | 458 |  | 5.1 |  | 12 |  | 10 |  | 0.6 | 0.1 |
|  |  |  |  |  |  |  |  |  |  |  |  |  |  |  |  |  |  |  |  |  |  |
| **Amani East Forest** | F4 | Forest | 5°05ˈ40 ̎ S;38°37ˈ56 ̎ E | 0–2 |  | Ah |  | 1.0 |  | 457 | 173 | 370 |  | 4.5 |  | 48 |  | 7 |  | 11.2 | 0.8 |
|  |  |  |  | 2–17 |  | AB |  | 0.7 |  | 377 | 123 | 500 |  | 3.9 |  | 18 |  | 9 |  | 5.2 | 0.3 |
|  |  |  |  | 17–43 |  | Bt1 |  | 1.2 |  | 312 | 100 | 588 |  | 5.1 |  | 8 |  | 15 |  | 1.4 | 0.1 |
|  |  |  |  | 43–68 |  | Bt2 |  | 1.0 |  | 302 | 100 | 598 |  | 4.7 |  | 5 |  | 68 |  | 0.7 | 0.1 |
|  |  |  |  | 68–100 |  | Bt3 |  | 1.3 |  | 272 | 100 | 628 |  | 4.6 |  | 7 |  | 25 |  | 0.9 | 0.1 |
|  |  |  |  |  |  |  |  |  |  |  |  |  |  |  |  |  |  |  |  |  |  |
| **Kwamkoro Forest** | F5 | Forest | 5°08ˈ42 ̎ S; 38°37ˈ32 ̎ E | 0–15 |  | Ah |  | 0.9 |  | 639 | 127 | 234 |  | 3.9 |  | 17 |  | 4 |  | 5.1 | 0.4 |
|  |  |  |  | 15–25 |  | AB |  | 1.1 |  | 557 | 170 | 273 |  | 4.4 |  | 11 |  | 3 |  | 2.3 | 0.2 |
|  |  |  |  | 25–40 |  | Bt1 |  | 1.2 |  | 513 | 187 | 300 |  | 4.6 |  | 11 |  | 3 |  | 1.8 | 0.1 |
|  |  |  |  | 40–60 |  | Bt2 |  | 1.1 |  | 503 | 179 | 318 |  | 4.7 |  | 12 |  | 2 |  | 1.5 | 0.1 |
|  |  |  |  | 60–100 |  | Bt3 |  | 1.1 |  | 512 | 156 | 331 |  | 4.8 |  | 11 |  | 1 |  | 1.3 | 0.1 |
|  |  |  |  |  |  |  |  |  |  |  |  |  |  |  |  |  |  |  |  |  |  |
| **Monga Forest** | F6 | Forest | 5°05ˈ18 ̎ S; 38°36ˈ28 ̎ E | 0–10 |  | Ah |  | 0.9 |  | 359 | 179 | 463 |  | 4.5 |  | 28 |  | 33 |  | 7.9 | 0.7 |
|  |  |  |  | 10–20 |  | AB |  | 1.1 |  | 265 | 196 | 539 |  | 4.4 |  | 19 |  | 6 |  | 2.8 | 0.3 |
|  |  |  |  | 20–40 |  | Bt1 |  | 1.1 |  | 264 | 180 | 557 |  | 4.7 |  | 17 |  | 7 |  | 1.7 | 0.2 |
|  |  |  |  | 40–60 |  | Bt2 |  | 1.0 |  | 281 | 172 | 547 |  | 4.7 |  | 21 |  | 5 |  | 1.2 | 0.1 |
|  |  |  |  | 60–100 |  | Bt3 |  | 1.0 |  | 277 | 163 | 560 |  | 4.7 |  | 19 |  | 4 |  | 1.0 | 0.1 |
|  |  |  |  |  |  |  |  |  |  |  |  |  |  |  |  |  |  |  |  |  |  |
| **Maramba Cropland** | A1 | Cropland | 5°03ˈ57 ̎ S; 38°36ˈ59 ̎ E | 0–25 |  | Ah |  | 1.2 |  | 632 | 63 | 305 |  | 5.8 |  | 9 |  | 50 |  | 1.9 | 0.2 |
|  |  |  |  | 25–60 |  | Bt1 |  | 1.4 |  | 442 | 53 | 505 |  | 5.0 |  | 7 |  | 39 |  | 1.4 | 0.1 |
|  |  |  |  | 60–90 |  | Bt2 |  | 1.4 |  | 402 | 63 | 535 |  | 4.9 |  | 5 |  | 46 |  | 1.1 | 0.1 |
|  |  |  |  | 90–100 |  | Bt3 |  | 1.4 |  | 382 | 63 | 555 |  | 5.0 |  | 6 |  | 25 |  | 0.5 | 0.1 |
|  |  |  |  |  |  |  |  |  |  |  |  |  |  |  |  |  |  |  |  |  |  |
| **Kwemwewe Cropland** | A3 | Cropland | 5°03ˈ51 ̎ S; 38°35ˈ25 ̎ E | 0–8 |  | Ah |  | 1.0 |  | 452 | 143 | 405 |  | 6.1 |  | 8 |  | 44 |  | 3.4 | 0.3 |
|  |  |  |  | 8–23 |  | AB |  | 1.2 |  | 352 | 113 | 535 |  | 5.2 |  | 13 |  | 23 |  | 1.6 | 0.1 |
|  |  |  |  | 23–50 |  | Bt1 |  | 1.2 |  | 242 | 103 | 655 |  | 4.9 |  | 7 |  | 31 |  | 0.9 | 0.1 |
|  |  |  |  | 50–80 |  | Bt2 |  | 1.2 |  | 262 | 93 | 645 |  | 4.8 |  | 7 |  | 34 |  | 0.5 | 0.1 |
|  |  |  |  | 80–00 |  | Bt3 |  | 1.2 |  | 252 | 93 | 655 |  | 4.8 |  | 6 |  | 40 |  | 0.7 | 0.1 |
|  |  |  |  |  |  |  |  |  |  |  |  |  |  |  |  |  |  |  |  |  |  |
| **Mbomole Cropland** | A4 | Cropland | 5°05ˈ17 ̎ S; 38°37ˈ04 ̎ E | 0–12 |  | Ah |  | 1.0 |  | 398 | 197 | 405 |  | 5.1 |  | 17 |  | 23 |  | 3.0 | 0.2 |
|  |  |  |  | 12–23 |  | AB |  | 1.0 |  | 355 | 212 | 433 |  | 5.2 |  | 15 |  | 19 |  | 2.1 | 0.2 |
|  |  |  |  | 23–46 |  | Bt1 |  | 1.2 |  | 316 | 210 | 474 |  | 4.9 |  | 16 |  | 14 |  | 1.4 | 0.1 |
|  |  |  |  | 46–72 |  | Bt2 |  | 1.0 |  | 330 | 210 | 460 |  | 5.0 |  | 15 |  | 14 |  | 1.1 | 0.1 |
|  |  |  |  | 72–100 |  | Bt3 |  | 0.9 |  | 354 | 169 | 477 |  | 4.9 |  | 16 |  | 8 |  | 1.1 | 0.1 |
|  |  |  |  |  |  |  |  |  |  |  |  |  |  |  |  |  |  |  |  |  |  |

**Table S2:** Basic soil data for the two sampled soil depths along the mineralogical combinations: aluminous clay (hydrogen peroxide and dithionite-citrate-bicarbonate-treated clay), dithionite-citrate-bicarbonate-extractable Fe (Fe_d_), bulk density (BD), effective cation exchange capacity (CEC_eff_), base saturation related to CEC_eff_ (BS), 30% H_2_O_2_ oxidized and dithionite-citrate-bicarbonate-treated sand and silt contents, total iron, aluminum, and silica contents (Fe_XRF_, Al_XRF_, and Si_XRF_) measured with X-ray florescence spectroscopy (XRF), oxalate-extractable Fe and Al content (Fe_o_ and Al_o_), weathering indicators (*K*_r_ and *K*_i_) based on Becquer et al.^2^. Aluminous clay represents the weight sum of kaolinite and gibbsite present in the < 2-µm fraction after removal of OM and pedogenic Fe oxides. Lower case letters indicate significant differences within a certain land-use as separated by depth. Sample numbers for the combinations are as follows: ‛low clay‒low Fe’ under forest (*n* = 4), ‛low clay‒high Fe’ under forest (*n* = 4), ‛high clay‒low Fe’ under forest (*n* = 3), ‛high clay‒high Fe’ under forest (*n* = 7); all cropland combinations (*n* = 3).

| **Land-use** | **Mineralogical Combination** | **Depth** |  |  | **Clay** | **Fe_d_** |  | **BD** |  | **pH** |  | **CEC_eff_** |  | **BS** |  | **Sand** | **Silt** |  | **Fe_XRF_** | **Al_XRF_** | **Si_XRF_** |  | **Fe_o_** | **Al_o_** |  | ***K*_r_** | ***K*_i_** |
| --- | --- | --- | --- | --- | --- | --- | --- | --- | --- | --- | --- | --- | --- | --- | --- | --- | --- | --- | --- | --- | --- | --- | --- | --- | --- | --- | --- |
|  |  | (cm) |  |  | (g kg^‒1^) | |  | (g cm^‒3^) |  | (0.01 M CaCl_2_) |  | (cmol_c_ kg^‒1^) |  | (%) |  | (g kg^‒1^) | | | | | | | | |  |  |  |
|  |  |  |  |  |  |  |  |  |  |  |  |  |  |  |  |  |  |  |  |  |  |  |  |  |  |  |  |
|  |  |  |  |  |  |  |  |  |  |  |  |  |  |  |  |  |  |  |  |  |  |  |  |  |  |  |  |
| **Forest** | **Low** aluminous clay– | 0‒5 |  |  | **149^b^**  (19) | **21^d^**  (4) |  | **1.1^a^**  (0.1) |  | **3.5^b^**  (0.1) |  | **5.7^a^**  (2.6) |  | **41^b^**  (8) |  | **788^a^**  (21) | **63^c^**  (24) |  | **44^c^**  (5) | **78^c^**  (12) | **198^a^**  (15) |  | **1.4^a^**  (0.3) | **1.2^a^**  (0.2) |  | **2.1^a^**  (0.5) | **3.0^a^**  (0.7) |
|  | **Low** pedogenic Fe oxides | 5‒10 |  |  | **181^b^**  (19) | **38^b^**  (13) |  | **1.1^a^**  (0.1) |  | **3.7^b^**  (0.1) |  | **2.9^a^**  (0.1) |  | **8^a^**  (2) |  | **712^a^**  (46) | **107^b^**  (57) |  | **59^b^**  (1) | **108^b^**  (10) | **169^b^**  (9) |  | **1.8^a^**  (0.3) | **1.4^a^**  (0.2) |  | **1.2^a^**  (0.3) | **1.7^a^**  (0.3) |
|  |  |  |  |  |  |  |  |  |  |  |  |  |  |  |  |  |  |  |  |  |  |  |  |  |  |  |  |
| **Forest** | **Low** aluminous clay– | 0‒5 |  |  | **182^b^**  (38) | **78^a^**  (14) |  | **0.9^b^**  (0.1) |  | **3.8^a^**  (0.2) |  | **5.6^a^**  (1.7) |  | **50^b^**  (14) |  | **617^b^**  (36) | **201^a^**  (52) |  | **109^a^**  (14) | **111^a^**  (9) | **128^b^**  (14) |  | **1.3^a^**  (0.2) | **1.5^a^**  (0.2) |  | **0.8^c^**  (0.2) | **1.3^c^**  (0.3) |
|  | **High** pedogenic Fe oxides | 5‒10 |  |  | **174^b^**  (42) | **77^a^**  (4) |  | **0.9^b^**  (0.1) |  | **3.8^ab^**  (0.1) |  | **3.2^a^**  (0.9) |  | **26^a^**  (15) |  | **647^b^**  (49) | **179^a^**  (26) |  | **112^a^**  (9) | **119^a^**  (4) | **130^c^**  (14) |  | **1.3^b^**  (0.1) | **1.6^a^**  (0.3) |  | **0.7^b^**  (0.1) | **1.2^b^**  (0.1) |
|  |  |  |  |  |  |  |  |  |  |  |  |  |  |  |  |  |  |  |  |  |  |  |  |  |  |  |  |
| **Forest** | **High** aluminous clay– | 0‒5 |  |  | **298^a^**  (41) | **36^c^**  (5) |  | **0.9^ab^**  (0.0) |  | **4.0^a^**  (0.2) |  | **5.2^a^**  (1.1) |  | **62^ab^**  (14) |  | **571^c^**  (19) | **131^b^**  (32) |  | **59^c^**  (1) | **112^a^**  (4) | **191^a^**  (3) |  | **0.9^b^**  (0.0) | **1.3^a^**  (0.2) |  | **1.4^b^**  (0.1) | **1.9^b^**  (0.1) |
|  | **Low** pedogenic Fe oxides | 5‒10 |  |  | **374^a^**  (24) | **44^b^**  (7) |  | **0.9^ab^**  (0.0) |  | **3.9^ab^**  (0.1) |  | **3.0^a^**  (0.4) |  | **27^a^**  (11) |  | **489^c^**  (24) | **137^ab^**  (1) |  | **66^b^**  (4) | **125^a^**  (7) | **186^a^**  (19) |  | **1.0^b^**  (0.1) | **1.5^a^**  (0.3) |  | **1.2^a^**  (0.2) | **1.7^a^**  (0.3) |
|  |  |  |  |  |  |  |  |  |  |  |  |  |  |  |  |  |  |  |  |  |  |  |  |  |  |  |  |
| **Forest** | **High** aluminous clay– | 0‒5 |  |  | **318^a^**  (41) | **67^b^**  (5) |  | **0.9^b^**  (0.1) |  | **4.1^a^**  (0.2) |  | **7.8^a^**  (1.8) |  | **72^a^**  (15) |  | **530^c^**  (28) | **152^b^**  (24) |  | **96^b^**  (6) | **97^b^**  (10) | **146^b^**  (15) |  | **1.2^ab^**  (0.3) | **1.9^a^**  (0.8) |  | **1.0^c^**  (0.2) | **1.7^bc^**  (0.3) |
|  | **High** pedogenic Fe oxides | 5‒10 |  |  | **349^a^**  (40) | **81^a^**  (6) |  | **0.9^b^**  (0.1) |  | **4.0^a^**  (0.1) |  | **4.9^a^**  (4.0) |  | **40^a^**  (25) |  | **473^c^**  (35) | **178^a^**  (45) |  | **114^a^**  (5) | **123^a^**  (4) | **147^bc^**  (11) |  | **1.3^b^**  (0.1) | **1.7^a^**  (0.2) |  | **0.8^b^**  (0.1) | **1.4^b^**  (0.1) |
|  |  |  |  |  |  |  |  |  |  |  |  |  |  |  |  |  |  |  |  |  |  |  |  |  |  |  |  |
| **Cropland** | **Low** aluminous clay‒ | 0‒5 |  |  | **227^b^**  (6) | **30^c^**  (2) |  | **1.2^a^**  (0.0) |  | **5.0^b^**  (0.1) |  | **5.1^b^**  (0.2) |  | **97^ab^**  (1) |  | **670^a^**  (8) | **103^c^**  (4) |  | **43^c^**  (2) | **96^b^**  (4) | **243^a^**  (2) |  | **0.6^c^**  (0.0) | **1.1^c^**  (0.1) |  | **2.2^a^**  (0.1) | **2.9^a^**  (0.1) |
|  | **Low** pedogenic Fe oxides | 5‒10 |  |  | **213^b^**  (24) | **29^c^**  (4) |  | **1.2^a^**  (0.0) |  | **5.0^ab^**  (0.1) |  | **5.1^b^**  (0.2) |  | **98^a^**  (0) |  | **669^a^**  (8) | **118^b^**  (28) |  | **43^c^**  (2) | **97^b^**  (5) | **238^a^**  (8) |  | **0.6^c^**  (0.0) | **1.1^b^**  (0.1) |  | **2.1^a^**  (0.2) | **2.8^a^**  (0.2) |
|  |  |  |  |  |  |  |  |  |  |  |  |  |  |  |  |  |  |  |  |  |  |  |  |  |  |  |  |
| **Cropland** | **Low** aluminous clay– | 0‒5 |  |  | **198^b^**  (29) | **101^a^**  (4) |  | **1.0^b^**  (0.0) |  | **4.9^c^**  (0.1) |  | **5.1^b^**  (0.2) |  | **96^b^**  (2) |  | **602^b^**  (17) | **200^a^**  (13) |  | **133^a^**  (1) | **116^a^**  (1) | **127^c^**  (2) |  | **1.5^a^**  (0.0) | **4.1^a^**  (0.2) |  | **0.7^c^**  (0.0) | **1.3^c^**  (0.0) |
|  | **High** pedogenic Fe oxides | 5‒10 |  |  | **215^b^**  (23) | **100^a^**  (5) |  | **1.0^b^**  (0.0) |  | **4.8^b^**  (0.1) |  | **5.0^b^**  (1.2) |  | **93^a^**  (6) |  | **579^b^**  (19) | **206^a^**  (4) |  | **137^a^**  (3) | **116^a^**  (3) | **125^c^**  (5) |  | **1.7^a^**  (0.1) | **4.3^a^**  (0.6) |  | **0.6^c^**  (0.0) | **1.2^c^**  (0.1) |
|  |  |  |  |  |  |  |  |  |  |  |  |  |  |  |  |  |  |  |  |  |  |  |  |  |  |  |  |
| **Cropland** | **High** aluminous clay– | 0‒5 |  |  | **434^a^**  (18) | **63^b^**  (3) |  | **1.0^b^**  (0.1) |  | **5.4^a^**  (0.0) |  | **9.4^a^**  (0.5) |  | **98^a^**  (1) |  | **437^c^**  (14) | **129^b^**  (12) |  | **83^b^**  (1) | **120^a^**  (4) | **178^b^**  (1) |  | **1.2^b^**  (0.0) | **1.4^b^**  (0.0) |  | **1.1^b^**  (0.0) | **1.7^b^**  (0.1) |
|  | **High** pedogenic Fe oxides | 5‒10 |  |  | **438^a^**  (17) | **66^b^**  (4) |  | **1.0^b^**  (0.1) |  | **5.2^a^**  (0.1) |  | **7.3^a^**  (0.7) |  | **98^a^**  (1) |  | **399^c^**  (18) | **163^ab^**  (35) |  | **85^b^**  (2) | **122^a^**  (3) | **174^b^**  (3) |  | **1.2^b^**  (0.1) | **1.3^b^**  (0.2) |  | **1.1^b^**  (0.0) | **1.6^b^**  (0.0) |
|  |  |  |  |  |  |  |  |  |  |  |  |  |  |  |  |  |  |  |  |  |  |  |  |  |  |  |  |

# Section 2: ^57^Fe Mössbauer spectroscopy of

^57^Fe Mössbauer (MB) spectroscopy was employed to evaluate the Fe site populations in 14 composite samples across the mineralogical combinations. Freeze-dried samples of the composite heavy fraction material derived from a density separation were used.

*MB collection and analysis methods*

Transmission ^57^Fe MB spectroscopy was performed with a variable temperature He-cooled system with a 1024 channel detector. A ^57^Co source (~50 mCi or less) embedded in a Rh matrix was used at room temperature. Freeze-dried powder samples were mounted between two pieces of 0.127 mm thickness Kapton tape and transferred to the spectrometer cryostat. Velocity (i.e., gamma-ray energy) was calibrated using α-Fe foil at 295 K and all center shifts (CSs) and peak positions are reported with respect to this standard. The transducer was operated in constant acceleration mode and folding was performed to achieve a flat background.

*MB spectral analysis approach*

Mössbauer spectral fitting of all spectra was performed using the Voigt-based fitting (VBF) method of Rancourt and Ping^3^ for quadrupole splitting distributions (QSDs) and combined hyperfine field distributions (HFDs), as implemented in the Recoil^TM^ software, ISA Inc. All VBF MB parameter definitions and a description of the relevant notation are given by Rancourt and Ping^3^. All errors in MB fitting parameters are two-standard deviation (2σ) errors, as calculated by Recoil^TM^. In reporting quantitative phase abundances or site populations it is assumed that the MB recoilless fractions of all detected phases or Fe-bearing components are equal, such that subspectral areas (expressed as fractions of total spectral area) are equal to the amounts of Fe (expressed as fractions of total Fe) in the corresponding phases or components. This assumption is expected to be valid at cryogenic temperatures, and also to be a good approximation at 295 K with dry samples^4,5^.

*MB spectral fitting approach*

In MB spectroscopy, each spectral component corresponds to one Fe-bearing solid phase or to a group of unresolved Fe-bearing solid phases. These components take the form of a doublet, sextet, octet (none resolved here) or a collapsed sextet—indicating a solid-phase near its magnetic ordering temperature (T_N_). Solid-phases well above (doublet) or below (sextet) their T_N_ will not exhibit any vertical (i.e. count axis) distance between the peak troughs and the baseline. When Fe solid-phases are near their T_N_, they exhibit an intermediate shape between a doublet and full sextet, which fills the area between the upper baseline and the inverse troughs of the peaks. We approximate this by using a separate collapsed sextet component (i.e., a sextet with exceedingly large line widths and Bhf = 0 T).

Assuming equal MB recoilless fractions (i.e., that the *measurable* gamma rays emitted by each phase are proportional to the phase abundance), the total spectral area of a given component is proportional to the amount of ^57^Fe in the corresponding solid phase(s). Furthermore, it can then be assumed that each phase occupies the same spectral area at all temperatures, although not the same spectral shape. For instance, nano-scale Fe^III^‑(oxyhydr)oxide minerals form a doublet at 295 K and magnetically order to form a sextet only when cooled sufficiently to prevent thermal disordering of the macroscopic magnetic ordering within the material^6^.

We left all fitting parameters normally used to fine-tune the fit at their most general, conservative values: These include: constraining all Lorentzian half widths at twice the Heisenberg values; disallowing parameter coupling between the center shift (CS), quadrapole splitting (QS or ε, for sextets) and average hyperfine field (Bhf); and constraining the area ratios between the doublet lines to 1:1 and area ratios between sextet lines 2 and 3 to 2:1 and lines 1 and 3 to 3:1 (see Figure S2 and associated Tables). The expectation is the Lorentzian half-width values, which we set at the value optimized for an α-Fe foil standard on our spectrometer (i.e. 0.1425 mm s^‒1^), which is slightly larger than the ideal theoretical value. An explanation of the four key MB spectral parameters used in our analysis (center shift, quadrapole splitting, hyperfine field strength and the line width) is summarized in the electronic annex of Thompson et al^7^.

*MB spectral components in the heavy fraction separates of the soils*

Across the three collection temperatures (295, 77, and 5 K), we resolved five distinct spectral components. Spectral components include: (1) an Fe^III^ quadrupole doublet (labelled ferric-1) corresponding to Fe^III^ in silicates, surface‑complexed to solids, and in all Fe^III^-(oxy)hydroxides that are superparamagnetic (SP) at the collection temperature; (2) a wide Fe^II^ quadrupole doublet (labelled ferrous-1) that we attribute to paramagnetic ferrous in silicate phases; (3) a broadened Fe^III^ sextet (labelled OxHy1) that corresponds to magnetically‑ordered Fe^III^ oxyhydroxides, including ferrihydrite and goethite phases; (4) a broadened Fe^III^ sextet (labelled OX1) that corresponds to hematite phases; and (5) a partially collapsed Fe^III^ ‘sextet’ (labelled (b)Oxhy) due to a Fe^III^-(oxy)hydroxide having its SP blocking temperature near the collection temperature. In the 5 K spectra, if we assume all the Fe^III^-oxyhydroxides have magnetically ordered we can refine our component descriptions of the Fe^III^ quadrupole doublet (ferric1) to correspond to Fe^III^ complexed to solids such as organic matter or in non-Fe-rich silicates; the Fe^III^ sextets (OX and OxHy) would represents phases with clear distinctive similarity to goethite and hematite, whereas the most disordered end-members of all Fe-oxide phases would be contained in the (b)OxHy component that is represented by the partially collapsed Fe^III^ ‘sextet’ attributed to Fe^III^-(oxyhydr)oxides having their SP blocking temperatures near 5 K.

*Calculation of phase abundances*

We calculated the abundance of each Fe-bearing phase from the spectral area of each respective component in the 5 K spectra, except the ferrous component, which is better estimated from the 295 K spectra. Fe^III^-clay and Fe^II^-clay abundances are calculated from spectra area of the ferric1 and ferous1 component spectra areas of the 5 K and 295 K spectra, respectively. The total Fe^III^-(oxyhyr)oxide abundance can be calculated by summing the OX1, OxHy1, and (b)OxHy components at 5 K (Tables S3). We also calculate a crystallinity index as the ratio of full sextet areas at 77 K : 5 K, which has a maximum value of 1, with higher values indicating more crystalline phases.

**MB phase analysis of samples**

Below we provide an analysis of the five Fe populations identified in the samples. Note that all samples had all of the same spectral components and simply varied in the abundance of each component. Therefore, we fit the spectra starting with the same initial fitting parameters for each spectra at a given temperature and then refined the fit as needed for each sample. This provides a better constraint on the abundance of each phase across all samples.

*Fe^III^-oxide populations* (Hematite)

At 295 K, we detect a sextet (~10% of the spectral area) with a very large field strength (48–51 T), moderate isomer shift (0.38 mm s^-1^) and slightly negative quadrapole splitting (QS ~ ‑0.1 mm s^‒1^). This same phase maintains a similar spectral area as the temperature is decreased. At 77 K the hyperfine field strength is above 52 T and by 5 K reaches 53 T across all samples. The quadrapole splitting values increase only slightly to less negative values as the temperature increases, but never above -0.07 mm s^‒1^. This behavior is consistent with a microcrystalline hematite that is typical for those formed in soils. It does not pass through a Morin transition and remains in a weakly ferromagnetic state even at 5 K. Typically this would suggest particle sizes of the hematite below 20 nm or larger particles with considerable substitution by foreign (non-Fe) atoms. We assessed the possibility that some of this hematite could represent maghematite (which has a ~ -0.01 mm s^‒1^ quadrupole splitting value, but a similar hyperfine field strength to hematite) by measuring the magnetic susceptibility of the soils (Table S4). However, magnetic susceptibility values were generally low (see Section 3).

*Fe^III^-oxyhydroxide populations* (Goethite/nano-goethite/ferrihydrite)

Collection of MB spectra at 295, 77, and 5 K allows us to characterize the crystallinity continuum of the Fe^III^-oxyhydroxide solid phases in these soils. All resolved Fe^III^‑(oxyhydr)oxides in this soil at 295 K are superparamagnetic or near their blocking temperature. This indicates that all the goethite is less crystalline (either smaller or more disordered or both) than most synthetic goethites^8^, however as illustrated by the high proportion of ordering at 77 K, they are more crystalline than many nano-goethite phases in soils^9,10^. As the MBS collection temperature is reduced, portions of the ^57^Fe^III^-oxyhydroxide populations are resolved as they magnetically order into a sextet (Figure S2), with the more crystalline portions of the population ordering at higher temperatures. In these samples the spectral area assigned to the full Fe^III^-oxyhydroxide sextets increases from 0% to 55–75% to 64–84% at 295, 77, and 5 K, respectively. At both 77 K and 5 K we can resolve only one Fe^III^-oxyhydroxide sextet with parameters consistent with micro-crystalline or nano-goethite with QS splitting values of ‑0.11 mm s^‒1^ that are near uniform across the samples. Fe^III^‑oxyhydroxides that are blocked or near their blocking temperature at 5 K comprise 6–15% of the spectral area (Table S3). These phases likely represent even more disordered ferrihydrite or nano-goethite phases or phases that are so disordered assigning them to a bulk pure mineral phases is un-justified^7^.

*Fe^III^-silicate/organic-bound Fe populations*

Fe atoms in silicates and monomeric organic complexes are too distant from each other to order magnetically, and thus both site populations produce a doublet at 5 K with center shift values that are consistent across the samples (CS = 0.45–0.5 mm s^‒1^), but quadrapole splitting values that are more variable across the samples (QS = 0.6‒0.9 mm s^‒1^). In all cases, these parameters are consistent with octahedrally coordinated, trivalent Fe. Fe^III^ substituted layered silicates typically yields QS values in this range, with Fe-substituted kaolinite typically yielding a QS value of 0.5 mm s^‒1^ and 2:1 layer clays yielding QS values of ~0.8 mm s^‒1 11^ or higher in Fe-poor 2:1 minerals^12^.

*Paramagnetic ferrous populations*

We observe one paramagnetic ferrous contribution in these samples consistent with ilmenite. No octets consistent with the presence of magnetite are observed, although highly substituted magnetite is difficult to distinguish from other Fe oxides. The position of the high‑velocity (H_L_) line of the ferrous1 component at ~1.4 mm s^‒1^ across all samples is diagnostic for the mineral ilmenite.

**Table S3:** Summary of Fe phases and properties calculated from MB spectroscopy. Total Fe content measured with X-ray florescence spectroscopy (Fe_XRF_), dithionite-citrate-bicarbonate-extractable Fe (Fe_d_) content. Lower case letter indicate significant differences separated by land-use and depth. LL-Forest (n = 4), LH-Forest (n = 4), HL-Forest (n = 3), HH-Forest (n = 7); Cropland combinations (n = 3); mean and standard deviation in parentheses. Total pedogenic Fe represents the sum of goethite, hematite, and short ranged ordered Fe minerals, magnetically ordered (5 K) Fe includes Fe (oxyhydr)oxides that form a full sextets at 5 K. Crystalline Fe forms are defined as magnetically ordered at 77 K and we report the relative share of identified Fe mineral phases that form a full sextet at 5 K (goethite, Goe; hematite, Hem). The crystallinity index (CI) was determined by the ratio of magnetically ordered mineral phases at 77 K to those at 5 K, where higher numbers (up to a value of 1) indicate higher crystallinity for the sum of all abundant Fe phases. Each mineralogical combination and depth were analyzed as a composite sample.

| **Land-use** | **Mineralogical Combination** | **Depth** |  |  | **Fe_XRF_** | **Fe_d_** |  | **Total pedogenic**  **Fe**  **(5 K)** | **Magnetically ordered Fe**  **(5 K)** | **Crystalline Fe forms**  **(77 K)** |  | **Goe**  **(5 K)** | **Hem**  **(5 K)** | **SRO**  **(5 K)** |  | **CI** |
| --- | --- | --- | --- | --- | --- | --- | --- | --- | --- | --- | --- | --- | --- | --- | --- | --- |
|  |  | (cm) |  |  | (g kg^‒1^) | |  |  |  | (%) |  |  |  |  |  |  |
|  |  |  |  |  |  |  |  |  |  |  |  |  |  |  |  |  |
|  |  |  |  |  |  |  |  |  |  |  |  |  |  |  |  |  |
| **Forest** | **Low** aluminous clay‒ | 0‒5 |  |  | **44^c^**  (5) | **21^d^**  (4) |  | **97.6** | **87.9** | **71.3** |  | **77.5** | **10.4** | **9.7** |  | **0.81** |
|  | **Low** pedogenic Fe oxides | 5‒10 |  |  | **59^b^**  (1) | **38^b^**  (13) |  | **98.3** | **88.8** | **77.5** |  | **79.5** | **9.3** | **9.5** |  | **0.87** |
|  |  |  |  |  |  |  |  |  |  |  |  |  |  |  |  |  |
| **Forest** | **Low** aluminous clay‒ | 0‒5 |  |  | **109^a^**  (14) | **78^a^**  (14) |  | **98.5** | **91,2** | **76.6** |  | **82.1** | **9.1** | **7.3** |  | **0.84** |
|  | **High** pedogenic Fe oxides | 5‒10 |  |  | **112^a^**  (9) | **77^a^**  (4) |  | **98.6** | **91.3** | **72.0** |  | **84.2** | **7.1** | **7.3** |  | **0.79** |
|  |  |  |  |  |  |  |  |  |  |  |  |  |  |  |  |  |
| **Forest** | **High** aluminous clay‒ | 0‒5 |  |  | **59^c^**  (1) | **36^c^**  (5) |  | **97.5** | **91.6** | **70.3** |  | **77.4** | **14.2** | **5.9** |  | **0.83** |
|  | **Low** pedogenic Fe oxides | 5‒10 |  |  | **66^b^**  (4) | **44^b^**  (7) |  | **97.2** | **89.0** | **75.2** |  | **74.0** | **15.0** | **8.2** |  | **0.85** |
|  |  |  |  |  |  |  |  |  |  |  |  |  |  |  |  |  |
| **Forest** | **High** aluminous clay‒ | 0‒5 |  |  | **96^b^**  (6) | **67^b^**  (5) |  | **98.2** | **90.4** | **74.9** |  | **74.8** | **15.6** | **7.8** |  | **0.83** |
|  | **High** pedogenic Fe oxides | 5‒10 |  |  | **114^a^**  (5) | **81^a^**  (6) |  | **98,2** | **90.2** | **71.9** |  | **74.3** | **15.9** | **8.0** |  | **0.80** |
|  |  |  |  |  |  |  |  |  |  |  |  |  |  |  |  |  |
| **Cropland** | **Low** aluminous clay‒ | 0‒5 |  |  | **43^c^**  (2) | **30^c^**  (2) |  | **96.6** | **81.5** | **64.6** |  | **64.6** | **16.9** | **15.1** |  | **0.79** |
|  | **Low** pedogenic Fe oxides | 5‒10 |  |  | **43^c^**  (2) | **29^c^**  (4) |  | **97.0** | **86.0** | **69.1** |  | **69.8** | **16.2** | **11.0** |  | **0.80** |
|  |  |  |  |  |  |  |  |  |  |  |  |  |  |  |  |  |
| **Cropland** | **Low** aluminous clay‒ | 0‒5 |  |  | **133^a^**  (1) | **101^a^**  (4) |  | **98.4** | **89.2** | **77.9** |  | **72.8** | **16.4** | **9.2** |  | **0.87** |
|  | **High** pedogenic Fe oxides | 5‒10 |  |  | **137^a^**  (3) | **100^a^**  (5) |  | **98.6** | **89.8** | **77.7** |  | **72.6** | **17.2** | **8.8** |  | **0.87** |
|  |  |  |  |  |  |  |  |  |  |  |  |  |  |  |  |  |
| **Cropland** | **High** aluminous clay‒ | 0‒5 |  |  | **83^b^**  (1) | **63^b^**  (3) |  | **97.7** | **88.3** | **78.5** |  | **74.3** | **14.0** | **9.4** |  | **0.89** |
|  | **High** pedogenic Fe oxides | 5‒10 |  |  | **85^b^**  (2) | **66^b^**  (4) |  | **97.2** | **89.0** | **76.6** |  | **77.9** | **11.1** | **8.2** |  | **0.86** |
|  |  |  |  |  |  |  |  |  |  |  |  |  |  |  |  |  |

# Section 3: Magnetic susceptibility

Methodology: Mass-specific magnetic susceptibility (MS) measurements of bulk soils were collected on a Bartington MS2B Magnetic Susceptibilty system in Low-Frequency mode at the University of Georgia. Measurements were cross calibrated with a sample set measured separately Dr. Christoph Geiss (Trincoll University) on a KLY-4 Kappabridge susceptibility bridge (ASC Scientific).

Results: Mass-specific MS for these soils (χ up to 2.1 × 10^−6^ m^3^ kg^−1^) were only slightly higher than expected for the antiferromagnetic and superparamagnetic Fe^III^ phases hematite, goethite, and ferrihydrite (Table 2), which typically have χ values of ~ 5.0 × 10^−7^ m^3^ kg^−1^. This suggests only minor potential contributions of ferrimagnetic solid phases, such as magnetite or maghemite—although a ferrimagnetic ferrihydrite^13^ may be applicable here as well. Typical χ values for magnetite or maghemite are ~4.5 × 10^−4^ m^3^ kg^−1^, so even trace amounts can alter the bulk χ value for the soil and this can often occur during fire events. However, even if we assume all the MS in these soils results from maghemite (assuming a typical χ value of 4.5 × 10^−4^ m^3^ kg^−1^ maghemite) we estimate that most soils contain ~1% of total Fe as maghemite/magnetite. In a few soils, however, these minerals could account for up to 6% of total Fe (Table S4).

**Table S4:** Magnetic susceptibility (χ) of soils and possible maximum abundance of maghemite (Mgh) and magnetite (Mag), assuming that all magnetic susceptibility resulted from these minerals and a standard mass-specific magnetic susceptibility for maghemite or magnetite of 4.5 × 10^−4^ m^3^ kg^−1^. Maximum fraction of soil Fe that could possibility by maghemite- or magnetite-Fe as calculated by from the maximum possible Mag or Mgh abundance and the total Fe content (Fe_XRF_). Sample number for all combinations: *n* = 1.

| **Land-use** | **Mineralogical Combination** | **Depth** |  |  | **Fe_XRF_** |  | **Magnetic susceptibility**  **χ** |  | **Max. possible amount of Mgh or Mag** |  | **Max. Mgh or Mag fraction related to total Fe (XRF) in soil** |
| --- | --- | --- | --- | --- | --- | --- | --- | --- | --- | --- | --- |
|  |  | (cm) |  |  | (g kg_soil_^−1^) |  | (10^−8^ m^3^ kg_soil_ ^−1^) |  | (g kg_soil_^−1^) |  | (%) |
|  |  |  |  |  |  |  |  |  |  |  |  |
|  |  |  |  |  |  |  |  |  |  |  |  |
| **Forest** | **Low** aluminous clay‒ | 0‒5 |  |  | **44^c^**  (5) |  | **24** |  | **0.5** |  | **1** |
|  | **Low** pedogenic Fe oxides | 5‒10 |  |  | **59^b^**  (1) |  | **30** |  | **0.7** |  | **1** |
|  |  |  |  |  |  |  |  |  |  |  |  |
| **Forest** | **Low** aluminous clay‒ | 0‒5 |  |  | **109^a^**  (14) |  | **50** |  | **1.1** |  | **1** |
|  | **High** pedogenic Fe oxides | 5‒10 |  |  | **112^a^**  (9) |  | **57** |  | **1.3** |  | **1** |
|  |  |  |  |  |  |  |  |  |  |  |  |
| **Forest** | **High** aluminous clay‒ | 0‒5 |  |  | **59^c^**  (1) |  | **107** |  | **2.4** |  | **4** |
|  | **Low** pedogenic Fe oxides | 5‒10 |  |  | **66^b^**  (4) |  | **113** |  | **2.5** |  | **3** |
|  |  |  |  |  |  |  |  |  |  |  |  |
| **Forest** | **High** aluminous clay‒ | 0‒5 |  |  | **96^b^**  (6) |  | **52** |  | **1.2** |  | **1** |
|  | **High** pedogenic Fe oxides | 5‒10 |  |  | **114^a^**  (5) |  | **65** |  | **1.4** |  | **1** |
|  |  |  |  |  |  |  |  |  |  |  |  |
| **Cropland** | **Low** aluminous clay‒ | 0‒5 |  |  | **43^c^**  (2) |  | **111** |  | **2.5** |  | **6** |
|  | **Low** pedogenic Fe oxides | 5‒10 |  |  | **43^c^**  (2) |  | **100** |  | **2.2** |  | **5** |
|  |  |  |  |  |  |  |  |  |  |  |  |
| **Cropland** | **Low** aluminous clay‒ | 0‒5 |  |  | **133^a^**  (1) |  | **192** |  | **4.3** |  | **3** |
|  | **High** pedogenic Fe oxides | 5‒10 |  |  | **137^a^**  (3) |  | **213** |  | **4.7** |  | **3** |
|  |  |  |  |  |  |  |  |  |  |  |  |
| **Cropland** | **High** aluminous clay‒ | 0‒5 |  |  | **83^b^**  (1) |  | **153** |  | **3.8** |  | **5** |
|  | **High** pedogenic Fe oxides | 5‒10 |  |  | **85^b^**  (2) |  | **114** |  | **2.9** |  | **3** |
|  |  |  |  |  |  |  |  |  |  |  |  |

# Section 4: Density fractionation

**Table S5:** Bulk organic carbon content (Bulk OC); bulk organic carbon change compared between forest and cropland land-use (Bulk OC loss); bulk organic carbon stock (Bulk OC stock); relative mass of density fractions (Mass); organic carbon content of density fractions (Fraction OC); organic carbon loss from density fractions compared between forest and cropland land-use (Fraction OC loss); organic carbon stocks related to density fractions (Fraction OC stock), na = value not available. Lower case letters indicate significant differences within a certain land-use as separated by depth, whereas capital letters denote significant differences between land-uses. Sample numbers for the combinations are as follows: ‛low clay‒low Fe’ under forest (*n* = 4), ‛low clay‒high Fe’ under forest (*n* = 4), ‛high clay‒low Fe’ under forest (*n* = 3), ‛high clay‒high Fe’ under forest (*n* = 7); all cropland combinations (*n* = 3).

| **Land-use** | **Mineralogical Combination** | **Depth** |  |  | **Bulk**  **OC** |  | **Bulk**  **OC**  **loss** |  | **Bulk**  **OC**  **stock** |  |  | **Mass** |  |  | **Fraction OC** | | |  | **Fraction OC loss** | | |  | **Fraction OC stock** | | |
| --- | --- | --- | --- | --- | --- | --- | --- | --- | --- | --- | --- | --- | --- | --- | --- | --- | --- | --- | --- | --- | --- | --- | --- | --- | --- |
|  |  |  |  |  |  |  |  |  |  |  | fLF | oLF | HF |  | fLF | oLF | HF  (MAOC) |  | fLF | oLF | HF  (MAOC) |  | fLF | oLF | HF  (MAOC) |
|  |  | (cm) |  |  | (g kg^‒1^) |  | (%) |  | (kg m^‒2^) |  |  | (%) |  |  |  | (g kg_soil_^‒1^) |  |  |  | (%) |  |  |  | (kg m^‒2^) |  |
|  |  |  |  |  |  |  |  |  |  |  |  |  |  |  |  |  |  |  |  |  |  |  |  |  |  |
|  |  |  |  |  |  |  |  |  |  |  |  |  |  |  |  |  |  |  |  |  |  |  |  |  |  |
| **Forest** | **Low** aluminous clay‒ | 0‒5 |  |  | **76.0^ab, A^**  (27.4) |  | na |  | **4.0^a, A^**  (1.3) |  | **10.1^a, A^**  (5.8) | **2.0^ab, A^**  (1.3) | **87.8^b, A^**  (7.0) |  | **33.8^a, A^**  (20.8) | **8.4^b, A^**  (5.4) | **27.1^a, A^**  (2.9) |  | na | na | na |  | **1.8^a, A^**  (1.0) | **0.4^ab, A^**  (0.3) | **1.4^a, A^**  (0.2) |
|  | **Low** pedogenic Fe oxides | 5‒10 |  |  | **34.1^a, A^**  (6.2) |  | na |  | **1.7^a, A^**  (0.4) |  | **0.7^ab, A^**  (0.2) | **0.9^ab, A^**  (0.3) | **98.4^ab, A^**  (0.6) |  | **2.4^ab, A^**  (0.9) | **3.0^a, A^**  (1.1) | **23.2^ab, A^**  (5.5) |  | na | na | na |  | **0.1^a, A^**  (0.0) | **0.2^a, A^**  (0.1) | **1.2^a, A^**  (0.3) |
|  |  |  |  |  |  |  |  |  |  |  |  |  |  |  |  |  |  |  |  |  |  |  |  |  |  |
| **Forest** | **Low** aluminous clay‒ | 0‒5 |  |  | **57.3^b, A^**  (14.4) |  | na |  | **2.3^a, A^**  (0.4) |  | **3.0^b, A^**  (1.9) | **1.7^b, A^**  (0.8) | **95.4^a, A^**  (2.6) |  | **10.8^ab, A^**  (5.4) | **6.9^b, A^**  (3.2) | **36.0^a, A^**  (4.9) |  | na | na | na |  | **0.4^b, A^**  (0.2) | **0.3^b, A^**  (0.1) | **1.4^a, B^**  (0.2) |
|  | **High** pedogenic Fe oxides | 5‒10 |  |  | **37.2^a, A^**  (6.6) |  | na |  | **1.4^ab, B^**  (0.2) |  | **0.9^a, A^**  (0.1) | **1.1^a, A^**  (0.5) | **98.0^b, A^**  (0.6) |  | **3.5^a, A^**  (0.5) | **4.0^a, A^**  (2.1) | **28.3^a, B^**  (5.3) |  | na | na | na |  | **0.1^a, A^**  (0.0) | **0.2^a, A^**  (0.1) | **1.1^ab, B^**  (0.1) |
|  |  |  |  |  |  |  |  |  |  |  |  |  |  |  |  |  |  |  |  |  |  |  |  |  |  |
| **Forest** | **High** aluminous clay‒ | 0‒5 |  |  | **43.2^b^**  (6.1) |  | na |  | **1.9^a^**  (0.3) |  | **1.8^b^**  (0.4) | **1.3^b^**  (0.3) | **96.9^a^**  (0.7) |  | **6.1^b^**  (1.7) | **5.7^b^**  (1.3) | **24.5^a^**  (1.1) |  | na | na | na |  | **0.3^b^**  (0.1) | **0.3^b^**  (0.1) | **1.1^a^**  (0.1) |
|  | **Low** pedogenic Fe oxides | 5‒10 |  |  | **23.0^b^**  (5.0) |  | na |  | **1.0^b^**  (0.2) |  | **0.3^b^**  (0.3) | **0.6^b^**  (0.1) | **99.1^a^**  (0.3) |  | **1.4^b^**  (1.3) | **2.2^a^**  (0.5) | **14.6^c^**  (0.8) |  | na | na | na |  | **0.1^b^**  (0.1) | **0.1^a^**  (0.0) | **0.7^c^**  (0.1) |
|  |  |  |  |  |  |  |  |  |  |  |  |  |  |  |  |  |  |  |  |  |  |  |  |  |  |
| **Forest** | **High** aluminous clay‒ | 0‒5 |  |  | **95.1^a, A^**  (31.1) |  | na |  | **3.9^a, A^**  (1.8) |  | **7.8^ab, A^**  (5.3) | **6.9^a, A^**  (5.2) | **85.3^b, B^**  (5.1) |  | **25.7^ab, A^**  (19.3) | **19.2^a, A^**  (9.9) | **36.1^a, A^**  (15.7) |  | na | na | na |  | **1.1^ab, A^**  (1.1) | **0.7^a, A^**  (0.3) | **1.5^a, A^**  (0.8) |
|  | **High** pedogenic Fe oxides | 5‒10 |  |  | **34.9^a, A^**  (4.5) |  | na |  | **1.4^ab, A^**  (0.2) |  | **0.7^a, A^**  (0.2) | **0.9^ab, A^**  (0.2) | **98.4^ab, A^**  (0.3) |  | **2.7^a, A^**  (0.9) | **2.5^a, A^**  (0.4) | **22.6^b, A^**  (2.6) |  | na | na | na |  | **0.1^ab, A^**  (0.0) | **0.1^a, B^**  (0.0) | **0.9^bc, A^**  (0.2) |
|  |  |  |  |  |  |  |  |  |  |  |  |  |  |  |  |  |  |  |  |  |  |  |  |  |  |
| **Cropland** | **Low** aluminous clay‒ | 0‒5 |  |  | **18.7^c, B^**  (0.2) |  | **-73**  (10) |  | **1.1^c, B^**  (0.0) |  | **0.3^c, B^**  (0.0) | **1.8^a, A^**  (1.3) | **97.9^a, A^**  (1.3) |  | **1.1^b, A^**  (0.1) | **4.4^a, A^**  (2.4) | **10.5^c, B^**  (2.7) |  | **-97** | **-48** | **-61** |  | **0.1^b, B^**  (0.0) | **0.3^a, A^**  (0.1) | **0.6^c, B^**  (0.2) |
|  | **Low** pedogenic Fe oxides | 5‒10 |  |  | **18.8^c, B^**  (1.3) |  | **-44**  (14) |  | **1.1^c, B^**  (0.1) |  | **0.3^b, B^**  (0.1) | **0.5^c, A^**  (0.0) | **99.3^a, A^**  (0.1) |  | **1.0^c, B^**  (0.2) | **2.1^b, A^**  (0.1) | **13.7^c, B^**  (0.2) |  | **-58** | **-30** | **-41** |  | **0.1^c, A^**  (0.0) | **0.1^b, A^**  (0.0) | **0.8^b, A^**  (0.0) |
|  |  |  |  |  |  |  |  |  |  |  |  |  |  |  |  |  |  |  |  |  |  |  |  |  |  |
| **Cropland** | **Low** aluminous clay‒ | 0‒5 |  |  | **47.1^a, A^**  (0.9) |  | **-14**  (18) |  | **2.4^a, A^**  (0.1) |  | **1.3^a, A^**  (0.2) | **1.3^a, A^**  (0.5) | **97.4^a, A^**  (0.6) |  | **3.6^a, A^**  (0.4) | **5.7^a, A^**  (1.9) | **38.2^a, A^**  (4.7) |  | **-67** | **-17** | **+6** |  | **0.2^a, A^**  (0.0) | **0.3^a, A^**  (0.1) | **1.9^a, A^**  (0.2) |
|  | **High** pedogenic Fe oxides | 5‒10 |  |  | **48.1^a,A^**  (4.8) |  | **+35**  (18) |  | **2.5^a, A^**  (0.2) |  | **0.8^a, A^**  (0.1) | **1.0^a, A^**  (0.2) | **98.2^c, A^**  (0.2) |  | **2.9^a, A^**  (0.3) | **4.2^a, A^**  (0.7) | **36.7^a, A^**  (1.3) |  | **-17** | **+5** | **+30** |  | **0.2^a, A^**  (0.0) | **0.2^a, A^**  (0.0) | **1.9^a, A^**  (0.0) |
|  |  |  |  |  |  |  |  |  |  |  |  |  |  |  |  |  |  |  |  |  |  |  |  |  |  |
| **Cropland** | **High** aluminous clay‒ | 0‒5 |  |  | **33.7^b,B^**  (1.2) |  | **-62**  (9) |  | **1.7^b, A^**  (0.1) |  | **1.1^b, A^**  (0.1) | **0.8^a, A^**  (0.1) | **98.2^a, A^**  (0.0) |  | **3.4^a, A^**  (0.3) | **3.6^a, B^**  (0.4) | **20.0^b, A^**  (0.9) |  | **-87** | **-81** | **-45** |  | **0.2^a, A^**  (0.0) | **0.2^a, B^**  (0.0) | **1.0^b, A^**  (0.1) |
|  | **High** pedogenic Fe oxides | 5‒10 |  |  | **28.9^b,A^**  (2.9) |  | **-15**  (15) |  | **1.4^b, A^**  (0.1) |  | **0.6^a, A^**  (0.2) | **0.7^b, A^**  (0.0) | **98.7^b, A^**  (0.3) |  | **2.0^b, A^**  (0.7) | **2.9^b, A^**  (0.2) | **18.8^b, A^**  (3.0) |  | **-26** | **+16** | **-17** |  | **0.1^b, A^**  (0.0) | **0.1^b, A^**  (0.0) | **0.9^b, A^**  (0.1) |
|  |  |  |  |  |  |  |  |  |  |  |  |  |  |  |  |  |  |  |  |  |  |  |  |  |  |

# Section 5: Model parameters

Table S6: Linear and multiple linear relationships of aluminous clay, dithionite-citrate-bicarbonate-extractable Fe (Fe_d_), Fe_d_ to aluminous clay ratio, and oxalate-extractable Fe (Fe_o_) to bulk organic carbon (Bulk OC), OC mineralization (OC_min_), free light fraction OC, occluded light fraction OC, mineral-associated OC (MAOC), MAOC resistant to 6% NaOCl treatment, and OC mineralization of MAOC. Aluminous clay represents the weight sum of kaolinite and gibbsite present in the < 2-µm fraction after removal of OM and pedogenic Fe.

|  |  | **Aluminous clay** | **Fe_d_** | **Fe_d_/clay** | **Fe_o_** | **OC_min_** |  | **Intercept** | **Degree of freedom** | **F-value** | **r^2^**  **(%)** | **P**  **value** |
| --- | --- | --- | --- | --- | --- | --- | --- | --- | --- | --- | --- | --- |
| Dependent variables |  | Independent variables | | | | |  | Model parameters | | | | |
|  |  |  |  |  |  |  |  |  |  |  |  |  |
|  |  |  |  |  |  |  |  |  |  |  |  |  |
| **Bulk OC** |  |  |  |  |  |  |  |  |  |  |  |  |
| OC_Forest_0‒5 cm_ |  | 0.13 |  |  |  |  |  | 39.88 | 18 | 2.35 | 7 | 0.15 |
| OC_Forest_5‒10 cm_ |  | -0.03 |  |  |  |  |  | 41.81 | 18 | 3.69 | 14 | 0.07 |
| OC_Cropland_0‒5 cm_ |  | -0.01 |  |  |  |  |  | 35.53 | 9 | 0.04 | < 1 | 0.85 |
| OC_Cropland_5‒10 cm_ |  | -0.02 |  |  |  |  |  | 36.66 | 9 | 0.14 | < 1 | 0.72 |
| OC_Forest+Cropland_0‒5 cm_ |  | 0.02 |  |  |  |  |  | 53.68 | 27 | 0.11 | < 1 | 0.74 |
| OC_Forest+Cropland_5‒10 cm_ |  | -0.03 |  |  |  |  |  | 39.97 | 27 | 2.09 | 4 | 0.16 |
| OC_Forest+Cropland_0‒10 cm_ |  | -0.02 |  |  |  |  |  | 51.03 | 54 | 0.22 | < 1 | 0.64 |
|  |  |  |  |  |  |  |  |  |  |  |  |  |
| OC_Forest_0‒5 cm_ |  |  | -0.002 |  |  |  |  | 72.90 | 18 | < 0.01 | < 1 | 0.99 |
| OC_Forest_5‒10 cm_ |  |  | 0.12 |  |  |  |  | 25.82 | 18 | 2.24 | 7 | 0.15 |
| OC_Cropland_0‒5 cm_ |  |  | 0.40 |  |  |  |  | 7.61 | 9 | 914 | 99 | 0.001 |
| OC_Cropland_5‒10 cm_ |  |  | 0.41 |  |  |  |  | 5.67 | 9 | 54.15 | 87 | 0.001 |
| OC_Forest+Cropland_0‒5 cm_ |  |  | 0.08 |  |  |  |  | 54.91 | 27 | 0.10 | < 1 | 0.76 |
| OC_Forest+Cropland_5‒10 cm_ |  |  | 0.26 |  |  |  |  | 16.01 | 27 | 21.17 | 44 | 0.001 |
| OC_Forest+Cropland_0‒10 cm_ |  |  | 0.11 |  |  |  |  | 39.49 | 54 | 0.52 | < 1 | 0.47 |
|  |  |  |  |  |  |  |  |  |  |  |  |  |
| OC_Forest_0‒5 cm_ |  | 0.14 | -0.08 |  |  |  |  | 43.51 | 18 | 1.14 | 2 | 0.35 |
| OC_Forest_5‒10 cm_ |  | -0.04 | 0.15 |  |  |  |  | 33.92 | 18 | 4.84 | 31 | 0.02 |
| OC_Cropland_0‒5 cm_ |  | 0.01 | 0.40 |  |  |  |  | 5.45 | 9 | 778.4 | 99 | 0.001 |
| OC_Cropland_5‒10 cm_ |  | -0.02 | 0.41 |  |  |  |  | 10.59 | 9 | 29.35 | 88 | 0.001 |
| OC_Forest+Cropland_0‒5 cm_ |  | 0.02 | 0.07 |  |  |  |  | 49.50 | 27 | 0.10 | < 1 | 0.91 |
| OC_Forest+Cropland_5‒10 cm_ |  | -0.03 | 0.28 |  |  |  |  | 24.31 | 27 | 17.22 | 56 | 0.001 |
| OC_Forest+Cropland_0‒10 cm_ |  | -0.02 | 0.12 |  |  |  |  | 44.61 | 54 | 0.40 | < 1 | 0.68 |
|  |  |  |  |  |  |  |  |  |  |  |  |  |
| OC_Forest_0‒5 cm_ |  |  |  | -55.47 |  |  |  | 86.78 | 18 | 1.22 | < 1 | 0.29 |
| OC_Forest_5‒10 cm_ |  |  |  | 20.78 |  |  |  | 27.81 | 18 | 3.32 | 12 | 0.09 |
| OC_Cropland_0‒5 cm_ |  |  |  | 55.19 |  |  |  | 18.64 | 9 | 18.66 | 69 | 0.004 |
| OC_Cropland_5‒10 cm_ |  |  |  | 68.17 |  |  |  | 14.67 | 9 | 20.21 | 71 | 0.003 |
| OC_Forest+Cropland_0‒5 cm_ |  |  |  | -9.94 |  |  |  | 62.11 | 27 | 0.06 | < 1 | 0.81 |
| OC_Forest+Cropland_5‒10 cm_ |  |  |  | 40.18 |  |  |  | 22.40 | 27 | 16.56 | 37 | 0.001 |
| OC_Forest+Cropland_0‒10 cm_ |  |  |  | 12.08 |  |  |  | 43.07 | 54 | 0.24 | < 1 | 0.63 |
|  |  |  |  |  |  |  |  |  |  |  |  |  |
| OC_Forest_0‒5 cm_ |  |  |  |  | 22.14 |  |  | 46.54 | 18 | 0.58 | < 1 | 0.46 |
| OC_Forest_5‒10 cm_ |  |  |  |  | 10.90 |  |  | 18.71 | 18 | 4.85 | 19 | 0.04 |
| OC_Cropland_0‒5 cm_ |  |  |  |  | 30.76 |  |  | -0.67 | 9 | 704.5 | 99 | 0.001 |
| OC_Cropland_5‒10 cm_ |  |  |  |  | 26.66 |  |  | 0.83 | 9 | 45.89 | 85 | 0.001 |
| OC_Forest+Cropland_0‒5 cm_ |  |  |  |  | 34.58 |  |  | 19.56 | 27 | 3.10 | 8 | 0.09 |
| OC_Forest+Cropland_5‒10 cm_ |  |  |  |  | 18.49 |  |  | 9.16 | 27 | 28.68 | 52 | 0.001 |
| OC_Forest+Cropland_0‒10 cm_ |  |  |  |  | 17.01 |  |  | 25.48 | 54 | 2.52 | 3 | 0.12 |
|  |  |  |  |  |  |  |  |  |  |  |  |  |
| **Free light fraction OC** |  |  |  |  |  |  |  |  |  |  |  |  |
| OC_Forest_0‒5 cm_ |  | 0.03 | -0.24 |  |  |  |  | 25.04 | 18 | 0.92 | < 1 | 0.42 |
| OC_Forest_5‒10 cm_ |  | -0.01 | 0.03 |  |  |  |  | 2.65 | 18 | 7.14 | 42 | 0.007 |
| OC_Cropland_0‒5 cm_ |  | 0.01 | 0.04 |  |  |  |  | -1.16 | 9 | 62.16 | 94 | 0.001 |
| OC_Cropland_5‒10 cm_ |  | 0.00 | 0.00 |  |  |  |  | 0.02 | 9 | 14.5 | 77 | 0.005 |
| OC_Forest+Cropland_0‒5 cm_ |  | 0.00 | -0.14 |  |  |  |  | 23.2 | 27 | 0.65 | < 1 | 0.53 |
| OC_Forest+Cropland_5‒10 cm_ |  | 0.00 | 0.03 |  |  |  |  | 1.85 | 27 | 9.7 | 40 | 0.001 |
|  |  |  |  |  |  |  |  |  |  |  |  |  |
| **Occluded light fraction OC** |  |  |  |  |  |  |  |  |  |  |  |  |
| OC_Forest_0‒5 cm_ |  | 0.04 | 0.06 |  |  |  |  | -0.77 | 18 | 1.37 | 4 | 0.28 |
| OC_Forest_5‒10 cm_ |  | -0.01 | 0.01 |  |  |  |  | 4.04 | 18 | 1.73 | 8 | 0.21 |
| OC_Cropland_0‒5 cm_ |  | -0.01 | 0.02 |  |  |  |  | 5.04 | 9 | 0.97 | < 1 | 0.43 |
| OC_Cropland_5‒10 cm_ |  | 0.00 | 0.03 |  |  |  |  | 1.36 | 9 | 10.33 | 70 | 0.01 |
| OC_Forest+Cropland_0‒5 cm_ |  | 0.01 | 0.03 |  |  |  |  | 5.00 | 27 | 0.29 | < 1 | 0.75 |
| OC_Forest+Cropland_5‒10 cm_ |  | 0.00 | 0.02 |  |  |  |  | 2.95 | 27 | 3.25 | 15 | 0.06 |
|  |  |  |  |  |  |  |  |  |  |  |  |  |
| **Heavy fraction OC (MAOC)** |  |  |  |  |  |  |  |  |  |  |  |  |
| OC_Forest_0‒5 cm_ |  | 0.03 |  |  |  |  |  | 26.42 | 18 | 0.58 | < 1 | 0.46 |
| OC_Forest_5‒10 cm_ |  | -0.03 |  |  |  |  |  | 32.04 | 18 | 8.28 | 30 | 0.01 |
| OC_Cropland_0‒5 cm_ |  | -0.03 |  |  |  |  |  | 32.35 | 9 | 0.68 | < 1 | 0.44 |
| OC_Cropland_5‒10 cm_ |  | -0.03 |  |  |  |  |  | 30.85 | 9 | 0.65 | < 1 | 0.45 |
| OC_Forest+Cropland_0‒5 cm_ |  | -0.01 |  |  |  |  |  | 32.62 | 27 | 0.22 | < 1 | 0.64 |
| OC_Forest+Cropland_5‒10 cm_ |  | -0.03 |  |  |  |  |  | 31.50 | 27 | 5.38 | 14 | 0.03 |
| OC_Forest+Cropland_0‒10 cm_ |  | -0.03 |  |  |  |  |  | 33.07 | 54 | 3.07 | 4 | 0.09 |
|  |  |  |  |  |  |  |  |  |  |  |  |  |
| OC_Forest_0‒5 cm_ |  |  | 0.15 |  |  |  |  | 23.86 | 18 | 1.87 | 5 | 0.19 |
| OC_Forest_5‒10 cm_ |  |  | 0.09 |  |  |  |  | 16.66 | 18 | 2.20 | 7 | 0.16 |
| OC_Cropland_0‒5 cm_ |  |  | 0.38 |  |  |  |  | -1.77 | 9 | 62.01 | 88 | 0.001 |
| OC_Cropland_5‒10 cm_ |  |  | 0.32 |  |  |  |  | 2.36 | 9 | 42.2 | 84 | 0.001 |
| OC_Forest+Cropland_0‒5 cm_ |  |  | 0.23 |  |  |  |  | 15.7 | 27 | 7.57 | 20 | 0.01 |
| OC_Forest+Cropland_5‒10 cm_ |  |  | 0.21 |  |  |  |  | 9.47 | 27 | 20.14 | 42 | 0.001 |
| OC_Forest+Cropland_0‒10 cm_ |  |  | 0.20 |  |  |  |  | 13.48 | 54 | 15.29 | 21 | 0.001 |
|  |  |  |  |  |  |  |  |  |  |  |  |  |
| OC_Forest_0‒5 cm_ |  | 0.02 | 0.14 |  |  |  |  | 19.85 | 18 | 1.06 | < 1 | 0.37 |
| OC_Forest_5‒10 cm_ |  | -0.04 | 0.13 |  |  |  |  | 25.20 | 18 | 10.1 | 52 | 0.002 |
| OC_Cropland_0‒5 cm_ |  | -0.02 | 0.37 |  |  |  |  | 4.29 | 9 | 38.37 | 90 | 0.001 |
| OC_Cropland_5‒10 cm_ |  | -0.03 | 0.32 |  |  |  |  | 10.29 | 9 | 52.46 | 93 | 0.001 |
| OC_Forest+Cropland_0‒5 cm_ |  | -0.02 | 0.23 |  |  |  |  | 19.57 | 27 | 3.92 | 18 | 0.034 |
| OC_Forest+Cropland_5‒10 cm_ |  | -0.04 | 0.23 |  |  |  |  | 18.77 | 27 | 28.11 | 68 | 0.001 |
| OC_Forest+Cropland_0‒10 cm_ |  | -0.03 | 0.21 |  |  |  |  | 21.15 | 54 | 11.27 | 28 | 0.001 |
|  |  |  |  |  |  |  |  |  |  |  |  |  |
| OC_Forest_0‒5 cm_ |  |  |  | 16.79 |  |  |  | 28.40 | 18 | 0.83 | < 1 | 0.38 |
| OC_Forest_5‒10 cm_ |  |  |  | 25.05 |  |  |  | 16.09 | 18 | 9.74 | 34 | 0.007 |
| OC_Cropland_0‒5 cm_ |  |  |  | 61.91 |  |  |  | 6.59 | 9 | 56.71 | 87 | 0.001 |
| OC_Cropland_5‒10 cm_ |  |  |  | 59.59 |  |  |  | 7.96 | 9 | 54.2 | 87 | 0.001 |
| OC_Forest+Cropland_0‒5 cm_ |  |  |  | 25.96 |  |  |  | 20.18 | 27 | 6.96 | 19 | 0.01 |
| OC_Forest+Cropland_5‒10 cm_ |  |  |  | 39.05 |  |  |  | 12.67 | 27 | 34.94 | 57 | 0.001 |
| OC_Forest+Cropland_0‒10 cm_ |  |  |  | 37.14 |  |  |  | 16.52 | 54 | 20.32 | 27 | 0.001 |
|  |  |  |  |  |  |  |  |  |  |  |  |  |
| OC_Forest_0‒5 cm_ |  |  |  |  | 10.82 |  |  | 19.82 | 18 | 1.08 | < 1 | 0.32 |
| OC_Forest_5‒10 cm_ |  |  |  |  | 8.02 |  |  | 11.95 | 18 | 3.77 | 14 | 0.07 |
| OC_Cropland_0‒5 cm_ |  |  |  |  | 29.40 |  |  | -9.45 | 9 | 51.41 | 86 | 0.001 |
| OC_Cropland_5‒10 cm_ |  |  |  |  | 20.15 |  |  | -0.47 | 9 | 23.96 | 74 | 0.002 |
| OC_Forest+Cropland_0‒5 cm_ |  |  |  |  | 22.45 |  |  | 3.42 | 27 | 11.53 | 29 | 0.002 |
| OC_Forest+Cropland_5‒10 cm_ |  |  |  |  | 13.48 |  |  | 5.55 | 27 | 19.59 | 42 | 0.001 |
| OC_Forest+Cropland_0‒10 cm_ |  |  |  |  | 14.79 |  |  | 8.07 | 54 | 15.24 | 21 | 0.001 |
|  |  |  |  |  |  |  |  |  |  |  |  |  |
| **Bulk OC mineralization** |  |  |  |  |  |  |  |  |  |  |  |  |
| OC__Forest+Cropland_0‒5 cm_ |  |  |  | -5.35 |  |  |  | 5.62 | 7 | 1.85 | 12 | 0.23 |
| OC__ Forest+Cropland_5‒10 cm_ |  |  |  | -7.32 |  |  |  | 6.04 | 7 | 3.58 | 30 | 0.12 |
| OC__ Forest+Cropland_0‒10 cm_ |  |  |  | -6.25 |  |  |  | 5.80 | 14 | 6.09 | 28 | 0.03 |
|  |  |  |  |  |  |  |  |  |  |  |  |  |
| **MAOC_remain_ (6% NaOCl)** |  |  |  |  |  |  |  |  |  |  |  |  |
| OC_Forest+Cropland_0‒5 cm_ |  | -0.01 | 0.18 |  |  |  |  | 6.87 | 7 | 6.75 | 66 | 0.05 |
| OC_Forest+Cropland_5‒10 cm_ |  | -0.02 | 0.16 |  |  |  |  | 5.50 | 7 | 7.80 | 69 | 0.04 |
| OC_Forest+Cropland_0‒10 cm_ |  | -0.02 | 0.16 |  |  |  |  | 6.73 | 14 | 14.38 | 67 | 0.000 |
|  |  |  |  |  |  |  |  |  |  |  |  |  |
| OC_Forest+Cropland_0‒5 cm_ |  |  |  | 28.69 |  |  |  | 5.81 | 7 | 9.87 | 60 | 0.03 |
| OC_Forest+Cropland_5‒10 cm_ |  |  |  | 25.77 |  |  |  | 4.32 | 7 | 10.04 | 60 | 0.03 |
| OC_Forest+Cropland_0‒10 cm_ |  |  |  | 27.09 |  |  |  | 5.09 | 14 | 20.14 | 60 | 0.001 |
|  |  |  |  |  |  |  |  |  |  |  |  |  |
| OC_Forest+Cropland_0‒5 cm_ |  |  |  |  |  | -2.74 |  | 24.65 | 7 | 8.93 | 57 | 0.03 |
| OC_Forest+Cropland_5‒10 cm_ |  |  |  |  |  | -1.86 |  | 18.68 | 7 | 4.06 | 34 | 0.1 |
| OC_Forest+Cropland_0‒10 cm_ |  |  |  |  |  | -2.27 |  | 21.50 | 14 | 12.51 | 47 | 0.004 |
|  |  |  |  |  |  |  |  |  |  |  |  |  |

# Section 6: Specific surface area analysis and chemical resistance of MAOC

**Table S7:** Fe_d_ to aluminous clay ratio, specific surface area (SSA), mineral-associated OC (MAOC), mineral-associated OC resistant to oxidation with 6% NaOCl (MAOC_remain_; see Method section), and relative amount of OC removed from the heavy fraction. Lower case letters indicate significant differences within a certain land-use separated by depth, whereas capital letters denote significant differences between land-uses. Sample numbers for the combinations are as follows: ‛low clay‒low Fe’ under forest (*n* = 4), ‛low clay‒high Fe’ under forest (*n* = 4), ‛high clay‒low Fe’ under forest (*n* = 3), ‛high clay‒high Fe’ under forest (*n* = 7); all cropland combinations (*n* = 3). SSA, and MAOC_remain_ (for each mineralogical combination and depth; *n* = 2).

| **Land-use** | **Mineralogical Combination** | **Depth** |  |  | **Fe_d_/clay** |  | **SSA** |  | **MAOC** |  | **MAOC_remain_** | | |
| --- | --- | --- | --- | --- | --- | --- | --- | --- | --- | --- | --- | --- | --- |
|  |  | (cm) |  |  |  |  | (m^2^ g^‒1^) |  | (g kg^‒1^) |  | (g kg^‒1^) |  | (%) |
|  |  |  |  |  |  |  |  |  |  |  |  |  |  |
|  |  |  |  |  |  |  |  |  |  |  |  |  |  |
| **Forest** | **Low** aluminous clay‒ | 0‒5 |  |  | **0.15^b, A^**  (0.04) |  | **10.1^b, B^**  (0.7) |  | **27.1^a, A^**  (2.9) |  | **10.9**  (0.7) |  | **40** |
|  | **Low** pedogenic Fe oxides | 5‒10 |  |  | **0.21^bc, A^**  (0.09) |  | **18.0^c, A^**  (0.09) |  | **23.2^ab, A^**  (0.09) |  | **10.2**  (0.8) |  | **44** |
|  |  |  |  |  |  |  |  |  |  |  |  |  |  |
| **Forest** | **Low** aluminous clay‒ | 0‒5 |  |  | **0.45^a, A^**  (0.12) |  | **23.9^a, B^**  (2.1) |  | **36.0^a, A^**  (4.9) |  | **17.0**  (2.2) |  | **47** |
|  | **High** pedogenic Fe oxides | 5‒10 |  |  | **047^a, A^**  (0.13) |  | **28.2^b, B^**  (2.7) |  | **28.3^a, B^**  (5.3) |  | **11.9**  (1.7) |  | **42** |
|  |  |  |  |  |  |  |  |  |  |  |  |  |  |
| **Forest** | **High** aluminous clay‒ | 0‒5 |  |  | **0.12^b^**  (0.01) |  | **27.8^a^**  (7.2) |  | **24.5^a^**  (1.1) |  | **8.7**  (0.9) |  | **36** |
|  | **Low** pedogenic Fe oxides | 5‒10 |  |  | **0.12^c^**  (0.02 |  | **26.1^b^**  (2.6) |  | **14.6^c^**  (0.8) |  | **8.6**  (1.1) |  | **59** |
|  |  |  |  |  |  |  |  |  |  |  |  |  |  |
| **Forest** | **High** aluminous clay‒ | 0‒5 |  |  | **0.22^b, A^**  (0.03) |  | **28.7^a, A^**  (3.2) |  | **36.1^a, A^**  (15.7) |  | **18.1**  (1.3) |  | **50** |
|  | **High** pedogenic Fe oxides | 5‒10 |  |  | **0.23^b, A^**  (0.02) |  | **36.4^a, A^**  (2.6) |  | **22.6^b, A^**  (2.6) |  | **11.3**  (0.7) |  | **50** |
|  |  |  |  |  |  |  |  |  |  |  |  |  |  |
| **Cropland** | **Low** aluminous clay‒ | 0‒5 |  |  | **0.13^b, A^**  (0.01) |  | **21.0^b, A^**  (0.6) |  | **10.5^c, B^**  (2.7) |  | **4.4**  (0.1) |  | **42** |
|  | **Low** pedogenic Fe oxides | 5‒10 |  |  | **0.14^b, A^**  (0.03) |  | **17.0^c, A^**  (0.4) |  | **13.7^c, B^**  (0.2) |  | **5.5**  (0.6) |  | **40** |
|  |  |  |  |  |  |  |  |  |  |  |  |  |  |
| **Cropland** | **Low** aluminous clay‒ | 0‒5 |  |  | **0.51^a, A^**  (0.06) |  | **32.1^a, A^**  (1.2) |  | **38.2^a, A^**  (4.7) |  | **20.5**  (1.9) |  | **54** |
|  | **High** pedogenic Fe oxides | 5‒10 |  |  | **0.47^a, A^**  (0.07) |  | **35.9^a, A^**  (1.6) |  | **36.7^a, A^**  (1.3) |  | **20.5**  (0.0) |  | **56** |
|  |  |  |  |  |  |  |  |  |  |  |  |  |  |
| **Cropland** | **High** aluminous clay‒ | 0‒5 |  |  | **0.15^b, B^**  (0.01) |  | **31.7^a, A^**  (1.2) |  | **20.0^b, A^**  (0.9) |  | **10.3**  (0.9) |  | **52** |
|  | **High** pedogenic Fe oxides | 5‒10 |  |  | **0.15^b, B^**  (0.01) |  | **30.2^b, B^**  (2.0) |  | **18.8^b, A^**  (3.0) |  | **8.4**  (1.0) |  | **45** |
|  |  |  |  |  |  |  |  |  |  |  |  |  |  |

# Section 7: ^13^C NMR analysis

Table S8: Chemical composition of forest and cropland litter material (Oi horizons) as derived from solid-state CPMAS ^13^C-NMR spectroscopy. Capital letters indicate significant differences between land-uses. Sample numbers were *n* = 7 for forests and *n* = 3 for croplands; mean and standard deviation in parentheses.

| **Land-use** |  |  | **Alkyl C** |  | **O/N-Alkyl C** |  | **Aryl C** |  | **Carboxyl C** |  | **Alkyl C / O/N-Alkyl C** |
| --- | --- | --- | --- | --- | --- | --- | --- | --- | --- | --- | --- |
|  |  |  | 0‒45 ppm |  | 45‒110 ppm |  | 110‒160 ppm |  | 160‒220 ppm |  |  |
|  |  |  | (%) | | | | | | |  |  |
|  |  |  |  |  |  |  |  |  |  |  |  |
|  |  |  |  |  |  |  |  |  |  |  |  |
| **Forest**  **Litter** |  |  | **19.4^A^**  (1.4) |  | **59.6^B^**  (3.0) |  | **17.4^A^**  (1.3) |  | **3.9^A^**  (2.4) |  | **0.3^A^**  (0.0) |
|  |  |  |  |  |  |  |  |  |  |  |  |
| **Cropland**  **Litter** |  |  | **13.8^B^**  (2.4) |  | **81.8^A^**  (9.4) |  | **8.0^B^**  (4.1) |  | **0.6^A^**  (1.1) |  | **0.2^B^**  (0.0) |
|  |  |  |  |  |  |  |  |  |  |  |  |

# Section 8: Microbial respiration

**Table S9:** Cumulative OC mineralization differentiated by selected time intervals. Lower case letters indicate significant differences within a certain land-use separated by depth, whereas capital letters denote significant differences between land-uses. Sample numbers for the combinations are as follows: ‛low clay‒low Fe’ under forest (*n* = 4), ‛low clay‒high Fe’ under forest (*n* = 4), ‛high clay‒low Fe’ under forest (*n* = 3), ‛high clay‒high Fe’ under forest (*n* = 7); all cropland combinations (*n* = 3).

| **Land-use** | **Mineralogical Combination** | **Depth** |  |  | **Cumulative OC _mineralization_** | | | | |  | **Relative share** | | |
| --- | --- | --- | --- | --- | --- | --- | --- | --- | --- | --- | --- | --- | --- |
|  |  |  |  |  | 0‒7 days |  | 8‒50 days |  | 0‒50 days |  | 0‒7 days |  | 8‒50 days |
|  |  | (cm) |  |  | (mg CO_2_-C g_OC_^‒1^) | | | | |  | (%) | | |
|  |  |  |  |  |  |  |  |  |  |  |  |  |  |
|  |  |  |  |  |  |  |  |  |  |  |  |  |  |
| **Forest** | **Low** aluminous clay‒ | 0‒5 |  |  | **1.0^a, B^**  (0.2) |  | **1.6^a, B^**  (0.2) |  | **2.6^a, B^**  (0.4) |  | **39** |  | **61** |
|  | **Low** pedogenic Fe oxides | 5‒10 |  |  | **0.9^b, B^**  (0.4) |  | **1.3^b, B^**  (0.5) |  | **2.2^b, B^**  (0.8) |  | **41** |  | **59** |
|  |  |  |  |  |  |  |  |  |  |  |  |  |  |
| **Forest** | **Low** aluminous clay‒ | 0‒5 |  |  | **1.4^a, A^**  (0.7) |  | **2.1^a, A^**  (1.1) |  | **3.5^a, A^**  (1.7) |  | **40** |  | **60** |
|  | **High** pedogenic Fe oxides | 5‒10 |  |  | **1.3^b, A^**  (0.6) |  | **1.9^ab, A^**  (0.8) |  | **3.2^ab, A^**  (1.4) |  | **41** |  | **59** |
|  |  |  |  |  |  |  |  |  |  |  |  |  |  |
| **Forest** | **High** aluminous clay‒ | 0‒5 |  |  | **2.2^a^**  (1.1) |  | **3.0^a^**  (1.8) |  | **5.2^a^**  (2.9) |  | **42** |  | **58** |
|  | **Low** pedogenic Fe oxides | 5‒10 |  |  | **2.3^a^**  (0.9) |  | **2.6^a^**  (0.8) |  | **4.9^a^**  (1.6) |  | **47** |  | **53** |
|  |  |  |  |  |  |  |  |  |  |  |  |  |  |
| **Forest** | **High** aluminous clay‒ | 0‒5 |  |  | **1.2^a, B^**  (0.7) |  | **2.0^a, A^**  (1.2) |  | **3.2^a, A^**  (1.9) |  | **38** |  | **62** |
|  | **High** pedogenic Fe oxides | 5‒10 |  |  | **1.4^b, B^**  (0.3) |  | **2.0^ab, B^**  0.5) |  | **3.4^ab, B^**  (0.8) |  | **41** |  | **59** |
|  |  |  |  |  |  |  |  |  |  |  |  |  |  |
| **Cropland** | **Low** aluminous clay‒ | 0‒5 |  |  | **3.9^a, A^**  (0.2) |  | **3.4^a, A^**  (0.2) |  | **7.3^a, A^**  (0.4) |  | **53** |  | **47** |
|  | **Low** pedogenic Fe oxides | 5‒10 |  |  | **3.5^a, A^**  (0.4) |  | **2.9^a, A^**  (0.3) |  | **6.4^a, A^**  (0.6) |  | **55** |  | **45** |
|  |  |  |  |  |  |  |  |  |  |  |  |  |  |
| **Cropland** | **Low** aluminous clay‒ | 0‒5 |  |  | **1.4^c, A^**  (0.2) |  | **1.6^c, A^**  (0.0) |  | **3.0^c, A^**  (0.2) |  | **47** |  | **53** |
|  | **High** pedogenic Fe oxides | 5‒10 |  |  | **1.2^b, A^**  (0.2) |  | **1.5^b, A^**  (0.2) |  | **2.8^b, A^**  (0.4) |  | **43** |  | **57** |
|  |  |  |  |  |  |  |  |  |  |  |  |  |  |
| **Cropland** | **High** aluminous clay‒ | 0‒5 |  |  | **2.8^b, A^**  (0.3) |  | **2.6^b, A^**  (0.4) |  | **5.3^b, A^**  (0.7) |  | **53** |  | **47** |
|  | **High** pedogenic Fe oxides | 5‒10 |  |  | **3.5^a, A^**  (0.4) |  | **2.9^a, A^**  (0.4) |  | **6.4^a, A^**  (0.8) |  | **55** |  | **45** |
|  |  |  |  |  |  |  |  |  |  |  |  |  |  |

# Section 9: Figures


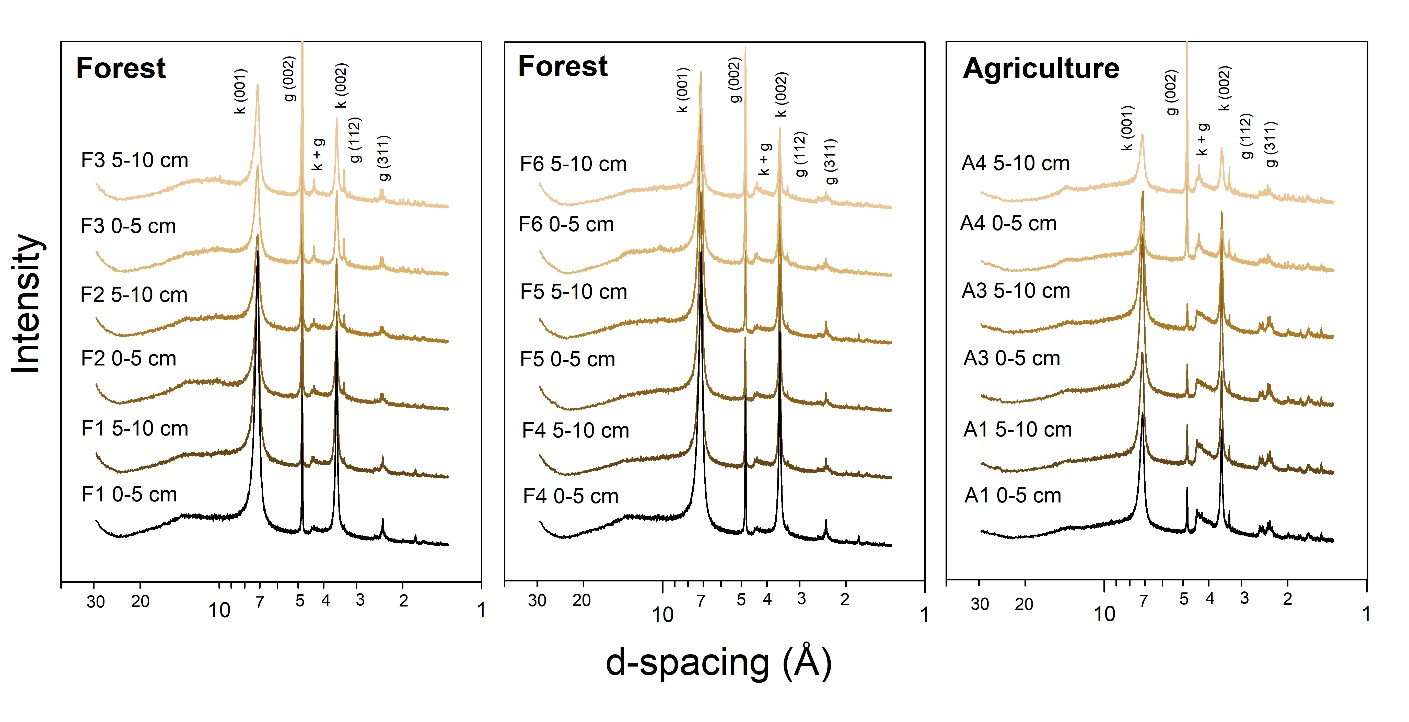


**Figure S1:** X-ray diffractograms of oriented magnesium- and ethylene glycol-saturated aluminous clay samples (CuKα) from soils along the mineralogical combinations as separated by depth and land-use. Prior analysis, OM and pedogenic Fe (oxyhydr)oxides have been removed by treatment with 30% H_2_O_2_ and dithionite-citrate-bicarbonate, respectively. Peaks are labeled k for kaolinite and g for gibbsite, and the respective *hkl* indices are in given in parentheses. For sample codes, please refer to Table S1.

**Figure S2:** Measured and model-fitted MB spectra of the mineralogical combinations. The y-axes are count intensities (arbitrary units), and the x-axes are source velocities (mm s^‒1^). Black dots represent measured values, and the colored lines represent the modeled phases as indicated in the legend. The headings of each figure indicate the mineralogical combination and the temperature in Kelvin (K). Together with the three spectra of each mineralogical combination and depth, the relevant spectra data provided in an associated table.

**‛low clay‒low Fe’ Forest (0‒5 cm) 295 K**

**‛low clay‒low Fe’ Forest (0‒5 cm) 77 K**

**‛low clay‒low Fe’ Forest (0‒5 cm) 5 K**

| **Sample** |  |  | **Phase** |  | **Area** |  | $\bar{\text{CS}}$ **or δ_0_** | $\bar{\text{ε}}$ |  | **P** |  | **Δ or H** | **σ** | $\bar{\text{QS}}$ **or** $\bar{\text{H}}$ | $\text{Χ}_{\text{ν}}^{\text{2}}$ |
| --- | --- | --- | --- | --- | --- | --- | --- | --- | --- | --- | --- | --- | --- | --- | --- |
| **‛low clay‒low Fe’ Forest**  **(0‒5 cm)** |  |  |  |  | (%) |  | (mm s^‒1^) | |  | (%) |  | (mm s^‒1^) | | |  |
|  |  |  |  |  |  |  |  |  |  |  |  |  |  |  |  |
|  |  |  |  |  |  |  |  |  |  |  |  |  |  |  |  |
| **295 K** |  |  | **ferric 1** |  | **80.8**  (24) |  | 0.3599  (19) | n/a |  | 56.2563* |  | 0.488  (28) | 0.05  (10) | 0.6 | 0.62 |
|  |  |  |  |  |  |  |  |  |  | 44  (53) |  | 0.73  (26) | 0.18  (11) |  |  |
|  |  |  | **ferrous 1** |  | **0.36**  (63) |  | 1.00  (37) | n/a |  | 100* |  | 0.91  (74) | 0.0004 | 0.91 |  |
|  |  |  | **OX1** |  | **6.8**  (12) |  | 0.411  (48) | -0.073  (48) |  | 100* |  | 49.48  (41) | 1.51  (53) | 49.48 |  |
|  |  |  |  |  |  |  |  |  |  |  |  |  |  |  |  |
| (BG = 3.8 MC/ch) |  |  | **(b)OxHy** |  | **12.0**  (23) |  | 0.5 | 0* |  | 100* |  | 0* | 42  (17) | 33.43 |  |
|  |  |  |  |  |  |  |  |  |  |  |  |  |  |  |  |
|  |  |  |  |  |  |  |  |  |  |  |  |  |  |  |  |
| **77 K** |  |  | **ferric 1** |  | **3.88**  (62) |  | 0.449  (24) | n/a |  | 100* |  | 0.695  (44) | 0.15* | 0.7 | 0.66 |
|  |  |  | **ferrous 1** |  | **0.09**  (46) |  | 1.2* | n/a |  | 100* |  | 1* | 0.0891625 | 1 |  |
|  |  |  | **OX1** |  | **7.1**  (17) |  | 0.462  (20) | -0.077  (20) |  | 100* |  | 52.53  (17) | 0.57  (32) | 52.53 |  |
|  |  |  | **OxHy1** |  | **64.2**  (40) |  | 0.476  (11) | -0.130  (11) |  | 49.8114* |  | 45.66  (19) | 2.64  (49) | 42.77 |  |
|  |  |  |  |  |  |  |  |  |  | 50  (27) |  | 39.9  (35) | 5.8  (19) |  |  |
| (BG = 3.8 MC/ch) |  |  | **(b)OxHy** |  | **24.7**  (41) |  | 0.5* | 0* |  | 100* |  | 0* | 25.5  (70) | 20.32 |  |
|  |  |  |  |  |  |  |  |  |  |  |  |  |  |  |  |
|  |  |  |  |  |  |  |  |  |  |  |  |  |  |  |  |
| **5 K** |  |  | **ferric 1** |  | **1.60**  (52) |  | 0.463  (68) | n/a |  | 100* |  | 0.70  (12) | 0.2* | 0.7 | 0.85 |
|  |  |  | **ferrous 1** |  | **0.87**  (46) |  | 0.902  (76) | n/a |  | 100* |  | 1.17  (15) | 1.797e^-05^ | 1.17 |  |
|  |  |  | **OX1** |  | **10.4**  (43) |  | 0.482  (12) | -0.078  (17) |  | 100* |  | 52.99  (14) | 0.51  (31) | 52.99 |  |
|  |  |  | **OxHy1** |  | **77.5**  (43) |  | 0.4748  (33) | -0.1169  (33) |  | 61.4363 |  | 49.152  (98) | 1.07  (23) | 48.43 |  |
|  |  |  |  |  |  |  |  |  |  | 39  (32) |  | 47.3  (24) | 2.57  (91) |  |  |
| (BG = 3.8 MC/ch) |  |  | **(b)OxHy** |  | **9.7**  (22) |  | 0.5* | 0* |  | 100* |  | 0* | 39  (14) | 31.45 |  |
|  |  |  |  |  |  |  |  |  |  |  |  |  |  |  |  |

BG = background level, in mega-counts per channel (MC/ch).

Phase = assigned spectral component of a certain mineral phase (ferrous1 = ferrous content of the sample; Ox1 = hematite; OxHy1 = goethite).

$\bar{\text{CS}}$ or δ_0_= the center shift of a Gaussian component in the quadrupole splitting distribution (QSD) of the hyperfine field distribution (HFD) of a given spectral component, given in mm s^‒1^.

$\bar{\text{QS}}$ = the center shift of a Gaussian component in the quadrupole splitting distribution (QSD) of the hyperfine field distribution (HFD) of a given spectral component, given in mm s^‒1^.

σ = the Gaussian standard deviation width of a given Gaussian component of a given QSD or HFD.

P = the weight factor (%) for a given Gaussian component in a given QSD or HFD.

H and $\bar{\text{H}}$ = the average magnitude of the hyperfine field (expressed as an excited state Zeeman splitting, in Torr (T), in a given HFD of a given sextet spectral component, or all components, respectively.

Δ = the average magnitude of the quadrupole splitting in a given QSD of a given doublet spectral component, given in mm s^‒1^.

$\bar{\text{ε}}$ = the average magnitude of the slave distribution of quadrupole shifts ($\text{ε}$) associated to a given HFD of a given sextet spectral component, given in mm s^‒1^.

$\text{Χ}_{\text{ν}}^{\text{2}}$ = the reduced chi-squared value for the fit: chi-squared ($\text{Χ}$^2^) divided by the number of degrees of freedom ($\text{ν}$). It has an ideal value of 1 for a correct model.

All fits performed using the Voigt-based fitting method of Rancourt and Ping ^3^ with the Recoil^TM^ software.

All fitting and calculated parameters are as defined in by Rancourt and Ping ^3^.

All errors are shown in parentheses in concise notation as the error on the last digit (e.g, 5.13 ± 0.25 is 5.13 (25))

All δ-1 couplings between CS and H (or DELTA) were taken to be zero.

All line-1 to line-2 area ratios in all (distributed) elemental doublets were taken to be 1.

All line-2/line-3 and line-1/line-3 area ratios in all (distributed and symmetric) elemental sextets were taken to be 2 and 3, respectively.

All ε-1 couplings between epsilon and H (in a HFD) are taken to be 0.

All Lorentzian half widths at half maximum (HWHM) are set at 0.1425 mm s^‒1^ as measured on Fe foil standards on the instrument.

All center shifts ($\bar{\text{CS}}$ or δ_0_) are given with respect to the CS of metallic Fe at 295 K.

**‛low clay‒low Fe’ Forest (5‒10 cm) 295 K**

**‛low clay‒low Fe’ Forest (5‒10 cm) 77 K**

**‛low clay‒low Fe’ Forest (5‒10 cm) 5 K**

| **Sample** |  |  | **Phase** |  | **Area** |  | $\bar{\text{CS}}$ **or δ_0_** | $\bar{\text{ε}}$ |  | **P** |  | **Δ or H** | **σ** | $\bar{\text{QS}}$ **or** $\bar{\text{H}}$ | $\text{Χ}_{\text{ν}}^{\text{2}}$ |
| --- | --- | --- | --- | --- | --- | --- | --- | --- | --- | --- | --- | --- | --- | --- | --- |
| **‛low clay‒low Fe’ Forest**  **(5‒10 cm)** |  |  |  |  | (%) |  | (mm s^‒1^) | |  | (%) |  | (mm s^‒1^) | | |  |
|  |  |  |  |  |  |  |  |  |  |  |  |  |  |  |  |
|  |  |  |  |  |  |  |  |  |  |  |  |  |  |  |  |
| **295 K** |  |  | **ferric1** |  | **74.4**  (14) |  | 0.3591  (22) | n/a |  | 62.7556* |  | 0.500  (24) | 0.084  (49) | 0.6 | 0.83 |
|  |  |  |  |  |  |  |  |  |  | 37  (39) |  | 0.78  (25) | 0.20  (11) |  |  |
|  |  |  | **ferrous1** |  | **0.81**  (33) |  | 0.89  (14) | n/a |  | 100* |  | 0.58  (30) | 0.000392 | 0.58 |  |
|  |  |  | **OX1** |  | **8.68**  (86) |  | 0.378  (32) | -0.110  (32) |  | 100* |  | 48.62  (28) | 2.29  (36) | 48.62 |  |
|  |  |  |  |  |  |  |  |  |  |  |  |  |  |  |  |
| (BG = 3.8 MC/ch) |  |  | **(b)OxHy** |  | **16.1**  (14) |  | 0.3* | 0* |  | 100* |  | 0* | 40.1  (69) | 31.98 |  |
|  |  |  |  |  |  |  |  |  |  |  |  |  |  |  |  |
|  |  |  |  |  |  |  |  |  |  |  |  |  |  |  |  |
| **77 K** |  |  | **ferric1** |  | **3.68**  (32) |  | 0.475  (11) | n/a |  | 100* |  | 0.699  (20) | 0.15* | 0.7 | 1.77 |
|  |  |  | **ferrous1** |  | **0.23**  (24) |  | 1.08305* | n/a |  | 100* |  | 1.208* | 0.08916* | 1.21 |  |
|  |  |  | **OX1** |  | **3.12**  (48) |  | 0.477  (15) | -0.103  (15) |  | 100* |  | 52.75  (11) | 0  (86) | 52.75 |  |
|  |  |  | **OxHy1** |  | **74.4**  (26) |  | 0.4728  (48) | -0.1346  (47) |  | 54.1993* |  | 46.266  (77) | 3.20  (13) | 42.96 |  |
|  |  |  |  |  |  |  |  |  |  | 45.8  (60) |  | 39.05  (89) | 7.45  (70) |  |  |
| (BG = 3.8 MC/ch) |  |  | **(b)OxHy** |  | **18.6**  (27) |  | 0.5* | 0* |  | 100* |  | 0* | 23.3  (56) | 18.6 |  |
|  |  |  |  |  |  |  |  |  |  |  |  |  |  |  |  |
|  |  |  |  |  |  |  |  |  |  |  |  |  |  |  |  |
| **5 K** |  |  | **ferric1** |  | **1.27**  (27) |  | 0.423  (45) | n/a |  | 100* |  | 0.655  (84) | 0.2* | 0.66 | 1.5 |
|  |  |  | **ferrous1** |  | **0.46**  (24) |  | 0.849  (77) | n/a |  | 100* |  | 1.18  (15) | 1.797e^-05^ | 1.19 |  |
|  |  |  | **OX1** |  | **9.3**  (22) |  | 0.4821  (73) | -0.0826  (89) |  | 100* |  | 53.003  (75) | 0.51  (19) | 53 |  |
|  |  |  | **OxHy1** |  | **79.5**  (23) |  | 0.4757  (17) | -0.1160  (17) |  | 61.2429* |  | 49.182  (41) | 1.052  (86) | 48.44 |  |
|  |  |  |  |  |  |  |  |  |  | 39  (11) |  | 47.26  (97) | 2.84  (40) |  |  |
| (BG = 3.8 MC/ch) |  |  | **(b)OxHy** |  | **9.5**  (12) |  | 0.5* | 0* |  | 100* |  | 0* | 39.6  (79) | 31.6 |  |
|  |  |  |  |  |  |  |  |  |  |  |  |  |  |  |  |

BG = background level, in mega-counts per channel (MC/ch).

Phase = assigned spectral component of a certain mineral phase (ferrous1 = ferrous content of the sample; Ox1 = hematite; OxHy1 = goethite).

$\bar{\text{CS}}$ or δ_0_= the center shift of a Gaussian component in the quadrupole splitting distribution (QSD) of the hyperfine field distribution (HFD) of a given spectral component, given in mm s^‒1^.

$\bar{\text{QS}}$ = the center shift of a Gaussian component in the quadrupole splitting distribution (QSD) of the hyperfine field distribution (HFD) of a given spectral component, given in mm s^‒1^.

σ = the Gaussian standard deviation width of a given Gaussian component of a given QSD or HFD.

P = the weight factor (%) for a given Gaussian component in a given QSD or HFD.

H and $\bar{\text{H}}$ = the average magnitude of the hyperfine field (expressed as an excited state Zeeman splitting, in Torr (T), in a given HFD of a given sextet spectral component, or all components, respectively.

Δ = the average magnitude of the quadrupole splitting in a given QSD of a given doublet spectral component, given in mm s^‒1^.

$\bar{\text{ε}}$ = the average magnitude of the slave distribution of quadrupole shifts ($\text{ε}$) associated to a given HFD of a given sextet spectral component, given in mm s^‒1^.

$\text{Χ}_{\text{ν}}^{\text{2}}$ = the reduced chi-squared value for the fit: chi-squared ($\text{Χ}$^2^) divided by the number of degrees of freedom ($\text{ν}$). It has an ideal value of 1 for a correct model.

All fits performed using the Voigt-based fitting method of Rancourt and Ping ^3^ with the Recoil^TM^ software.

All fitting and calculated parameters are as defined in by Rancourt and Ping ^3^.

All errors are shown in parentheses in concise notation as the error on the last digit (e.g, 5.13 ± 0.25 is 5.13 (25))

All δ-1 couplings between CS and H (or DELTA) were taken to be zero.

All line-1 to line-2 area ratios in all (distributed) elemental doublets were taken to be 1.

All line-2/line-3 and line-1/line-3 area ratios in all (distributed and symmetric) elemental sextets were taken to be 2 and 3, respectively.

All ε-1 couplings between epsilon and H (in a HFD) are taken to be 0.

All Lorentzian half widths at half maximum (HWHM) are set at 0.1425 mm s^‒1^ as measured on Fe foil standards on the instrument.

All center shifts ($\bar{\text{CS}}$ or δ_0_) are given with respect to the CS of metallic Fe at 295 K.

**‛low clay‒high Fe’ Forest (0‒5 cm) 295 K**

**‛low clay‒high Fe’ Forest (0‒5 cm) 77 K**

**‛low clay‒high Fe’ Forest (0‒5 cm) 5 K**

| **Sample** |  |  | **Phase** |  | **Area** |  | $\bar{\text{CS}}$ **or δ_0_** | $\bar{\text{ε}}$ |  | **P** |  | **Δ or H** | **σ** | $\bar{\text{QS}}$ **or** $\bar{\text{H}}$ | $\text{Χ}_{\text{ν}}^{\text{2}}$ |
| --- | --- | --- | --- | --- | --- | --- | --- | --- | --- | --- | --- | --- | --- | --- | --- |
| **‛low clay‒high Fe’ Forest**  **(0‒5 cm)** |  |  |  |  | (%) |  | (mm s^‒1^) | |  | (%) |  | (mm s^‒1^) | | |  |
|  |  |  |  |  |  |  |  |  |  |  |  |  |  |  |  |
|  |  |  |  |  |  |  |  |  |  |  |  |  |  |  |  |
| **295 K** |  |  | **ferric1** |  | **74.1**  (10) |  | 0.3574  (12) | n/a |  | 58.3957* |  | 0.489  (13) | 0.087  (35) | 0.6 | 0.95 |
|  |  |  |  |  |  |  |  |  |  | 42  (24) |  | 0.76  (14) | 0.215  (57) |  |  |
|  |  |  | **ferrous1** |  | **1.04**  (25) |  | 1.004  (56) | n/a |  | 100* |  | 0.61  (11) | 0.00039* | 0.61 |  |
|  |  |  | **OX1** |  | **8.24**  (57) |  | 0.379  (21) | -0.105  (21) |  | 100* |  | 48.86  (18) | 1.95  (23) | 48.86 |  |
|  |  |  |  |  |  |  |  |  |  |  |  |  |  |  |  |
| (BG = 3.8 MC/ch) |  |  | **(b)OxHy** |  | **16.64**  (99) |  | **0.5*** | 0* |  | 100* |  | 0* | 41.2  (51) | 32.89 |  |
|  |  |  |  |  |  |  |  |  |  |  |  |  |  |  |  |
|  |  |  |  |  |  |  |  |  |  |  |  |  |  |  |  |
| **77 K** |  |  | **ferric1** |  | **2.41**  (40) |  | 0.480  (19) | n/a |  | 100* |  | 0.749  (38) | 0.15* | 0.75 | 1.15 |
|  |  |  | **ferrous1** |  | **0.44**  (25) |  | 1.28  (10) | n/a |  | 100* |  | 1.28  (18) | 0.08916* | 1.28 |  |
|  |  |  | **OX1** |  | **5.10**  (60) |  | 0.469  (11) | -0.081  (11) |  | 100* |  | 52.640  (80) | 0.12  (44) | 52.64 |  |
|  |  |  | **OxHy1** |  | **71.5**  (35) |  | 0.4685  (57) | -0.1250  (54) |  | 49.8397* |  | 46.310  (82) | 2.85  (18) | 42.78 |  |
|  |  |  |  |  |  |  |  |  |  | 50.2  (87) |  | 39.3  (12) | 6.67  (83) |  |  |
| (BG = 3.8 MC/ch) |  |  | **(b)OxHy** |  | **20.6**  37) |  | 0.5* | 0* |  | 100* |  | 0* | 24.7  (72) | 19.71 |  |
|  |  |  |  |  |  |  |  |  |  |  |  |  |  |  |  |
|  |  |  |  |  |  |  |  |  |  |  |  |  |  |  |  |
| **5 K** |  |  | **ferric1** |  | **0.92**  (19) |  | 0.439  (43) | n/a |  | 100* |  | 0.688  (80) | 0.2* | 0.69 | 2.04 |
|  |  |  | **ferrous1** |  | **0.61**  (17) |  | 0.869  (40) | n/a |  | 100* |  | 1.227  (78) | 1.797e^-05^* | 1.23 |  |
|  |  |  | **OX1** |  | **9.1**  (16) |  | 0.4760  (48) | -0.0912  (54) |  | 100* |  | 53.030  (49) | 0.39  (14) | 53.03 |  |
|  |  |  | **OxHy1** |  | **82.1**  (16) |  | 0.4745  (11) | -0.1140  (11) |  | 57.5858* |  | 49.252  (35) | 1.036  (75) | 48.5 |  |
|  |  |  |  |  |  |  |  |  |  | 42.4  (95) |  | 47.47  (66) | 2.60  (25) |  |  |
| (BG = 3.8 MC/ch) |  |  | **(b)OxHy** |  | **7.26**  (81) |  | 0.5* | 0* |  | 100* |  | 0* | 39.5  (70) | 31.55 |  |
|  |  |  |  |  |  |  |  |  |  |  |  |  |  |  |  |

BG = background level, in mega-counts per channel (MC/ch).

Phase = assigned spectral component of a certain mineral phase (ferrous1 = ferrous content of the sample; Ox1 = hematite; OxHy1 = goethite).

$\bar{\text{CS}}$ or δ_0_= the center shift of a Gaussian component in the quadrupole splitting distribution (QSD) of the hyperfine field distribution (HFD) of a given spectral component, given in mm s^‒1^.

$\bar{\text{QS}}$ = the center shift of a Gaussian component in the quadrupole splitting distribution (QSD) of the hyperfine field distribution (HFD) of a given spectral component, given in mm s^‒1^.

σ = the Gaussian standard deviation width of a given Gaussian component of a given QSD or HFD.

P = the weight factor (%) for a given Gaussian component in a given QSD or HFD.

H and $\bar{\text{H}}$ = the average magnitude of the hyperfine field (expressed as an excited state Zeeman splitting, in Torr (T), in a given HFD of a given sextet spectral component, or all components, respectively.

Δ = the average magnitude of the quadrupole splitting in a given QSD of a given doublet spectral component, given in mm s^‒1^.

$\bar{\text{ε}}$ = the average magnitude of the slave distribution of quadrupole shifts ($\text{ε}$) associated to a given HFD of a given sextet spectral component, given in mm s^‒1^.

$\text{Χ}_{\text{ν}}^{\text{2}}$ = the reduced chi-squared value for the fit: chi-squared ($\text{Χ}$^2^) divided by the number of degrees of freedom ($\text{ν}$). It has an ideal value of 1 for a correct model.

All fits performed using the Voigt-based fitting method of Rancourt and Ping ^3^ with the Recoil^TM^ software.

All fitting and calculated parameters are as defined in by Rancourt and Ping ^3^.

All errors are shown in parentheses in concise notation as the error on the last digit (e.g, 5.13 ± 0.25 is 5.13 (25))

All δ-1 couplings between CS and H (or DELTA) were taken to be zero.

All line-1 to line-2 area ratios in all (distributed) elemental doublets were taken to be 1.

All line-2/line-3 and line-1/line-3 area ratios in all (distributed and symmetric) elemental sextets were taken to be 2 and 3, respectively.

All ε-1 couplings between epsilon and H (in a HFD) are taken to be 0.

All Lorentzian half widths at half maximum (HWHM) are set at 0.1425 mm s^‒1^ as measured on Fe foil standards on the instrument.

All center shifts ($\bar{\text{CS}}$ or δ_0_) are given with respect to the CS of metallic Fe at 295 K.

**‛low clay‒high Fe’ Forest (5‒10 cm) 295 K**

**‛low clay‒high Fe’ Forest (5‒10 cm) 77 K**

**‛low clay‒high Fe’ Forest (5‒10 cm) 5 K**

| **Sample** |  |  | **Phase** |  | **Area** |  | $\bar{\text{CS}}$ **or δ_0_** | $\bar{\text{ε}}$ |  | **P** |  | **Δ or H** | **σ** | $\bar{\text{QS}}$ **or** $\bar{\text{H}}$ | $\text{Χ}_{\text{ν}}^{\text{2}}$ |
| --- | --- | --- | --- | --- | --- | --- | --- | --- | --- | --- | --- | --- | --- | --- | --- |
| **‛low clay‒high Fe’ Forest**  **(5‒10 cm)** |  |  |  |  | (%) |  | (mm s^‒1^) | |  | (%) |  | (mm s^‒1^) | | |  |
|  |  |  |  |  |  |  |  |  |  |  |  |  |  |  |  |
|  |  |  |  |  |  |  |  |  |  |  |  |  |  |  |  |
| **295 K** |  |  | **ferric1** |  | **76.54**  (71) |  | 0.35898  (59) | n/a |  | 61.1743* |  | 0.500  (11) | 0.100  (24) | 0.61 | 1.29 |
|  |  |  |  |  |  |  |  |  |  | 39  (19) |  | 0.79  (12) | 0.221  (50) |  |  |
|  |  |  | **ferrous1** |  | **1.22**  (17) |  | 0.940  (30) | n/a |  | 100* |  | 0.817  (59) | 0.00039* | 0.82 |  |
|  |  |  | **OX1** |  | **7.95**  (41) |  | 0.388  (16) | -0.115  (16) |  | 100* |  | 49.05  (14) | 2.08  (18) | 49.05 |  |
|  |  |  |  |  |  |  |  |  |  |  |  |  |  |  |  |
| (BG = 3.8 MC/ch) |  |  | **(b)OxHy** |  | **14.29**  (68) |  | 0.3* | 0* |  | 100* |  | 0* | 38.5  (36) | 30.7 |  |
|  |  |  |  |  |  |  |  |  |  |  |  |  |  |  |  |
|  |  |  |  |  |  |  |  |  |  |  |  |  |  |  |  |
| **77 K** |  |  | **ferric1** |  | **2.50**  (21) |  | 0.474  (13) | n/a |  | 100* |  | 0.707  (25) | 0.15* | 0.71 | 1.58 |
|  |  |  | **ferrous1** |  | **0.00**  (18) |  | 1.08305* | n/a |  | 100* |  | 1.208* | 0.08916* | 1.21 |  |
|  |  |  | **OX1** |  | **4.09**  (40) |  | 0.4745  (99) | -0.0868  (100) |  | 100* |  | 52.678  (77) | 0.25  (22) | 52.68 |  |
|  |  |  | **OxHy1** |  | **67.9**  (17) |  | 0.4736  (45) | -0.1246  (44) |  | 52.9479* |  | 45.939  (69) | 3.18  (17) | 42.45 |  |
|  |  |  |  |  |  |  |  |  |  | 47.1  (82) |  | 38.5  (13) | 6.89  (78) |  |  |
| (BG = 3.8 MC/ch) |  |  | **(b)OxHy** |  | **25.5**  (18) |  | 0.5* | 0* |  | 100* |  | 0* | 27.0  (29) | 21.51 |  |
|  |  |  |  |  |  |  |  |  |  |  |  |  |  |  |  |
|  |  |  |  |  |  |  |  |  |  |  |  |  |  |  |  |
| **5 K** |  |  | **ferric1** |  | **0.88**  (25) |  | 0.470  (62) | n/a |  | 100* |  | 0.67  (11) | 0.2* | 0.67 | 1.53 |
|  |  |  | **ferrous1** |  | **0.52**  (23) |  | 0.908  (63) | n/a |  | 100* |  | 1.15  (12) | 1.797e^-05*^ | 1.15 |  |
|  |  |  | **OX1** |  | **7.1**  (15) |  | 0.4776  (79) | -0.0894  (82) |  | 100* |  | 53.068  (67) | 0.30  (23) | 53.07 |  |
|  |  |  | **OxHy1** |  | **84.2**  (17) |  | 0.4740  (16) | -0.1104  (16) |  | 55.5927* |  | 49.173  (39) | 1.100  (69) | 48.51 |  |
|  |  |  |  |  |  |  |  |  |  | 44.4  (70) |  | 47.67  (49) | 2.91  (19) |  |  |
| (BG = 3.8 MC/ch) |  |  | **(b)OxHy** |  | **7.3**  (11) |  | 0.5* | 0* |  | 100* |  | 0* | 41  (10) | 33.05 |  |
|  |  |  |  |  |  |  |  |  |  |  |  |  |  |  |  |

BG = background level, in mega-counts per channel (MC/ch).

Phase = assigned spectral component of a certain mineral phase (ferrous1 = ferrous content of the sample; Ox1 = hematite; OxHy1 = goethite).

$\bar{\text{CS}}$ or δ_0_= the center shift of a Gaussian component in the quadrupole splitting distribution (QSD) of the hyperfine field distribution (HFD) of a given spectral component, given in mm s^‒1^.

$\bar{\text{QS}}$ = the center shift of a Gaussian component in the quadrupole splitting distribution (QSD) of the hyperfine field distribution (HFD) of a given spectral component, given in mm s^‒1^.

σ = the Gaussian standard deviation width of a given Gaussian component of a given QSD or HFD.

P = the weight factor (%) for a given Gaussian component in a given QSD or HFD.

H and $\bar{\text{H}}$ = the average magnitude of the hyperfine field (expressed as an excited state Zeeman splitting, in Torr (T), in a given HFD of a given sextet spectral component, or all components, respectively.

Δ = the average magnitude of the quadrupole splitting in a given QSD of a given doublet spectral component, given in mm s^‒1^.

$\bar{\text{ε}}$ = the average magnitude of the slave distribution of quadrupole shifts ($\text{ε}$) associated to a given HFD of a given sextet spectral component, given in mm s^‒1^.

$\text{Χ}_{\text{ν}}^{\text{2}}$ = the reduced chi-squared value for the fit: chi-squared ($\text{Χ}$^2^) divided by the number of degrees of freedom ($\text{ν}$). It has an ideal value of 1 for a correct model.

All fits performed using the Voigt-based fitting method of Rancourt and Ping ^3^ with the Recoil^TM^ software.

All fitting and calculated parameters are as defined in by Rancourt and Ping ^3^.

All errors are shown in parentheses in concise notation as the error on the last digit (e.g, 5.13 ± 0.25 is 5.13 (25))

All δ-1 couplings between CS and H (or DELTA) were taken to be zero.

All line-1 to line-2 area ratios in all (distributed) elemental doublets were taken to be 1.

All line-2/line-3 and line-1/line-3 area ratios in all (distributed and symmetric) elemental sextets were taken to be 2 and 3, respectively.

All ε-1 couplings between epsilon and H (in a HFD) are taken to be 0.

All Lorentzian half widths at half maximum (HWHM) are set at 0.1425 mm s^‒1^ as measured on Fe foil standards on the instrument.

All center shifts ($\bar{\text{CS}}$ or δ_0_) are given with respect to the CS of metallic Fe at 295 K.

**‛high clay‒low Fe’ Forest (0‒5 cm) 295 K**

**‛high clay‒low Fe’ Forest (0‒5 cm) 77 K**

**‛high clay‒low Fe’ Forest (0‒5 cm) 5 K**

| **Sample** |  |  | **Phase** |  | **Area** |  | $\bar{\text{CS}}$ **or δ_0_** | $\bar{\text{ε}}$ |  | **P** |  | **Δ or H** | **σ** | $\bar{\text{QS}}$ **or** $\bar{\text{H}}$ | $\text{Χ}_{\text{ν}}^{\text{2}}$ |
| --- | --- | --- | --- | --- | --- | --- | --- | --- | --- | --- | --- | --- | --- | --- | --- |
| **‛high clay‒low Fe’ Forest**  **(0‒5 cm)** |  |  |  |  | (%) |  | (mm s^‒1^) | |  | (%) |  | (mm s^‒1^) | | |  |
|  |  |  |  |  |  |  |  |  |  |  |  |  |  |  |  |
|  |  |  |  |  |  |  |  |  |  |  |  |  |  |  |  |
| **295 K** |  |  | **ferric1** |  | **72.2**  (10) |  | 0.3580  (16) | n/a |  | 60.2371* |  | 0.492  (15) | 0.050  (50) | 0.6 | 1.01 |
|  |  |  |  |  |  |  |  |  |  | 40  (26) |  | 0.76  (15) | 0.181  (72) |  |  |
|  |  |  | **ferrous1** |  | **1.61**  (29) |  | 1.014  (50) | n/a |  | 100* |  | 0.67  (10) | 0.001* | 0.67 |  |
|  |  |  | **OX1** |  | **11.6**  (53) |  | 0.382  (11) | -0,091  (11) |  | 100* |  | 50.855  (92) | 1.09  (13) | 50.85 |  |
|  |  |  |  |  |  |  |  |  |  |  |  |  |  |  |  |
| (BG = 3.8 MC/ch) |  |  | **(b)OxHy** |  | **14.6**  (11) |  | 0.5* | 0* |  | 100* |  | 0* | 41.5  (67) | 33.11 |  |
|  |  |  |  |  |  |  |  |  |  |  |  |  |  |  |  |
|  |  |  |  |  |  |  |  |  |  |  |  |  |  |  |  |
| **77 K** |  |  | **ferric1** |  | **5.16**  (45) |  | 0.4705  (89) | n/a |  | 100* |  | 0.655  (15) | 0.248  (25) | 0.66 | 1.53 |
|  |  |  | **ferrous1** |  | **0.21**  (17) |  | 1* | n/a |  | 100* |  | 0.8* | 0.001* | 0.8 |  |
|  |  |  | **OX1** |  | **9.94**  (68) |  | 0.4701  (47) | -0.0728  (48) |  | 100* |  | 52.779  (41) | 0.523  (75) | 52.78 |  |
|  |  |  | **OxHy1** |  | **60.4**  (28) |  | 0.4723  (51) | -0.1345  (50) |  | 51.2665* |  | 46.292  (84) | 3.14  (15) | 42.99 |  |
|  |  |  |  |  |  |  |  |  |  | 48.7  (70) |  | 39.5  (11) | 7.23  (67) |  |  |
| (BG = 3.8 MC/ch) |  |  | **(b)OxHy** |  | **24.3**  (33) |  | 0.5* | 0* |  | 100* |  | 0* | 27.7  (63) | 22.1 |  |
|  |  |  |  |  |  |  |  |  |  |  |  |  |  |  |  |
|  |  |  |  |  |  |  |  |  |  |  |  |  |  |  |  |
| **5 K** |  |  | **ferric1** |  | **2.12**  (33) |  | 0.486  (30) | n/a |  | 100* |  | 0.607  (55) | 0.2* | 0.61 | 1.53 |
|  |  |  | **ferrous1** |  | **0.40**  (26) |  | 1.091  (94) | n/a |  | 100* |  | 0.83  (20) | 0  (530) | 0.83 |  |
|  |  |  | **OX1** |  | **14.2**  (12) |  | 0.4816  (43) | -0.0805  (46) |  | 100* |  | 53.053  (37) | 0.35  (10) | 53.05 |  |
|  |  |  | **OxHy1** |  | **77.4**  (13) |  | 0.4744  (18) | -0.1126  (18) |  | 55.0134* |  | 49.150  (33) | 1.057  (62) | 48.57 |  |
|  |  |  |  |  |  |  |  |  |  | 45.0  (52) |  | 47.86  (39) | 3.18  (18) |  |  |
| (BG = 3.8 MC/ch) |  |  | **(b)OxHy** |  | **5.94**  (72) |  | 0.5* | 0* |  | 100* |  | 0* | 20.0  (47) | 15.96 |  |
|  |  |  |  |  |  |  |  |  |  |  |  |  |  |  |  |

BG = background level, in mega-counts per channel (MC/ch).

Phase = assigned spectral component of a certain mineral phase (ferrous1 = ferrous content of the sample; Ox1 = hematite; OxHy1 = goethite).

$\bar{\text{CS}}$ or δ_0_= the center shift of a Gaussian component in the quadrupole splitting distribution (QSD) of the hyperfine field distribution (HFD) of a given spectral component, given in mm s^‒1^.

$\bar{\text{QS}}$ = the center shift of a Gaussian component in the quadrupole splitting distribution (QSD) of the hyperfine field distribution (HFD) of a given spectral component, given in mm s^‒1^.

σ = the Gaussian standard deviation width of a given Gaussian component of a given QSD or HFD.

P = the weight factor (%) for a given Gaussian component in a given QSD or HFD.

H and $\bar{\text{H}}$ = the average magnitude of the hyperfine field (expressed as an excited state Zeeman splitting, in Torr (T), in a given HFD of a given sextet spectral component, or all components, respectively.

Δ = the average magnitude of the quadrupole splitting in a given QSD of a given doublet spectral component, given in mm s^‒1^.

$\bar{\text{ε}}$ = the average magnitude of the slave distribution of quadrupole shifts ($\text{ε}$) associated to a given HFD of a given sextet spectral component, given in mm s^‒1^.

$\text{Χ}_{\text{ν}}^{\text{2}}$ = the reduced chi-squared value for the fit: chi-squared ($\text{Χ}$^2^) divided by the number of degrees of freedom ($\text{ν}$). It has an ideal value of 1 for a correct model.

All fits performed using the Voigt-based fitting method of Rancourt and Ping ^3^ with the Recoil^TM^ software.

All fitting and calculated parameters are as defined in by Rancourt and Ping ^3^.

All errors are shown in parentheses in concise notation as the error on the last digit (e.g, 5.13 ± 0.25 is 5.13 (25))

All δ-1 couplings between CS and H (or DELTA) were taken to be zero.

All line-1 to line-2 area ratios in all (distributed) elemental doublets were taken to be 1.

All line-2/line-3 and line-1/line-3 area ratios in all (distributed and symmetric) elemental sextets were taken to be 2 and 3, respectively.

All ε-1 couplings between epsilon and H (in a HFD) are taken to be 0.

All Lorentzian half widths at half maximum (HWHM) are set at 0.1425 mm s^‒1^ as measured on Fe foil standards on the instrument.

All center shifts ($\bar{\text{CS}}$ or δ_0_) are given with respect to the CS of metallic Fe at 295 K.

**‛high clay‒low Fe’ Forest (5‒10 cm) 295 K**

**‛high clay‒low Fe’ Forest (5‒10 cm) 77 K**

**‛high clay‒low Fe’ Forest (5‒10 cm) 5 K**

| **Sample** |  |  | **Phase** |  | **Area** |  | $\bar{\text{CS}}$ **or δ_0_** | $\bar{\text{ε}}$ |  | **P** |  | **Δ or H** | **σ** | $\bar{\text{QS}}$ **or** $\bar{\text{H}}$ | $\text{Χ}_{\text{ν}}^{\text{2}}$ |
| --- | --- | --- | --- | --- | --- | --- | --- | --- | --- | --- | --- | --- | --- | --- | --- |
| **‛high clay‒low Fe’ Forest**  **(5‒10 cm)** |  |  |  |  | (%) |  | (mm s^‒1^) | |  | (%) |  | (mm s^‒1^) | | |  |
|  |  |  |  |  |  |  |  |  |  |  |  |  |  |  |  |
|  |  |  |  |  |  |  |  |  |  |  |  |  |  |  |  |
| **295 K** |  |  | **ferric1** |  | **67.53**  (64) |  | 0.36116  (89) | n/a |  | 61.0114* |  | 0.501  (10) | 0.069  (30) | 0.61 | 1.62 |
|  |  |  |  |  |  |  |  |  |  | 39  (19) |  | 0.77  (12) | 0.198  (50) |  |  |
|  |  |  | **ferrous1** |  | **2.25**  (19) |  | 0.978  (21) | n/a |  | 100* |  | 0.827  (43) | 0.00039* | 0.83 |  |
|  |  |  | **OX1** |  | **14.49**  (35) |  | 0.3708  (58) | -0.1047  (58) |  | 100* |  | 50.801  (47) | 1.047  (66) | 50.8 |  |
|  |  |  |  |  |  |  |  |  |  |  |  |  |  |  |  |
| (BG = 3.8 MC/ch) |  |  | **(b)OxHy** |  | **15.73**  (72) |  | 0.3* | 0* |  | 100* |  | 0* | 41.8  (41) | 33.35 |  |
|  |  |  |  |  |  |  |  |  |  |  |  |  |  |  |  |
|  |  |  |  |  |  |  |  |  |  |  |  |  |  |  |  |
| **77 K** |  |  | **ferric1** |  | **4.89**  (50) |  | 0.472  (12) | n/a |  | 100* |  | 0.649  (22) | 0.231  (39) | 0.65 | 1.14 |
|  |  |  | **ferrous1** |  | **0.74**  (24) |  | 1.326  (48) | n/a |  | 100* |  | 1.432  (91) | 0.01* | 1.43 |  |
|  |  |  | **OX1** |  | **12.41**  (80) |  | 0.4819  (51) | -0.0707  (51) |  | 100* |  | 52.875  (45) | 0.578  (80) | 52.87 |  |
|  |  |  | **OxHy1** |  | **62.8**  (22) |  | 0.4771  (66) | -0.1241  (63) |  | 53.1066* |  | 46.34  (11) | 3.25  (20) | 42.85 |  |
|  |  |  |  |  |  |  |  |  |  | 46.9  (85) |  | 38.9  (13) | 7.13  (98) |  |  |
| (BG = 3.8 MC/ch) |  |  | **(b)OxHy** |  | **19.1**  (23) |  | 0.5* | 0* |  | 100* |  | 0* | 20.1  (44) | 16.02 |  |
|  |  |  |  |  |  |  |  |  |  |  |  |  |  |  |  |
|  |  |  |  |  |  |  |  |  |  |  |  |  |  |  |  |
| **5 K** |  |  | **ferric1** |  | **2.13**  (29) |  | 0.466  (29) | n/a |  | 100* |  | 0.652  (50) | 0.2* | 0.65 | 1.39 |
|  |  |  | **ferrous1** |  | **0.69**  (25) |  | 0.938  (52) | n/a |  | 100* |  | 1.095  (99) | 1.797e^-05^* | 1.09 |  |
|  |  |  | **OX1** |  | **15.0**  (15) |  | 0.4808  (42) | -0.0798  (46) |  | 100* |  | 53.076  (36) | 0.37  (11) | 53.08 |  |
|  |  |  | **OxHy1** |  | **74.0**  (17) |  | 0.4759  (20) | -0.1124  (20) |  | 48.3173* |  | 49.173  (42) | 0.976  (78) | 48.67 |  |
|  |  |  |  |  |  |  |  |  |  | 51.7  (61) |  | 48.20  (36) | 2.99  (18) |  |  |
| (BG = 3.8 MC/ch) |  |  | **(b)OxHy** |  | **8.2**  (12) |  | 0.5* | 0* |  | 100* |  | 0* | 32.7  (79) | 26.08 |  |
|  |  |  |  |  |  |  |  |  |  |  |  |  |  |  |  |

BG = background level, in mega-counts per channel (MC/ch).

Phase = assigned spectral component of a certain mineral phase (ferrous1 = ferrous content of the sample; Ox1 = hematite; OxHy1 = goethite).

$\bar{\text{CS}}$ or δ_0_= the center shift of a Gaussian component in the quadrupole splitting distribution (QSD) of the hyperfine field distribution (HFD) of a given spectral component, given in mm s^‒1^.

$\bar{\text{QS}}$ = the center shift of a Gaussian component in the quadrupole splitting distribution (QSD) of the hyperfine field distribution (HFD) of a given spectral component, given in mm s^‒1^.

σ = the Gaussian standard deviation width of a given Gaussian component of a given QSD or HFD.

P = the weight factor (%) for a given Gaussian component in a given QSD or HFD.

H and $\bar{\text{H}}$ = the average magnitude of the hyperfine field (expressed as an excited state Zeeman splitting, in Torr (T), in a given HFD of a given sextet spectral component, or all components, respectively.

Δ = the average magnitude of the quadrupole splitting in a given QSD of a given doublet spectral component, given in mm s^‒1^.

$\bar{\text{ε}}$ = the average magnitude of the slave distribution of quadrupole shifts ($\text{ε}$) associated to a given HFD of a given sextet spectral component, given in mm s^‒1^.

$\text{Χ}_{\text{ν}}^{\text{2}}$ = the reduced chi-squared value for the fit: chi-squared ($\text{Χ}$^2^) divided by the number of degrees of freedom ($\text{ν}$). It has an ideal value of 1 for a correct model.

All fits performed using the Voigt-based fitting method of Rancourt and Ping ^3^ with the Recoil^TM^ software.

All fitting and calculated parameters are as defined in by Rancourt and Ping ^3^.

All errors are shown in parentheses in concise notation as the error on the last digit (e.g, 5.13 ± 0.25 is 5.13 (25))

All δ-1 couplings between CS and H (or DELTA) were taken to be zero.

All line-1 to line-2 area ratios in all (distributed) elemental doublets were taken to be 1.

All line-2/line-3 and line-1/line-3 area ratios in all (distributed and symmetric) elemental sextets were taken to be 2 and 3, respectively.

All ε-1 couplings between epsilon and H (in a HFD) are taken to be 0.

All Lorentzian half widths at half maximum (HWHM) are set at 0.1425 mm s^‒1^ as measured on Fe foil standards on the instrument.

All center shifts ($\bar{\text{CS}}$ or δ_0_) are given with respect to the CS of metallic Fe at 295 K.

**‛high clay‒high Fe’ Forest (0‒5 cm) 295 K**

**‛high clay‒high Fe’ Forest (0‒5 cm) 77 K**

**‛high clay‒high Fe’ Forest (0‒5 cm) 5 K**

| **Sample** |  |  | **Phase** |  | **Area** |  | $\bar{\text{CS}}$ **or δ_0_** | $\bar{\text{ε}}$ |  | **P** |  | **Δ or H** | **σ** | $\bar{\text{QS}}$ **or** $\bar{\text{H}}$ | $\text{Χ}_{\text{ν}}^{\text{2}}$ |
| --- | --- | --- | --- | --- | --- | --- | --- | --- | --- | --- | --- | --- | --- | --- | --- |
| **‛high clay‒high Fe’ Forest**  **(0‒5 cm)** |  |  |  |  | (%) |  | (mm s^‒1^) | |  | (%) |  | (mm s^‒1^) | | |  |
|  |  |  |  |  |  |  |  |  |  |  |  |  |  |  |  |
|  |  |  |  |  |  |  |  |  |  |  |  |  |  |  |  |
| **295 K** |  |  | **ferric1** |  | **72.38**  (74) |  | 0.36040  (73) | n/a |  | 57.4291* |  | 0.491  (10) | 0.082  (28) | 0.61 | 1.12 |
|  |  |  |  |  |  |  |  |  |  | 43  (19) |  | 0.76  (11) | 0.207  (44) |  |  |
|  |  |  | **ferrous1** |  | **1.65**  (18) |  | 0.945  (25) | n/a |  | 100* |  | 0.785  (51) | 0.00039* | 0.78 |  |
|  |  |  | **OX1** |  | **9.01**  (46) |  | 0.371  (16) | -0.098  (16) |  | 100* |  | 49.29  (14) | 2.23  (18) | 49.29 |  |
|  |  |  |  |  |  |  |  |  |  |  |  |  |  |  |  |
| (BG = 3.8 MC/ch) |  |  | **(b)OxHy** |  | **16.96**  (72) |  | 0.3* | 0* |  | 100* |  | 0* | 37.1  (32) | 29.63 |  |
|  |  |  |  |  |  |  |  |  |  |  |  |  |  |  |  |
|  |  |  |  |  |  |  |  |  |  |  |  |  |  |  |  |
| **77 K** |  |  | **ferric1** |  | **3.07**  (32) |  | 0.450  (14) | n/a |  | 100* |  | 0.700  (25) | 0.213  (44) | 0.7 | 1.34 |
|  |  |  | **ferrous1** |  | **0.47**  (19) |  | 1.151  (62) | n/a |  | 100* |  | 1.74  (13) | 0.08916* | 1.74 |  |
|  |  |  | **OX1** |  | **9.18**  (56) |  | 0.4745  (46) | -0.0906  (46) |  | 100* |  | 52.473  (38) | 0.419  (79) | 52.47 |  |
|  |  |  | **OxHy1** |  | **65.7**  (22) |  | 0.4670  (47) | -0.1256  (45) |  | 58.2461* |  | 46.145  (75) | 3.45  (20) | 42.88 |  |
|  |  |  |  |  |  |  |  |  |  | 42  (11) |  | 38.3  (18) | 7.0  (11) |  |  |
| (BG = 3.8 MC/ch) |  |  | **(b)OxHy** |  | **21.6**  (24) |  | 0.5* | 0* |  | 100* |  | 0* | 24.3  (43) | 19.41 |  |
|  |  |  |  |  |  |  |  |  |  |  |  |  |  |  |  |
|  |  |  |  |  |  |  |  |  |  |  |  |  |  |  |  |
| **5 K** |  |  | **ferric1** |  | **1.31**  (22) |  | 0.459  (28) | n/a |  | 100* |  | 0.662  (50) | 0.2* | 0.66 | 2.3 |
|  |  |  | **ferrous1** |  | **0.47**  (15) |  | 0.927  (47) | n/a |  | 100* |  | 1.173  (92) | 1.797e^-05^* | 1.17 |  |
|  |  |  | **OX1** |  | **15.6**  (14) |  | 0.4821  (26) | -0.0926  (28) |  | 100* |  | 53.012  (25) | 0.419  (73) | 53.01 |  |
|  |  |  | **OxHy1** |  | **74.8**  (22) |  | 0.4748  (11) | -0.1122  (11) |  | 58.6023* |  | 49.206  (34) | 1.087  (63) | 48.51 |  |
|  |  |  |  |  |  |  |  |  |  | 41.4  (79) |  | 47.52  (58) | 2.76  (23) |  |  |
| (BG = 3.8 MC/ch) |  |  | **(b)OxHy** |  | **7.8**  (23) |  | 0.5* | 0* |  | 100* |  | 0* | 35  (14) | 28.02 |  |
|  |  |  |  |  |  |  |  |  |  |  |  |  |  |  |  |

BG = background level, in mega-counts per channel (MC/ch).

Phase = assigned spectral component of a certain mineral phase (ferrous1 = ferrous content of the sample; Ox1 = hematite; OxHy1 = goethite).

$\bar{\text{CS}}$ or δ_0_= the center shift of a Gaussian component in the quadrupole splitting distribution (QSD) of the hyperfine field distribution (HFD) of a given spectral component, given in mm s^‒1^.

$\bar{\text{QS}}$ = the center shift of a Gaussian component in the quadrupole splitting distribution (QSD) of the hyperfine field distribution (HFD) of a given spectral component, given in mm s^‒1^.

σ = the Gaussian standard deviation width of a given Gaussian component of a given QSD or HFD.

P = the weight factor (%) for a given Gaussian component in a given QSD or HFD.

H and $\bar{\text{H}}$ = the average magnitude of the hyperfine field (expressed as an excited state Zeeman splitting, in Torr (T), in a given HFD of a given sextet spectral component, or all components, respectively.

Δ = the average magnitude of the quadrupole splitting in a given QSD of a given doublet spectral component, given in mm s^‒1^.

$\bar{\text{ε}}$ = the average magnitude of the slave distribution of quadrupole shifts ($\text{ε}$) associated to a given HFD of a given sextet spectral component, given in mm s^‒1^.

$\text{Χ}_{\text{ν}}^{\text{2}}$ = the reduced chi-squared value for the fit: chi-squared ($\text{Χ}$^2^) divided by the number of degrees of freedom ($\text{ν}$). It has an ideal value of 1 for a correct model.

All fits performed using the Voigt-based fitting method of Rancourt and Ping ^3^ with the Recoil^TM^ software.

All fitting and calculated parameters are as defined in by Rancourt and Ping ^3^.

All errors are shown in parentheses in concise notation as the error on the last digit (e.g, 5.13 ± 0.25 is 5.13 (25))

All δ-1 couplings between CS and H (or DELTA) were taken to be zero.

All line-1 to line-2 area ratios in all (distributed) elemental doublets were taken to be 1.

All line-2/line-3 and line-1/line-3 area ratios in all (distributed and symmetric) elemental sextets were taken to be 2 and 3, respectively.

All ε-1 couplings between epsilon and H (in a HFD) are taken to be 0.

All Lorentzian half widths at half maximum (HWHM) are set at 0.1425 mm s^‒1^ as measured on Fe foil standards on the instrument.

All center shifts ($\bar{\text{CS}}$ or δ_0_) are given with respect to the CS of metallic Fe at 295 K.

**‛high clay‒high Fe’ Forest (5‒10 cm) 295 K**

**‛high clay‒high Fe’ Forest (5‒10 cm) 77 K**

**‛high clay‒high Fe’ Forest (5‒10 cm) 5 K**

| **Sample** |  |  | **Phase** |  | **Area** |  | $\bar{\text{CS}}$ **or δ_0_** | $\bar{\text{ε}}$ |  | **P** |  | **Δ or H** | **σ** | $\bar{\text{QS}}$ **or** $\bar{\text{H}}$ | $\text{Χ}_{\text{ν}}^{\text{2}}$ |
| --- | --- | --- | --- | --- | --- | --- | --- | --- | --- | --- | --- | --- | --- | --- | --- |
| **‛high clay‒high Fe’ Forest**  **(5‒10 cm)** |  |  |  |  | (%) |  | (mm s^‒1^) | |  | (%) |  | (mm s^‒1^) | | |  |
|  |  |  |  |  |  |  |  |  |  |  |  |  |  |  |  |
|  |  |  |  |  |  |  |  |  |  |  |  |  |  |  |  |
| **295 K** |  |  | **ferric1** |  | **75.1**  (10) |  | 0.36055  (86) | n/a |  | 63.2334* |  | 0.502  (17) | 0.097  (35) | 0.61 | 0.9 |
|  |  |  |  |  |  |  |  |  |  | 37  (29) |  | 0.80  (21) | 0.219  (86) |  |  |
|  |  |  | **ferrous1** |  | **2.02**  (25) |  | 0.93706* | n/a |  | 100* |  | 0.70945* | 0.00039* | 0.71 |  |
|  |  |  | **OX1** |  | **9.62**  (67) |  | 0.377  (21) | -0.096  (21) |  | 100* |  | 49.31  (19) | 2.12  (24) | 49.31 |  |
|  |  |  |  |  |  |  |  |  |  |  |  |  |  |  |  |
| (BG = 3.8 MC/ch) |  |  | **(b)OxHy** |  | **13.24**  (95) |  | 0.3* | 0* |  | 100* |  | 0* | 28.5  (42) | 22.77 |  |
|  |  |  |  |  |  |  |  |  |  |  |  |  |  |  |  |
|  |  |  |  |  |  |  |  |  |  |  |  |  |  |  |  |
| **77 K** |  |  | **ferric1** |  | **2.97**  (23) |  | 0.447  (13) | n/a |  | 100* |  | 0.654  (24) | 0.15* | 0.65 | 1.19 |
|  |  |  | **ferrous1** |  | **0.47**  (21) |  | 1.185  (63) | n/a |  | 100* |  | 1.63  (13) | 0.08916* | 1.63 |  |
|  |  |  | **OX1** |  | **9.19**  (57) |  | 0.4796  (55) | -0.0949  (55) |  | 100* |  | 52.411  (48) | 0.497  (88) | 52.41 |  |
|  |  |  | **OxHy1** |  | **62.7**  (18) |  | 0.4757  (53) | -0.1221  (50) |  | 55.2394* |  | 46.100  (84) | 3.20  (21) | 42.78 |  |
|  |  |  |  |  |  |  |  |  |  | 45  (10) |  | 38.7  (17) | 6.79  (100) |  |  |
| (BG = 3.8 MC/ch) |  |  | **(b)OxHy** |  | **24.7**  (19) |  | 0.5* | 0* |  | 100* |  | 0* | 27.1  (32) | 21.59 |  |
|  |  |  |  |  |  |  |  |  |  |  |  |  |  |  |  |
|  |  |  |  |  |  |  |  |  |  |  |  |  |  |  |  |
| **5 K** |  |  | **ferric1** |  | **1.25**  (25) |  | 0.453  (41) | n/a |  | 100* |  | 0.705  (72) | 0.2* | 0.7 | 1.38 |
|  |  |  | **ferrous1** |  | **0.61**  (21) |  | 0.857  (50) | n/a |  | 100* |  | 1.115  (94) | 1.797e^-05^* | 1.11 |  |
|  |  |  | **OX1** |  | **15.9**  (21) |  | 0.4806  (36) | -0.0916  (40) |  | 100* |  | 53.024  (40) | 0.47  (11) | 53.02 |  |
|  |  |  | **OxHy1** |  | **74.3**  (21) |  | 0.4752  (16) | -0.1104  (16) |  | 57.1202* |  | 49.233  (51) | 1.05  (11) | 48.48 |  |
|  |  |  |  |  |  |  |  |  |  | 43  (14) |  | 47.47  (97) | 2.66  (38) |  |  |
| (BG = 3.8 MC/ch) |  |  | **(b)OxHy** |  | **8.0**  (10) |  | 0.5* | 0* |  | 100* |  | 0* | 35.0  (71) | 27.89 |  |
|  |  |  |  |  |  |  |  |  |  |  |  |  |  |  |  |

BG = background level, in mega-counts per channel (MC/ch).

Phase = assigned spectral component of a certain mineral phase (ferrous1 = ferrous content of the sample; Ox1 = hematite; OxHy1 = goethite).

$\bar{\text{CS}}$ or δ_0_= the center shift of a Gaussian component in the quadrupole splitting distribution (QSD) of the hyperfine field distribution (HFD) of a given spectral component, given in mm s^‒1^.

$\bar{\text{QS}}$ = the center shift of a Gaussian component in the quadrupole splitting distribution (QSD) of the hyperfine field distribution (HFD) of a given spectral component, given in mm s^‒1^.

σ = the Gaussian standard deviation width of a given Gaussian component of a given QSD or HFD.

P = the weight factor (%) for a given Gaussian component in a given QSD or HFD.

H and $\bar{\text{H}}$ = the average magnitude of the hyperfine field (expressed as an excited state Zeeman splitting, in Torr (T), in a given HFD of a given sextet spectral component, or all components, respectively.

Δ = the average magnitude of the quadrupole splitting in a given QSD of a given doublet spectral component, given in mm s^‒1^.

$\bar{\text{ε}}$ = the average magnitude of the slave distribution of quadrupole shifts ($\text{ε}$) associated to a given HFD of a given sextet spectral component, given in mm s^‒1^.

$\text{Χ}_{\text{ν}}^{\text{2}}$ = the reduced chi-squared value for the fit: chi-squared ($\text{Χ}$^2^) divided by the number of degrees of freedom ($\text{ν}$). It has an ideal value of 1 for a correct model.

All fits performed using the Voigt-based fitting method of Rancourt and Ping ^3^ with the Recoil^TM^ software.

All fitting and calculated parameters are as defined in by Rancourt and Ping ^3^.

All errors are shown in parentheses in concise notation as the error on the last digit (e.g, 5.13 ± 0.25 is 5.13 (25))

All δ-1 couplings between CS and H (or DELTA) were taken to be zero.

All line-1 to line-2 area ratios in all (distributed) elemental doublets were taken to be 1.

All line-2/line-3 and line-1/line-3 area ratios in all (distributed and symmetric) elemental sextets were taken to be 2 and 3, respectively.

All ε-1 couplings between epsilon and H (in a HFD) are taken to be 0.

All Lorentzian half widths at half maximum (HWHM) are set at 0.1425 mm s^‒1^ as measured on Fe foil standards on the instrument.

All center shifts ($\bar{\text{CS}}$ or δ_0_) are given with respect to the CS of metallic Fe at 295 K.

**‛low clay‒low Fe’ Cropland (0‒5 cm) 295 K**

**‛low clay‒low Fe’ Cropland (0‒5 cm) 77 K**

**‛low clay‒low Fe’ Cropland (0‒5 cm) 5 K**

| **Sample** |  |  | **Phase** |  | **Area** |  | $\bar{\text{CS}}$ **or δ_0_** | $\bar{\text{ε}}$ |  | **P** |  | **Δ or H** | **σ** | $\bar{\text{QS}}$ **or** $\bar{\text{H}}$ | $\text{Χ}_{\text{ν}}^{\text{2}}$ |
| --- | --- | --- | --- | --- | --- | --- | --- | --- | --- | --- | --- | --- | --- | --- | --- |
| **‛low clay‒low Fe’ Cropland (0‒5 cm)** |  |  |  |  | (%) |  | (mm s^‒1^) | |  | (%) |  | (mm s^‒1^) | | |  |
|  |  |  |  |  |  |  |  |  |  |  |  |  |  |  |  |
|  |  |  |  |  |  |  |  |  |  |  |  |  |  |  |  |
| **295 K** |  |  | **ferric1** |  | **61.22**  (93) |  | 0.3650  (13) | n/a |  | 56.8968* |  | 0.493  (19) | 0.051  (71) | 0.6 | 1.12 |
|  |  |  |  |  |  |  |  |  |  | 43  (36) |  | 0.74  (19) | 0.178  (80) |  |  |
|  |  |  | **ferrous1** |  | **4.74**  (32) |  | 0.974  (15) | n/a |  | 100* |  | 0.847  (29) | 0.00039* | 0.85 |  |
|  |  |  | **OX1** |  | **12.40**  (57) |  | 0.375  (12) | -0.091  (12) |  | 100* |  | 50.837  (96) | 1.20  (13) | 50.84 |  |
|  |  |  |  |  |  |  |  |  |  |  |  |  |  |  |  |
| (BG = 3.8 MC/ch) |  |  | **(b)OxHy** |  | **21.6**  (10) |  | 0.5* | 0* |  | 100* |  | 0* | 40.3  (45) | 32.19 |  |
|  |  |  |  |  |  |  |  |  |  |  |  |  |  |  |  |
|  |  |  |  |  |  |  |  |  |  |  |  |  |  |  |  |
| **77 K** |  |  | **ferric1** |  | **4.77**  (36) |  | 0.453  (13) | n/a |  | 100* |  | 0.629  (24) | 0.15* | 0.63 | 0.97 |
|  |  |  | **ferrous1** |  | **3.04**  (34) |  | 1.08305* | n/a |  | 100* |  | 1.208* | 0.08916* | 1.21 |  |
|  |  |  | **OX1** |  | **8.52**  (95) |  | 0.480  (10) | -0.055  (10) |  | 100* |  | 52.943  (87) | 0.51  (17) | 52.94 |  |
|  |  |  | **OxHy1** |  | **56.1**  (19) |  | 0.4866  (95) | -0.1348  (92) |  | 59.0477* |  | 46.97  (18) | 3.33  (35) | 44.31 |  |
|  |  |  |  |  |  |  |  |  |  | 41  (14) |  | 40.5  (27) | 6.9  (12) |  |  |
| (BG = 3.8 MC/ch) |  |  | **(b)OxHy** |  | **27.6**  (21) |  | 0.5* | 0* |  | 100* |  | 0* | 37.0  (43) | 29.49 |  |
|  |  |  |  |  |  |  |  |  |  |  |  |  |  |  |  |
|  |  |  |  |  |  |  |  |  |  |  |  |  |  |  |  |
| **5 K** |  |  | **ferric1** |  | **2.48**  (34) |  | 0.520  (28) | n/a |  | 100* |  | 0.717  (49) | 0.2* | 0.72 | 1.17 |
|  |  |  | **ferrous1** |  | **0.91**  (27) |  | 1.045  (47) | n/a |  | 100* |  | 0.915  (89) | 1.797e^-05^* | 0.91 |  |
|  |  |  | **OX1** |  | **16.9**  (32) |  | 0.4898  (58) | -0.0670  (90) |  | 100* |  | 53.019  (89) | 0.75  (13) | 53.02 |  |
|  |  |  | **OxHy1** |  | **64.6**  (30) |  | 0.4782  (24) | -0.1136  (24) |  | 51.778* |  | 49.367  (69) | 0.87  (14) | 48.69 |  |
|  |  |  |  |  |  |  |  |  |  | 48  (17) |  | 48.0  (10) | 2.48  (39) |  |  |
| (BG = 3.8 MC/ch) |  |  | **(b)OxHy** |  | **15.1**  (15) |  | 0.5* | 0* |  | 100* |  | 0* | 42.5  (63) | 33.93 |  |
|  |  |  |  |  |  |  |  |  |  |  |  |  |  |  |  |

BG = background level, in mega-counts per channel (MC/ch).

Phase = assigned spectral component of a certain mineral phase (ferrous1 = ferrous content of the sample; Ox1 = hematite; OxHy1 = goethite).

$\bar{\text{CS}}$ or δ_0_= the center shift of a Gaussian component in the quadrupole splitting distribution (QSD) of the hyperfine field distribution (HFD) of a given spectral component, given in mm s^‒1^.

$\bar{\text{QS}}$ = the center shift of a Gaussian component in the quadrupole splitting distribution (QSD) of the hyperfine field distribution (HFD) of a given spectral component, given in mm s^‒1^.

σ = the Gaussian standard deviation width of a given Gaussian component of a given QSD or HFD.

P = the weight factor (%) for a given Gaussian component in a given QSD or HFD.

H and $\bar{\text{H}}$ = the average magnitude of the hyperfine field (expressed as an excited state Zeeman splitting, in Torr (T), in a given HFD of a given sextet spectral component, or all components, respectively.

Δ = the average magnitude of the quadrupole splitting in a given QSD of a given doublet spectral component, given in mm s^‒1^.

$\bar{\text{ε}}$ = the average magnitude of the slave distribution of quadrupole shifts ($\text{ε}$) associated to a given HFD of a given sextet spectral component, given in mm s^‒1^.

$\text{Χ}_{\text{ν}}^{\text{2}}$ = the reduced chi-squared value for the fit: chi-squared ($\text{Χ}$^2^) divided by the number of degrees of freedom ($\text{ν}$). It has an ideal value of 1 for a correct model.

All fits performed using the Voigt-based fitting method of Rancourt and Ping ^3^ with the Recoil^TM^ software.

All fitting and calculated parameters are as defined in by Rancourt and Ping ^3^.

All errors are shown in parentheses in concise notation as the error on the last digit (e.g, 5.13 ± 0.25 is 5.13 (25))

All δ-1 couplings between CS and H (or DELTA) were taken to be zero.

All line-1 to line-2 area ratios in all (distributed) elemental doublets were taken to be 1.

All line-2/line-3 and line-1/line-3 area ratios in all (distributed and symmetric) elemental sextets were taken to be 2 and 3, respectively.

All ε-1 couplings between epsilon and H (in a HFD) are taken to be 0.

All Lorentzian half widths at half maximum (HWHM) are set at 0.1425 mm s^‒1^ as measured on Fe foil standards on the instrument.

All center shifts ($\bar{\text{CS}}$ or δ_0_) are given with respect to the CS of metallic Fe at 295 K.

**‛low clay‒low Fe’ Cropland (5‒10 cm) 295 K**

**‛low clay‒low Fe’ Cropland (5‒10 cm) 77 K**

**‛low clay‒low Fe’ Cropland (5‒10 cm) 5 K**

| **Sample** |  |  | **Phase** |  | **Area** |  | $\bar{\text{CS}}$ **or δ_0_** | $\bar{\text{ε}}$ |  | **P** |  | **Δ or H** | **σ** | $\bar{\text{QS}}$ **or** $\bar{\text{H}}$ | $\text{Χ}_{\text{ν}}^{\text{2}}$ |
| --- | --- | --- | --- | --- | --- | --- | --- | --- | --- | --- | --- | --- | --- | --- | --- |
| **‛low clay‒low Fe’ Cropland (5‒10 cm)** |  |  |  |  | (%) |  | (mm s^‒1^) | |  | (%) |  | (mm s^‒1^) | | |  |
|  |  |  |  |  |  |  |  |  |  |  |  |  |  |  |  |
|  |  |  |  |  |  |  |  |  |  |  |  |  |  |  |  |
| **295 K** |  |  | **ferric1** |  | **63.58**  (69) |  | 0.36355  (87) | n/a |  | 65.7744* |  | 0.507  (12) | 0.076  (33) | 0.6 | 1.5 |
|  |  |  |  |  |  |  |  |  |  | 34  (24) |  | 0.78  (17) | 0.201  (72) |  |  |
|  |  |  | **ferrous1** |  | **3.57**  (22) |  | 0.964  (13) | n/a |  | 100* |  | 0.837  (27) | 0.00039* | 0.84 |  |
|  |  |  | **OX1** |  | **11.33**  (39) |  | 0.3642  (84) | -0.0963  (84) |  | 100* |  | 50.955  (69) | 1.095  (96) | 50.95 |  |
|  |  |  |  |  |  |  |  |  |  |  |  |  |  |  |  |
| (BG = 3.8 MC/ch) |  |  | **(b)OxHy** |  | **21.51**  (76) |  | 0.3* | 0* |  | 100* |  | 0* | 43.4  (35) | 34.6 |  |
|  |  |  |  |  |  |  |  |  |  |  |  |  |  |  |  |
|  |  |  |  |  |  |  |  |  |  |  |  |  |  |  |  |
| **77 K** |  |  | **ferric1** |  | **5.16**  (29) |  | 0.4801  (98) | n/a |  | 100* |  | 0.664  (17) | 0.15* | 0.66 | 1.43 |
|  |  |  | **ferrous1** |  | **2.79**  (24) |  | 1.107  (14) | n/a |  | 100* |  | 1.182  (29) | 0.08916* | 1.18 |  |
|  |  |  | **OX1** |  | **7.88**  (72) |  | 0.4854  (87) | -0.0485  (89) |  | 100* |  | 52.952  (75) | 0.57  (14) | 52.95 |  |
|  |  |  | **OxHy1** |  | **61.2**  (17) |  | 0.4771  (69) | -0.1402  (68) |  | 58.5179* |  | 46.88  (12) | 3.34  (22) | 43.78 |  |
|  |  |  |  |  |  |  |  |  |  | 41.5  (83) |  | 39.4  (17) | 7.57  (98) |  |  |
| (BG = 3.8 MC/ch) |  |  | **(b)OxHy** |  | **23.0**  (18) |  | 0.3* | 0* |  | 100* |  | 0* | 36.0  (37) | 28.72 |  |
|  |  |  |  |  |  |  |  |  |  |  |  |  |  |  |  |
|  |  |  |  |  |  |  |  |  |  |  |  |  |  |  |  |
| **5 K** |  |  | **ferric1** |  | **2.12**  (36) |  | 0.456  (35) | n/a |  | 100* |  | 0.651  (60) | 0.2* | 0.65 | 1.15 |
|  |  |  | **ferrous1** |  | **0.89**  (30) |  | 0.856  (49) | n/a |  | 100* |  | 1.036  (92) | 1.797e^-05^* | 1.04 |  |
|  |  |  | **OX1** |  | **16.2**  (30) |  | 0.4817  (56) | -0.0690  (84) |  | 100* |  | 53.032  (67) | 0.63  (14) | 53.03 |  |
|  |  |  | **OxHy1** |  | **69.8**  (29) |  | 0.4732  (23) | -0.1150  (23) |  | 54.0851* |  | 49.365  (61) | 0.90  (12) | 48.74 |  |
|  |  |  |  |  |  |  |  |  |  | 46  (14) |  | 47.99  (93) | 2.63  (37) |  |  |
| (BG = 3.8 MC/ch) |  |  | **(b)OxHy** |  | **11.0**  (13) |  | 0.5* | 0* |  | 100* |  | 0* | 32.0  (64) | 25.5 |  |
|  |  |  |  |  |  |  |  |  |  |  |  |  |  |  |  |

BG = background level, in mega-counts per channel (MC/ch).

Phase = assigned spectral component of a certain mineral phase (ferrous1 = ferrous content of the sample; Ox1 = hematite; OxHy1 = goethite).

$\bar{\text{CS}}$ or δ_0_= the center shift of a Gaussian component in the quadrupole splitting distribution (QSD) of the hyperfine field distribution (HFD) of a given spectral component, given in mm s^‒1^.

$\bar{\text{QS}}$ = the center shift of a Gaussian component in the quadrupole splitting distribution (QSD) of the hyperfine field distribution (HFD) of a given spectral component, given in mm s^‒1^.

σ = the Gaussian standard deviation width of a given Gaussian component of a given QSD or HFD.

P = the weight factor (%) for a given Gaussian component in a given QSD or HFD.

H and $\bar{\text{H}}$ = the average magnitude of the hyperfine field (expressed as an excited state Zeeman splitting, in Torr (T), in a given HFD of a given sextet spectral component, or all components, respectively.

Δ = the average magnitude of the quadrupole splitting in a given QSD of a given doublet spectral component, given in mm s^‒1^.

$\bar{\text{ε}}$ = the average magnitude of the slave distribution of quadrupole shifts ($\text{ε}$) associated to a given HFD of a given sextet spectral component, given in mm s^‒1^.

$\text{Χ}_{\text{ν}}^{\text{2}}$ = the reduced chi-squared value for the fit: chi-squared ($\text{Χ}$^2^) divided by the number of degrees of freedom ($\text{ν}$). It has an ideal value of 1 for a correct model.

All fits performed using the Voigt-based fitting method of Rancourt and Ping ^3^ with the Recoil^TM^ software.

All fitting and calculated parameters are as defined in by Rancourt and Ping ^3^.

All errors are shown in parentheses in concise notation as the error on the last digit (e.g, 5.13 ± 0.25 is 5.13 (25))

All δ-1 couplings between CS and H (or DELTA) were taken to be zero.

All line-1 to line-2 area ratios in all (distributed) elemental doublets were taken to be 1.

All line-2/line-3 and line-1/line-3 area ratios in all (distributed and symmetric) elemental sextets were taken to be 2 and 3, respectively.

All ε-1 couplings between epsilon and H (in a HFD) are taken to be 0.

All Lorentzian half widths at half maximum (HWHM) are set at 0.1425 mm s^‒1^ as measured on Fe foil standards on the instrument.

All center shifts ($\bar{\text{CS}}$ or δ_0_) are given with respect to the CS of metallic Fe at 295 K.

**‛low clay‒high Fe’ Cropland (0‒5 cm) 295 K**

**‛low clay‒high Fe’ Cropland (0‒5 cm) 77 K**

**‛low clay‒high Fe’ Cropland (0‒5 cm) 5 K**

| **Sample** |  |  | **Phase** |  | **Area** |  | $\bar{\text{CS}}$ **or δ_0_** | $\bar{\text{ε}}$ |  | **P** |  | **Δ or H** | **σ** | $\bar{\text{QS}}$ **or** $\bar{\text{H}}$ | $\text{Χ}_{\text{ν}}^{\text{2}}$ |
| --- | --- | --- | --- | --- | --- | --- | --- | --- | --- | --- | --- | --- | --- | --- | --- |
| **‛low clay‒high Fe’ Cropland (0‒5 cm)** |  |  |  |  | (%) |  | (mm s^‒1^) | |  | (%) |  | (mm s^‒1^) | | |  |
|  |  |  |  |  |  |  |  |  |  |  |  |  |  |  |  |
|  |  |  |  |  |  |  |  |  |  |  |  |  |  |  |  |
| **295 K** |  |  | **ferric1** |  | **61.20**  (53) |  | 0.36252  (65) | n/a |  | 71.8576* |  | 0.5309  (62) | 0.155  (20) | 0.63 | 2.25 |
|  |  |  |  |  |  |  |  |  |  | 28  (17) |  | 0.88  (20) | 0.312  (74) |  |  |
|  |  |  | **ferrous1** |  | **2.73**  (15) |  | 0.951  (11) | n/a |  | 100* |  | 0.864  (21) | 0.00039* | 0.86 |  |
|  |  |  | **OX1** |  | **13.81**  (37) |  | 0.3709  (92) | -0.0957  (92) |  | 100* |  | 48.404  (80) | 2.50  (10) | 48.4 |  |
|  |  |  |  |  |  |  |  |  |  |  |  |  |  |  |  |
| (BG = 3.8 MC/ch) |  |  | **(b)OxHy** |  | **22.26**  (57) |  | 0.3* | 0* |  | 100* |  | 0* | 39.6  (22) | 31.62 |  |
|  |  |  |  |  |  |  |  |  |  |  |  |  |  |  |  |
|  |  |  |  |  |  |  |  |  |  |  |  |  |  |  |  |
| **77 K** |  |  | **ferric1** |  | **2.04**  (21) |  | 0.465  (14) | n/a |  | 100* |  | 0.701  (28) | 0.15* | 0.7 | 1.55 |
|  |  |  | **ferrous1** |  | **1.05**  (16) |  | 1.319  (25) | n/a |  | 100* |  | 1.333  (46) | 0.0891* | 1.33 |  |
|  |  |  | **OX1** |  | **9.17**  (54) |  | 0.4716  (43) | -0.0882  (43) |  | 100* |  | 52.448  (36) | 0.457  (75) | 52.45 |  |
|  |  |  | **OxHy1** |  | **68.7**  (17) |  | 0.4671  (39) | -0.1219  (38) |  | 55.9041* |  | 46.802  (72) | 3.30  (19) | 43.41 |  |
|  |  |  |  |  |  |  |  |  |  | 44.1  (91) |  | 39.1  (14) | 6.67  (86) |  |  |
| (BG = 3.8 MC/ch) |  |  | **(b)OxHy** |  | **19.0**  (18) |  | 0.5* | 0* |  | 100* |  | 0* | 24.3  (36) | 19.4 |  |
|  |  |  |  |  |  |  |  |  |  |  |  |  |  |  |  |
|  |  |  |  |  |  |  |  |  |  |  |  |  |  |  |  |
| **5 K** |  |  | **ferric1** |  | **0.96**  (15) |  | 0.462  (33) | n/a |  | 100* |  | 0.852  (58) | 0.2* | 0.85 | 2.83 |
|  |  |  | **ferrous1** |  | **0.59**  (13) |  | 0.931  (33) | n/a |  | 100* |  | 1.122  (63) | 1.797e^-05^* | 1.12 |  |
|  |  |  | **OX1** |  | **16.4**  (11) |  | 0.4810  (22) | -0.0950  (24) |  | 100* |  | 52.927  (23) | 0.475  (57) | 52.93 |  |
|  |  |  | **OxHy1** |  | **72.8**  (11) |  | 0.4746  (11) | -0.1152  (10) |  | 62.4611* |  | 49.265  (27) | 1.181  (58) | 48.5 |  |
|  |  |  |  |  |  |  |  |  |  | 37.5  (81) |  | 47.24  (67) | 2.79  (27) |  |  |
| (BG = 3.8 MC/ch) |  |  | **(b)OxHy** |  | **9.22**  (67) |  | 0.5* | 0* |  | 100* |  | 0* | 40.2  (46) | 32.07 |  |
|  |  |  |  |  |  |  |  |  |  |  |  |  |  |  |  |

BG = background level, in mega-counts per channel (MC/ch).

Phase = assigned spectral component of a certain mineral phase (ferrous1 = ferrous content of the sample; Ox1 = hematite; OxHy1 = goethite).

$\bar{\text{CS}}$ or δ_0_= the center shift of a Gaussian component in the quadrupole splitting distribution (QSD) of the hyperfine field distribution (HFD) of a given spectral component, given in mm s^‒1^.

$\bar{\text{QS}}$ = the center shift of a Gaussian component in the quadrupole splitting distribution (QSD) of the hyperfine field distribution (HFD) of a given spectral component, given in mm s^‒1^.

σ = the Gaussian standard deviation width of a given Gaussian component of a given QSD or HFD.

P = the weight factor (%) for a given Gaussian component in a given QSD or HFD.

H and $\bar{\text{H}}$ = the average magnitude of the hyperfine field (expressed as an excited state Zeeman splitting, in Torr (T), in a given HFD of a given sextet spectral component, or all components, respectively.

Δ = the average magnitude of the quadrupole splitting in a given QSD of a given doublet spectral component, given in mm s^‒1^.

$\bar{\text{ε}}$ = the average magnitude of the slave distribution of quadrupole shifts ($\text{ε}$) associated to a given HFD of a given sextet spectral component, given in mm s^‒1^.

$\text{Χ}_{\text{ν}}^{\text{2}}$ = the reduced chi-squared value for the fit: chi-squared ($\text{Χ}$^2^) divided by the number of degrees of freedom ($\text{ν}$). It has an ideal value of 1 for a correct model.

All fits performed using the Voigt-based fitting method of Rancourt and Ping ^3^ with the Recoil^TM^ software.

All fitting and calculated parameters are as defined in by Rancourt and Ping ^3^.

All errors are shown in parentheses in concise notation as the error on the last digit (e.g, 5.13 ± 0.25 is 5.13 (25))

All δ-1 couplings between CS and H (or DELTA) were taken to be zero.

All line-1 to line-2 area ratios in all (distributed) elemental doublets were taken to be 1.

All line-2/line-3 and line-1/line-3 area ratios in all (distributed and symmetric) elemental sextets were taken to be 2 and 3, respectively.

All ε-1 couplings between epsilon and H (in a HFD) are taken to be 0.

All Lorentzian half widths at half maximum (HWHM) are set at 0.1425 mm s^‒1^ as measured on Fe foil standards on the instrument.

All center shifts ($\bar{\text{CS}}$ or δ_0_) are given with respect to the CS of metallic Fe at 295 K.

**‛low clay‒high Fe’ Cropland (5‒10 cm) 295 K**

**‛low clay‒high Fe’ Cropland (5‒10 cm) 77 K**

**‛low clay‒high Fe’ Cropland (5‒10 cm) 5 K**

| **Sample** |  |  | **Phase** |  | **Area** |  | $\bar{\text{CS}}$ **or δ_0_** | $\bar{\text{ε}}$ |  | **P** |  | **Δ or H** | **σ** | $\bar{\text{QS}}$ **or** $\bar{\text{H}}$ | $\text{Χ}_{\text{ν}}^{\text{2}}$ |
| --- | --- | --- | --- | --- | --- | --- | --- | --- | --- | --- | --- | --- | --- | --- | --- |
| **‛low clay‒high Fe’ Cropland (5‒10 cm)** |  |  |  |  | (%) |  | (mm s^‒1^) | |  | (%) |  | (mm s^‒1^) | | |  |
|  |  |  |  |  |  |  |  |  |  |  |  |  |  |  |  |
|  |  |  |  |  |  |  |  |  |  |  |  |  |  |  |  |
| **295 K** |  |  | **ferric1** |  | **63.6**  (31) |  | 0.36114  (94) | n/a |  | 77.2972* |  | 0.5399  (71) | 0.162  (22) | 0.63 | 1.49 |
|  |  |  |  |  |  |  |  |  |  | 23  (18) |  | 0.94  (30) | 0.34  (12) |  |  |
|  |  |  | **ferrous1** |  | **2.59**  (26) |  | 0.966  (16) | n/a |  | 100* |  | 0.862  (32) | 0.00039* | 0.86 |  |
|  |  |  | **OX1** |  | **13.49**  (81) |  | 0.359  (12) | -0.106  (12) |  | 100* |  | 48.53  (10) | 2.19  (13) | 48.53 |  |
|  |  |  |  |  |  |  |  |  |  |  |  |  |  |  |  |
| (BG = 3.8 MC/ch) |  |  | **(b)OxHy** |  | **20.4**  (39) |  | 0.5* | 0* |  | 100* |  | 0* | 41  (11) | 33.09 |  |
|  |  |  |  |  |  |  |  |  |  |  |  |  |  |  |  |
|  |  |  |  |  |  |  |  |  |  |  |  |  |  |  |  |
| **77 K** |  |  | **ferric1** |  | **2.29**  (22) |  | 0.469  (13) | n/a |  | 100* |  | 0.690  (25) | 0.15* | 0.69 | 1.66 |
|  |  |  | **ferrous1** |  | **1.17**  (16) |  | 1.189  (24) | n/a |  | 100* |  | 1.087  (48) | 0.08916* | 1.09 |  |
|  |  |  | **OX1** |  | **9.43**  (56) |  | 0.4786  (44) | -0.0933  (44) |  | 100* |  | 52.459  (38) | 0.472  (76) | 52.46 |  |
|  |  |  | **OxHy1** |  | **68.3**  (18) |  | 0.4709  (42) | -0.1259  (40) |  | 58.283* |  | 46.662  (71) | 3.33  (19) | 43.41 |  |
|  |  |  |  |  |  |  |  |  |  | 41.7  (92) |  | 38.9  (15) | 6.84  (96) |  |  |
| (BG = 3.8 MC/ch) |  |  | **(b)OxHy** |  | **18.8**  (20) |  | 0.5* | 0* |  | 100* |  | 0* | 24.6  (41) | 19.59 |  |
|  |  |  |  |  |  |  |  |  |  |  |  |  |  |  |  |
|  |  |  |  |  |  |  |  |  |  |  |  |  |  |  |  |
| **5 K** |  |  | **ferric1** |  | **1.02**  (16) |  | 0.582  (35) | n/a |  | 100* |  | 0.800  (59) | 0.2* | 0.8 | 2.27 |
|  |  |  | **ferrous1** |  | **0.39**  (13) |  | 1.053  (57) | n/a |  | 100* |  | 0.90  (10) | 1.797e^-05^* | 0.9 |  |
|  |  |  | **OX1** |  | **17.2**  (13) |  | 0.4788  (23) | -0.0938  (25) |  | 100* |  | 52.912  (23) | 0.444  (62) | 52.91 |  |
|  |  |  | **OxHy1** |  | **72.6**  (13) |  | 0.4745  (11) | -0.1156  (11) |  | 61.0184* |  | 49.309  (31) | 1.100  (60) | 48.62 |  |
|  |  |  |  |  |  |  |  |  |  | 39.0  (84) |  | 47.53  (65) | 2.70  (25) |  |  |
| (BG = 3.8 MC/ch) |  |  | **(b)OxHy** |  | **8.79**  (72) |  | 0.5* | 0* |  | 100* |  | 0* | 39.8  (52) | 31.74 |  |
|  |  |  |  |  |  |  |  |  |  |  |  |  |  |  |  |

BG = background level, in mega-counts per channel (MC/ch).

Phase = assigned spectral component of a certain mineral phase (ferrous1 = ferrous content of the sample; Ox1 = hematite; OxHy1 = goethite).

$\bar{\text{CS}}$ or δ_0_= the center shift of a Gaussian component in the quadrupole splitting distribution (QSD) of the hyperfine field distribution (HFD) of a given spectral component, given in mm s^‒1^.

$\bar{\text{QS}}$ = the center shift of a Gaussian component in the quadrupole splitting distribution (QSD) of the hyperfine field distribution (HFD) of a given spectral component, given in mm s^‒1^.

σ = the Gaussian standard deviation width of a given Gaussian component of a given QSD or HFD.

P = the weight factor (%) for a given Gaussian component in a given QSD or HFD.

H and $\bar{\text{H}}$ = the average magnitude of the hyperfine field (expressed as an excited state Zeeman splitting, in Torr (T), in a given HFD of a given sextet spectral component, or all components, respectively.

Δ = the average magnitude of the quadrupole splitting in a given QSD of a given doublet spectral component, given in mm s^‒1^.

$\bar{\text{ε}}$ = the average magnitude of the slave distribution of quadrupole shifts ($\text{ε}$) associated to a given HFD of a given sextet spectral component, given in mm s^‒1^.

$\text{Χ}_{\text{ν}}^{\text{2}}$ = the reduced chi-squared value for the fit: chi-squared ($\text{Χ}$^2^) divided by the number of degrees of freedom ($\text{ν}$). It has an ideal value of 1 for a correct model.

All fits performed using the Voigt-based fitting method of Rancourt and Ping ^3^ with the Recoil^TM^ software.

All fitting and calculated parameters are as defined in by Rancourt and Ping ^3^.

All errors are shown in parentheses in concise notation as the error on the last digit (e.g, 5.13 ± 0.25 is 5.13 (25))

All δ-1 couplings between CS and H (or DELTA) were taken to be zero.

All line-1 to line-2 area ratios in all (distributed) elemental doublets were taken to be 1.

All line-2/line-3 and line-1/line-3 area ratios in all (distributed and symmetric) elemental sextets were taken to be 2 and 3, respectively.

All ε-1 couplings between epsilon and H (in a HFD) are taken to be 0.

All Lorentzian half widths at half maximum (HWHM) are set at 0.1425 mm s^‒1^ as measured on Fe foil standards on the instrument.

All center shifts ($\bar{\text{CS}}$ or δ_0_) are given with respect to the CS of metallic Fe at 295 K.

**‛high clay‒high Fe’ Cropland (0‒5 cm) 295 K**

**‛high clay‒high Fe’ Cropland (0‒5 cm) 77 K**

**‛high clay‒high Fe’ Cropland (0‒5 cm) 5 K**

| **Sample** |  |  | **Phase** |  | **Area** |  | $\bar{\text{CS}}$ **or δ_0_** | $\bar{\text{ε}}$ |  | **P** |  | **Δ or H** | **σ** | $\bar{\text{QS}}$ **or** $\bar{\text{H}}$ | $\text{Χ}_{\text{ν}}^{\text{2}}$ |
| --- | --- | --- | --- | --- | --- | --- | --- | --- | --- | --- | --- | --- | --- | --- | --- |
| **‛high clay‒high Fe’ Cropland (0‒5 cm)** |  |  |  |  | (%) |  | (mm s^‒1^) | |  | (%) |  | (mm s^‒1^) | | |  |
|  |  |  |  |  |  |  |  |  |  |  |  |  |  |  |  |
|  |  |  |  |  |  |  |  |  |  |  |  |  |  |  |  |
| **295 K** |  |  | **ferric1** |  | **69.6**  (46) |  | 0.3637  (14) | n/a |  | 63.4833* |  | 0.498  (11) | 0.080  (37) | 0.6 | 0.75 |
|  |  |  |  |  |  |  |  |  |  | 37  (23) |  | 0.78  (16) | 0.220  (67) |  |  |
|  |  |  | **ferrous1** |  | **1.87**  (32) |  | 0.971  (40) | n/a |  | 100* |  | 0.808  (82) | 0.00039* | 0.81 |  |
|  |  |  | **OX1** |  | **7.29**  (89) |  | 0.329  (31) | -0.122  (31) |  | 100* |  | 48.38  (27) | 2.16  (35) | 48.38 |  |
|  |  |  |  |  |  |  |  |  |  |  |  |  |  |  |  |
| (BG = 3.8 MC/ch) |  |  | **(b)OxHy** |  | **21.2**  (51) |  | 0.3* | 0* |  | 100* |  | 0* | 39  (14) | 31.07 |  |
|  |  |  |  |  |  |  |  |  |  |  |  |  |  |  |  |
|  |  |  |  |  |  |  |  |  |  |  |  |  |  |  |  |
| **77 K** |  |  | **ferric1** |  | **3.87**  (35) |  | 0.460  (15) | n/a |  | 100* |  | 0.632  (27) | 0.15* | 0.63 | 0.92 |
|  |  |  | **ferrous1** |  | **0.9**  3(30) |  | 1.250  (50) | n/a |  | 100* |  | 1.520  (96) | 0.08916* | 1.52 |  |
|  |  |  | **OX1** |  | **6.13**  (76) |  | 0.475  (11) | -0.085  (11) |  | 100* |  | 52.408  (89) | 0.24  (26) | 52.41 |  |
|  |  |  | **OxHy1** |  | **72.4**  (22) |  | 0.4694  (50) | -0.1248  (49) |  | 57.1894* |  | 47.091  (77) | 2.50  (15) | 44.72 |  |
|  |  |  |  |  |  |  |  |  |  | 42.8  (75) |  | 41.6  (11) | 5.98  (62) |  |  |
| (BG = 3.8 MC/ch) |  |  | **(b)OxHy** |  | **16.6**  (23) |  | 0.5* | 0* |  | 100* |  | 0* | 27.1  (61) | 21.65 |  |
|  |  |  |  |  |  |  |  |  |  |  |  |  |  |  |  |
|  |  |  |  |  |  |  |  |  |  |  |  |  |  |  |  |
| **5 K** |  |  | **ferric1** |  | **1.98**  (24) |  | 0.474  (26) | n/a |  | 100* |  | 0.588  (47) | 0.2* | 0.59 | 1.34 |
|  |  |  | **ferrous1** |  | **0.40**  (21) |  | 0.919  (76) | n/a |  | 100* |  | 1.14  (15) | 1.797e^-05^* | 1.14 |  |
|  |  |  | **OX1** |  | **14.0**  (26) |  | 0.4759  (42) | -0.0966  (50) |  | 100* |  | 52.926  (54) | 0.48  (13) | 52.93 |  |
|  |  |  | **OxHy1** |  | **74.3**  (24) |  | 0.4760  (14) | -0.1183  (14) |  | 54.0143* |  | 49.458  (46) | 0.846  (89) | 48.86 |  |
|  |  |  |  |  |  |  |  |  |  | 46  (12) |  | 48.15  (72) | 2.42  (27) |  |  |
| (BG = 3.8 MC/ch) |  |  | **(b)OxHy** |  | **9.38**  (99) |  | 0.5* | 0* |  | 100* |  | 0* | 37.2  (62) | 29.67 |  |
|  |  |  |  |  |  |  |  |  |  |  |  |  |  |  |  |

BG = background level, in mega-counts per channel (MC/ch).

Phase = assigned spectral component of a certain mineral phase (ferrous1 = ferrous content of the sample; Ox1 = hematite; OxHy1 = goethite).

$\bar{\text{CS}}$ or δ_0_= the center shift of a Gaussian component in the quadrupole splitting distribution (QSD) of the hyperfine field distribution (HFD) of a given spectral component, given in mm s^‒1^.

$\bar{\text{QS}}$ = the center shift of a Gaussian component in the quadrupole splitting distribution (QSD) of the hyperfine field distribution (HFD) of a given spectral component, given in mm s^‒1^.

σ = the Gaussian standard deviation width of a given Gaussian component of a given QSD or HFD.

P = the weight factor (%) for a given Gaussian component in a given QSD or HFD.

H and $\bar{\text{H}}$ = the average magnitude of the hyperfine field (expressed as an excited state Zeeman splitting, in Torr (T), in a given HFD of a given sextet spectral component, or all components, respectively.

Δ = the average magnitude of the quadrupole splitting in a given QSD of a given doublet spectral component, given in mm s^‒1^.

$\bar{\text{ε}}$ = the average magnitude of the slave distribution of quadrupole shifts ($\text{ε}$) associated to a given HFD of a given sextet spectral component, given in mm s^‒1^.

$\text{Χ}_{\text{ν}}^{\text{2}}$ = the reduced chi-squared value for the fit: chi-squared ($\text{Χ}$^2^) divided by the number of degrees of freedom ($\text{ν}$). It has an ideal value of 1 for a correct model.

All fits performed using the Voigt-based fitting method of Rancourt and Ping ^3^ with the Recoil^TM^ software.

All fitting and calculated parameters are as defined in by Rancourt and Ping ^3^.

All errors are shown in parentheses in concise notation as the error on the last digit (e.g, 5.13 ± 0.25 is 5.13 (25))

All δ-1 couplings between CS and H (or DELTA) were taken to be zero.

All line-1 to line-2 area ratios in all (distributed) elemental doublets were taken to be 1.

All line-2/line-3 and line-1/line-3 area ratios in all (distributed and symmetric) elemental sextets were taken to be 2 and 3, respectively.

All ε-1 couplings between epsilon and H (in a HFD) are taken to be 0.

All Lorentzian half widths at half maximum (HWHM) are set at 0.1425 mm s^‒1^ as measured on Fe foil standards on the instrument.

All center shifts ($\bar{\text{CS}}$ or δ_0_) are given with respect to the CS of metallic Fe at 295 K.

**‛high clay‒high Fe’ Cropland (5‒10 cm) 295 K**

**‛high clay‒high Fe’ Cropland (5‒10 cm) 77 K**

**‛high clay‒high Fe’ Cropland (5‒10 cm) 5 K**

| **Sample** |  |  | **Phase** |  | **Area** |  | $\bar{\text{CS}}$ **or δ_0_** | $\bar{\text{ε}}$ |  | **P** |  | **Δ or H** | **σ** | $\bar{\text{QS}}$ **or** $\bar{\text{H}}$ | $\text{Χ}_{\text{ν}}^{\text{2}}$ |
| --- | --- | --- | --- | --- | --- | --- | --- | --- | --- | --- | --- | --- | --- | --- | --- |
| **‛high clay‒high Fe’ Cropland (5‒10 cm)** |  |  |  |  | (%) |  | (mm s^‒1^) | |  | (%) |  | (mm s^‒1^) | | |  |
|  |  |  |  |  |  |  |  |  |  |  |  |  |  |  |  |
|  |  |  |  |  |  |  |  |  |  |  |  |  |  |  |  |
| **295 K** |  |  | **ferric1** |  | **71.52**  (62) |  | 0.36290  (67) | n/a |  | 66.2779* |  | 0.5015  (64) | 0.090  (18) | 0.6 | 1.41 |
|  |  |  |  |  |  |  |  |  |  | 34  (14) |  | 0.79  (10) | 0.221  (42) |  |  |
|  |  |  | **ferrous1** |  | **2.12**  (14) |  | 0.960  (17) | n/a |  | 100* |  | 0.785  (36) | 0.00039* | 0.79 |  |
|  |  |  | **OX1** |  | **7.39**  (41) |  | 0.359  (18) | -0.122  (18) |  | 100* |  | 48.09  (16) | 2.67  (21) | 48.09 |  |
|  |  |  |  |  |  |  |  |  |  |  |  |  |  |  |  |
| (BG = 3.8 MC/ch) |  |  | **(b)OxHy** |  | **18.98**  (60) |  | 0.3* | 0* |  | 100* |  | 0* | 38.9  (24) | 31.02 |  |
|  |  |  |  |  |  |  |  |  |  |  |  |  |  |  |  |
|  |  |  |  |  |  |  |  |  |  |  |  |  |  |  |  |
| **77 K** |  |  | **ferric1** |  | **3.31**  (25) |  | 0.430  (15) | n/a |  | 100* |  | 0.620  (22) | 0.15* | 0.62 | 1.38 |
|  |  |  | **ferrous1** |  | **1.00**  (23) |  | 0.757  (39) | n/a |  | 100* |  | 0.803  (62) | 0.08916* | 0.8 |  |
|  |  |  | **OX1** |  | **5.61**  (65) |  | 0.4677  (95) | -0.0895  (96) |  | 100* |  | 52.410  (84) | 0.41  (18) | 52.41 |  |
|  |  |  | **OxHy1** |  | **71.0**  (14) |  | 0.4755  (35) | -0.1273  (34) |  | 50.3047* |  | 47.027  (58) | 2.21  (10) | 44.83 |  |
|  |  |  |  |  |  |  |  |  |  | 49.7  (50) |  | 42.61  (63) | 5.54  (32) |  |  |
| (BG = 3.8 MC/ch) |  |  | **(b)OxHy** |  | **19.0**  (14) |  | 0.5* | 0* |  | 100* |  | 0* | 29.4  (37) | 23.48 |  |
|  |  |  |  |  |  |  |  |  |  |  |  |  |  |  |  |
|  |  |  |  |  |  |  |  |  |  |  |  |  |  |  |  |
| **5 K** |  |  | **ferric1** |  | **2.08**  (24) |  | 0.475  (27) | n/a |  | 100* |  | 0.647  (45) | 0.2* | 0.65 | 1.37 |
|  |  |  | **ferrous1** |  | **0.65**  (22) |  | 0.971  (49) | n/a |  | 100* |  | 1.053  (94) | 1.797e^-05^* | 1.05 |  |
|  |  |  | **OX1** |  | **11.1**  (20) |  | 0.4778  (54) | -0.0940  (61) |  | 100* |  | 52.949  (52) | 0.45  (15) | 52.95 |  |
|  |  |  | **OxHy1** |  | **77.9**  (20) |  | 0.4778  (15) | -0.1193  (14) |  | 55.553* |  | 49.396  (34) | 0.889  (61) | 48.87 |  |
|  |  |  |  |  |  |  |  |  |  | 44.4  (72) |  | 48.21  (50) | 2.70  (20) |  |  |
| (BG = 3.8 MC/ch) |  |  | **(b)OxHy** |  | **8.2**  (11) |  | 0.5* | 0* |  | 100* |  | 0* | 39.8  (83) | 31.75 |  |
|  |  |  |  |  |  |  |  |  |  |  |  |  |  |  |  |

BG = background level, in mega-counts per channel (MC/ch).

Phase = assigned spectral component of a certain mineral phase (ferrous1 = ferrous content of the sample; Ox1 = hematite; OxHy1 = goethite).

$\bar{\text{CS}}$ or δ_0_= the center shift of a Gaussian component in the quadrupole splitting distribution (QSD) of the hyperfine field distribution (HFD) of a given spectral component, given in mm s^‒1^.

$\bar{\text{QS}}$ = the center shift of a Gaussian component in the quadrupole splitting distribution (QSD) of the hyperfine field distribution (HFD) of a given spectral component, given in mm s^‒1^.

σ = the Gaussian standard deviation width of a given Gaussian component of a given QSD or HFD.

P = the weight factor (%) for a given Gaussian component in a given QSD or HFD.

H and $\bar{\text{H}}$ = the average magnitude of the hyperfine field (expressed as an excited state Zeeman splitting, in Torr (T), in a given HFD of a given sextet spectral component, or all components, respectively.

Δ = the average magnitude of the quadrupole splitting in a given QSD of a given doublet spectral component, given in mm s^‒1^.

$\bar{\text{ε}}$ = the average magnitude of the slave distribution of quadrupole shifts ($\text{ε}$) associated to a given HFD of a given sextet spectral component, given in mm s^‒1^.

$\text{Χ}_{\text{ν}}^{\text{2}}$ = the reduced chi-squared value for the fit: chi-squared ($\text{Χ}$^2^) divided by the number of degrees of freedom ($\text{ν}$). It has an ideal value of 1 for a correct model.

All fits performed using the Voigt-based fitting method of Rancourt and Ping ^3^ with the Recoil^TM^ software.

All fitting and calculated parameters are as defined in by Rancourt and Ping ^3^.

All errors are shown in parentheses in concise notation as the error on the last digit (e.g, 5.13 ± 0.25 is 5.13 (25))

All δ-1 couplings between CS and H (or DELTA) were taken to be zero.

All line-1 to line-2 area ratios in all (distributed) elemental doublets were taken to be 1.

All line-2/line-3 and line-1/line-3 area ratios in all (distributed and symmetric) elemental sextets were taken to be 2 and 3, respectively.

All ε-1 couplings between epsilon and H (in a HFD) are taken to be 0.

All Lorentzian half widths at half maximum (HWHM) are set at 0.1425 mm s^‒1^ as measured on Fe foil standards on the instrument.

All center shifts ($\bar{\text{CS}}$ or δ_0_) are given with respect to the CS of metallic Fe at 295 K.

**Figure S3:** Average solid-state CPMAS ^13^C-NMR spectra of forest and cropland litter material (Oi horizons) and related standard deviation, spectra were referenced to 0 ppm as related to tetramethylsilane. Forest (*n* = 6) and cropland (*n* = 3).

**
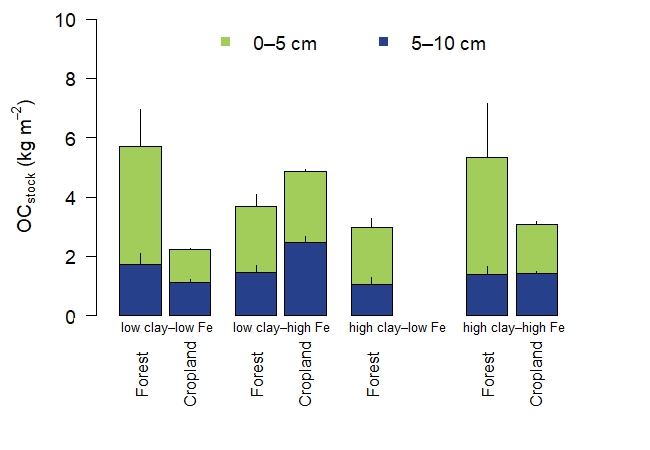
Figure S4:** Bulk organic carbon (OC) stocks of the mineral combinations under forest and cropland land-use. Sample numbers for the combinations are as follows: ‛low clay‒low Fe’ under forest (*n* = 4), ‛low clay‒high Fe’ under forest (*n* = 4), ‛high clay‒low Fe’ under forest (*n* = 3), ‛high clay‒high Fe’ under forest (*n* = 7); all cropland combinations (*n* = 3).

**Figure S5**: Mean bulk organic carbon (OC) stock changes in 0‒10 cm soil depth during forest to cropland conversion.

Section 9: References

1. FAO. *World Reference Base for Soil Resources 2014. International soil classification system for naming soils and creating legends for soil maps* (FAO, Rome, 2014).

2. Becquer, T. *et al.* Mineralogical, chemical and charge properties of Geric Ferralsols from New Caledonia. *Geoderma* **103,** 291–306; 10.1016/S0016-7061(01)00045-3 (2001).

3. Rancourt, D. G. & Ping, J. Y. Voigt-based methods for arbitrary-shape static hyperfine parameter distributions in Mössbauer spectroscopy. *Nuclear Instruments and Methods in Physics Research Section B: Beam Interactions with Materials and Atoms* **58,** 85–97; 10.1016/0168-583X(91)95681-3 (1991).

4. Rancourt, D. G. Mössbauer spectroscopy in clay science. *Hyperfine Interact.* **117,** 3–38; 10.1023/A:1012651628508 (1998).

5. Lalonde, A. E., Rancourt, D. G. & Ping, J. Y. Accuracy of ferric/ferrous determinations in micas: A comparison of Mössbauer spectroscopy and the Pratt and Wilson wet-chemical methods. *Hyperfine Interact.* **117,** 175–204; 10.1023/A:1012607813487 (1998).

6. Rancourt, D. G. Magnetism of Earth, Planetary, and Environmental Nanomaterials. *Rev. Mineral. Geochem.* **44,** 217–292; 10.2138/rmg.2001.44.07 (2001).

7. Thompson, A., Rancourt, D. G., Chadwick, O. A. & Chorover, J. Iron solid-phase differentiation along a redox gradient in basaltic soils. *Geochim. Cosmochim. Acta* **75,** 119–133; 10.1016/j.gca.2010.10.005 (2011).

8. Sun, J. *et al.* Simultaneously Quantifying Ferrihydrite and Goethite in Natural Sediments Using the Method of Standard Additions with X-ray Absorption Spectroscopy. *Chem. Geol.* **476,** 248–259; 10.1016/j.chemgeo.2017.11.021 (2018).

9. Winkler, P. *et al.* Contrasting evolution of iron phase composition in soils exposed to redox fluctuations. *Geochim. Cosmochim. Acta* **235,** 89–102; 10.1016/j.gca.2018.05.019 (2018).

10. Chen, C., Meile, C., Wilmoth, J., Barcellos, D. & Thompson, A. Influence of pO2 on Iron Redox Cycling and Anaerobic Organic Carbon Mineralization in a Humid Tropical Forest Soil. *Environ. Sci. Technol.* **52,** 7709–7719; 10.1021/acs.est.8b01368 (2018).

11. Murad, E. Clays and clay minerals: What can Mössbauer spectroscopy do to help understand them? *Hyperfine Interact.* **117,** 39–70; 10.1023/A:1012635124874 (1998).

12. Murad, E. & Wagner, U. Mössbauer study of pure illite and its firing products. *Hyperfine Interact.* **91,** 685–688; 10.1007/BF02064591 (1994).

13. Michel, F. M. *et al.* Ordered ferrimagnetic form of ferrihydrite reveals links among structure, composition, and magnetism. *Proc. Natl. Acad. Sci. U.S.A.* **107,** 2787–2792; 10.1073/pnas.0910170107 (2010).
